# Effect of post-traumatic stress disorder on type 2 diabetes and the mediated effect of obesity: a Mendelian randomization study

**DOI:** 10.3389/fendo.2024.1375068

**Published:** 2024-09-05

**Authors:** Yunfeng Yu, Gang Hu, Xinyu Yang, Siyang Bai, Jingyi Wu, Keke Tong, Rong Yu

**Affiliations:** ^1^ Department of Endocrinology, The First Hospital of Hunan University of Chinese Medicine, Changsha, Hunan, China; ^2^ School of Traditional Chinese Medicine, Hunan University of Chinese Medicine, Changsha, Hunan, China; ^3^ The Third School of Clinical Medicine, Zhejiang Chinese Medical University, Hangzhou, Zhejiang, China; ^4^ The Affiliated Changde Hospital, Hunan University of Chinese Medicine, Changde, Hunan, China

**Keywords:** post-traumatic stress disorder, type 2 diabetes, obesity, hypertension, genetic susceptibility, Mendelian randomization

## Abstract

**Objective:**

Whether the role of post-traumatic stress disorder (PTSD) on type 2 diabetes (T2D) is mediated by obesity or other mediating factors is controversial. This study was designed to assess the impact of PTSD on genetic susceptibility to T2D and mediating factors.

**Methods:**

The datasets for PTSD, T2D, obesity, hypertension, hyperlipidemia, smoking status, and alcohol consumption were obtained from genome-wide association studies. Mendelian randomization (MR) was used to assess exposure-outcome causality, and inverse variance weighted was used as the primary tool for MR analysis. MR-Egger intercept, Cochran’s Q, and leave-one-out sensitivity analysis were employed to assess horizontal pleiotropy, heterogeneity, and robustness, respectively.

**Results:**

The MR analysis showed that PTSD was associated with increased genetic susceptibility to T2D (OR, 1.036; 95% CI, 1.008-1.064; *p* = 0.011), obesity (OR, 1.033; 95% CI, 1.016-1.050; *p* < 0.001), and hypertension (OR, 1.002; 95% CI, 1.000-1.003; *p* = 0.015), but not not with genetic susceptibility to hyperlipidemia, alcohol consumption, and smoking status (*p* ≥ 0.05). Mediated effect analysis showed that PTSD increased genetic susceptibility to T2D by increasing genetic susceptibility to obesity and hypertension, with obesity accounting for 9.51% and hypertension accounting for 2.09%. MR-Egger intercept showed no horizontal pleiotropy (*p* ≥ 0.05). Cochran’s Q showed no heterogeneity (*p* ≥ 0.05). Leave-one-out sensitivity analysis showed that the results were robust.

**Conclusion:**

This MR analysis suggests that PTSD increases the risk of T2D and that this effect is partially mediated by obesity and hypertension. Active prevention and treatment of PTSD can help reduce the risk of T2D.

## 1 Introduction

Post-traumatic stress disorder (PTSD) is a mental health condition that occurs after exposure to a traumatic event (1). Epidemiological research has shown that the annual prevalence of PTSD in Europe ranges from 1% to 3% (2), while the lifetime prevalence in the United States is reported as 8.3% (3). Typical symptoms of PTSD include re-experiencing traumatic intrusive memories, avoidance of memories of traumatic events, negative cognitions and emotions, and hyperarousal, significantly impacting the physical and mental health as well as the quality of life of affected individuals (4). Risk factors for PTSD include gender (with females at higher risk), economic hardship, lower education level, unemployment, exposure to traumatic events, and co-morbid psychological disorders (5), and effective management of these factors contributes to reducing the risk of morbidity. Although psychotherapy as a primary treatment for PTSD has improved the prognosis of some patients, its efficacy remains limited (6). As studies have progressed, more and more researchers have found that PTSD may be a metabolic disorder in disguise, increasing the risk of conditions such as obesity, dyslipidemia, and hypertension (7). Given that obesity, hyperlipidemia and hypertension are independent risk factors for type 2 diabetes (T2D) (8), there is a growing interest in understanding whether PTSD could be a risk factor for T2D.

Type 2 diabetes is a chronic metabolic disease characterized by insulin resistance and hyperinsulinemia (9). Globally, approximately 537 million adults were reported to have diabetes in 2021, and the number continues to rise (10, 11). As an incurable disease, T2D is one of the leading causes of increased mortality and disability worldwide (12). A relevant study suggested that PTSD is a potential risk factor for T2D, which increases the incidence of T2D by 49% (13). However, considering that obesity is an important risk factor for T2D, other studies suggested that this effect is mediated by obesity (14). They believe that, after adjusting for obesity or BMI, the association between PTSD and T2D was no longer significant (15). Is PTSD an independent risk factor for T2D? Is obesity an intermediate pathway for PTSD to act on T2D? These questions continue to plague clinicians and researchers and await new analytical approaches to be addressed.

Mendelian randomization (MR) is a genetic statistical method for assessing the causal effect of exposure variables and outcome variables (16). MR is less susceptible to confounding variables and reverse causality due to the randomized nature of genetics (17), and it has emerged as an effective alternative to traditional epidemiologic studies (18). This MR analysis explored the causal relationship of PTSD with T2D and obesity in terms of genetic prediction, aiming to investigate whether the effect of PTSD on T2D is independent of obesity.

## 2 Materials and methods

### 2.1 Study design

MR was based on three basic assumptions of association, independence and exclusivity (19). The association assumption required that single nucleotide polymorphisms (SNPs) were strongly correlated with exposure factors. The independence assumption required that SNPs were independent of confounding variables. The exclusivity assumption required that SNPs only acted on outcome variables through exposure variables and not through other pathways.

**Figure 1:**
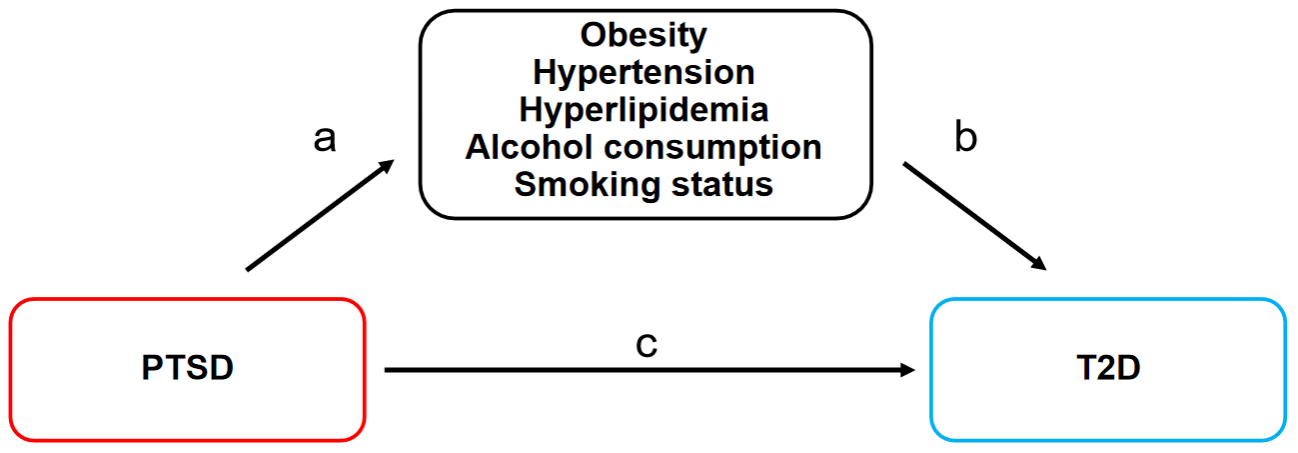
MR design for the causal relationships between PTSD, T2D, and mediating factors. MR, Mendelian randomization; PTSD, post-traumatic stress disorder; T2D, type 2 diabetes. **(A)** The causal effects of PTSD on mediating factors. **(B)** The causal effects of mediating factors on T2D. **(C)** The causal effect of PTSD on T2D.

Firstly, adhering to basic assumptions, a two-sample MR analysis was employed to assess the causal effect of PTSD on T2D (c). Secondly, MR was employed to assess the causal effects of PTSD on mediating factors such as obesity, hypertension, hyperlipidemia, alcohol consumption, and smoking status (a). Subsequently, mediating factors exhibiting significant causal relationships with PTSD were further examined for their causal effects on T2D (b). Finally, mediated MR was employed to evaluate the mediated effects of these mediating factors in the pathway from PTSD to T2D. The MR design is shown in **Figure 1**.

### 2.2 Data sources

The datasets were obtained from FinnGen (www.finngen.fi/fi), UK Biobank (www.nealelab.is/uk-biobank), European Bioinformatics Institute (www.ebi.ac.uk), and IEU Open GWAS project (gwas.mrcieu.ac.uk/), as shown in **Table 1**. As these datasets were publicly available, the study did not require additional ethical approval.

**Table 1:** Details of the GWAS studies included in the Mendelian randomization.

Year	Trait	GWAS ID	Population	Sample size	Web source
2024	PTSD	finngen_R11_F5_PTSD	European	406,822	www.finngen.fi/en
2018	T2D	ebi-a-GCST005413	European	70,127	https://www.ebi.ac.uk
2024	Obesity	finngen_R11_E4_OBESITY	European	453,592	www.finngen.fi/en
2018	Hypertension	ukb-b-14057	European	462,933	www.nealelab.is/uk-biobank
2021	Hyperlipidemia	ebi-a-GCST90104006	European	349,222	https://www.ebi.ac.uk
2022	Alcohol consumption	ieu-b-4834	European	83,626	gwas.mrcieu.ac.uk/
2018	Smoking status	ebi-a-GCST90029014	European	468,170	https://www.ebi.ac.uk

PTSD, post-traumatic stress disorder; T2D, type 2 diabetes.

The dataset of PTSD, numbered finngen_R11_F5_PTSD, provided genome-wide association studies (GWAS) data of 406,822 Europeans, including 3,005 cases in the experimental group and 403,817 cases in the control group. PTSD was diagnosed using the Post-traumatic Stress Disorder Checklist (PCL), which was described in FinnGen as an anxiety disorder precipitated by an experience of intense fear or horror while exposed to a traumatic (especially life-threatening) event. The disorder was characterized by intrusive recurring thoughts or images of the traumatic event; avoidance of anything associated with the event; a state of hyperarousal and diminished emotional responsiveness. These symptoms were present for at least one month, and the disorder was usually long-term. In addition, the comorbidity rates of PTSD with other psychiatric disorders were as follows: major depressive disorder 3.95%, anxiety disorders 5.91%, bipolar affective disorders 3.59%, sleep disorder 2.59%, personality disorders 5.32%, disturbance of activity and attention 2.91%, obsessive-compulsive disorder 2.74%, schizophrenia 1.07%.

The dataset of T2D, numbered ebi-a-GCST005413, provided GWAS data of 70,127 Europeans, including 12,931 cases in the experimental group and 57,196 cases in the control group. T2D is defined as a type of diabetes mellitus characterized by insulin resistance or desensitization and increased blood glucose levels.

The dataset of obesity, numbered finngen_R11_E4_OBESITY, contained GWAS data of 453,592 Europeans, including 27,711 cases in the experimental group and 425,881 cases in the control group. Obesity is defined as a disorder involving an excessive amount of body fat.

The dataset of hypertension, numbered ukb-b-14057, contained GWAS data of 462,933 Europeans, including 119,731 cases in the experimental group and 343,202 cases in the control group. It originated from a questionnaire survey conducted by the UK Biobank between 2006 and 2010, analyzing self-reported hypertension among participants globally.

The dataset of hyperlipidemia, numbered ebi-a-GCST90104006, contained GWAS data of 349,222 Europeans, including 39,961 cases in the experimental group and 309,261 cases in the control group. It was the familial combined hyperlipidemia defined by Consensus criteria, an instance of hyperlipidemia (disease) that is caused by an inherited modification of the individual’s genome.

The dataset of alcohol consumption, numbered ieu-b-4834, contained GWAS data of 83,626 Europeans. It was derived from the statistical analysis of the online 24-hour dietary recall questionnaire.

The dataset of smoking status, numbered ebi-a-GCST90029014, contained GWAS data of 468,170 Europeans. It was derived from the self-reported binary measure and used to denote whether an individual is currently or has ever been a smoker.

### 2.3 Genetic instrumental variables selection

First, the threshold of *p* < 5 × 10^-5^ was applied to search for SNPs closely associated with PTSD, and the threshold of *p* < 5 × 10^-8^ was applied to search for SNPs closely associated with obesity, hypertension, hyperlipidemia, alcohol consumption, and smoking status, to satisfy the association assumption. Second, qualifying *R^2^* < 0.001, *kb* = 10,000, we searched for SNPs with independence to satisfy the independence assumption. Third, limiting *F* > 10, SNPs with strong correlation were searched. *F* = [/()]*[)/]. R^2^ referred to the cumulative explained variance of the selected instrument variables on exposure, N referred to the sample size of the GWAS, and K referred to a number of paired samples. Fourth, the meanings of SNPs were searched through databases, including PhenoScanner, PubMed, and Web of Science, and SNPs that might be associated with the outcome were excluded to satisfy the independence and the exclusivity assumption. Fifth, the allelic orientation of exposure-SNPs and outcome-SNPs was adjusted, and mismatched SNPs were excluded based on the effect of allele frequency. Sixth, outlier SNPs (*p* < 1) were excluded using the MR-Pleiotropy RESidual Sum and Outlier method (MR-PRESSO) to ensure the correctness of causal inference.

### 2.4 Data analysis

The study followed the STROBE-MR guidelines (20). First, a two-sample MR was used to analyze the overall effect of PSTD on T2D (c). Second, a two-step MR was used to further analyze the causal effect of PTSD on mediating factors (a) and the causal effect of mediating factors on T2D (b) to screen for significant mediating factors. Third, the mediated effect and mediated proportion of mediating factors in the action of PTSD to T2D were calculated based on the above data. Mediated effect = a × b, with confidence interval (CI) calculated by the delta method, and mediated proportion = (a × b)/c.

R 4.3.1 software “TwoSampleMR (0.5.7)” was used for the MR analysis to evaluate the causal relationships. Inverse variance weighted (IVW) was the main assessment tool, which allowed for unbiased causal analysis without pleiotropy (21). Weighted median and MR-Egger were secondary assessment tools, with the former being less sensitive to error values and outliers, and the latter providing effective causal analysis in the presence of pleiotropy. When the MR analysis had no heterogeneity and horizontal pleiotropy, only IVW were referenced without weighted median and MR-Egger. When heterogeneity existed in the MR analysis, both IVW and weighted median were referenced. When the MR analysis had horizontal pleiotropy, only MR-Egger was referenced. Subsequently, we employed mediated analysis to assess the mediating ratio and mediating effect of obesity in the relationship between PTSD and T2D. In addition, MR-Egger intercept was used to assess horizontal pleiotropy, with *p* ≥ 0.05 indicating no significant pleiotropy to meet the exclusivity assumption. Cochran’s Q was used to assess heterogeneity, with *p* ≥ 0.05 indicating no significant heterogeneity. Leave-one-out sensitivity analysis was used to assess the robustness of the MR results, indicating that the results were robust when the combined effect sizes were all on the same side.

## 3 Results

### 3.1 Genetic instrumental variables

After step-by-step screening, 94 SNPs for PTSD and T2D, 96 SNPs for PTSD and obesity, 89 SNPs for PTSD and hypertension, 97 SNPs for PTSD and hyperlipidemia, 73 SNPs for PTSD and alcohol consumption, 88 SNPs for PTSD and smoking status, 46 SNPs for obesity and T2D, as well as 204 SNPs for hypertension and T2D were included in the causal analysis, as described in **Supplementary Table S1**.

### 3.2 MR analysis

**Figure 2:**
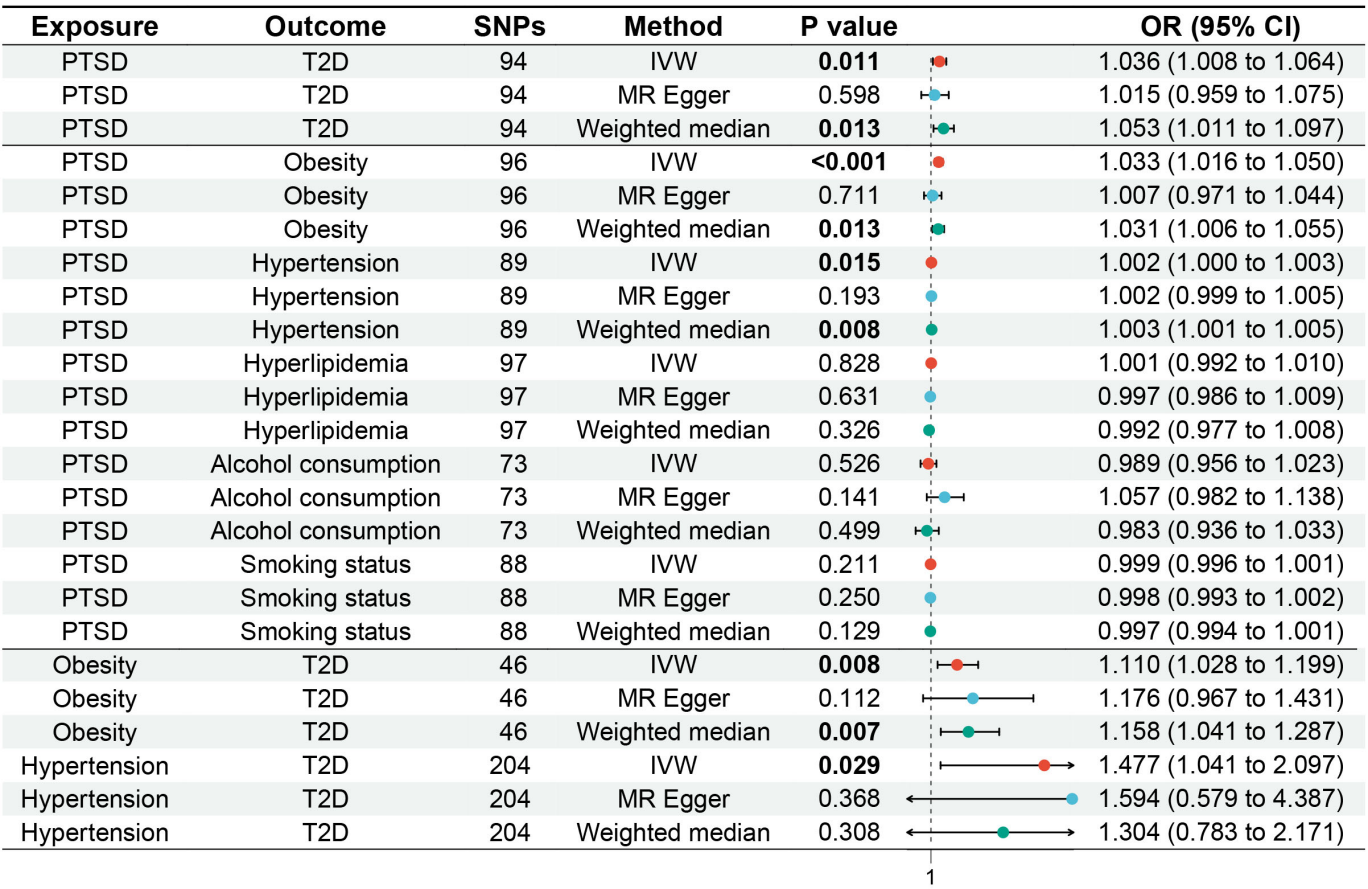
Forest plot of MR analysis for the causal relationships between PTSD, T2D, and mediating factors. MR, Mendelian randomization; PTSD, post-traumatic stress disorder; T2D, type 2 diabetes.

**Figure 3:**
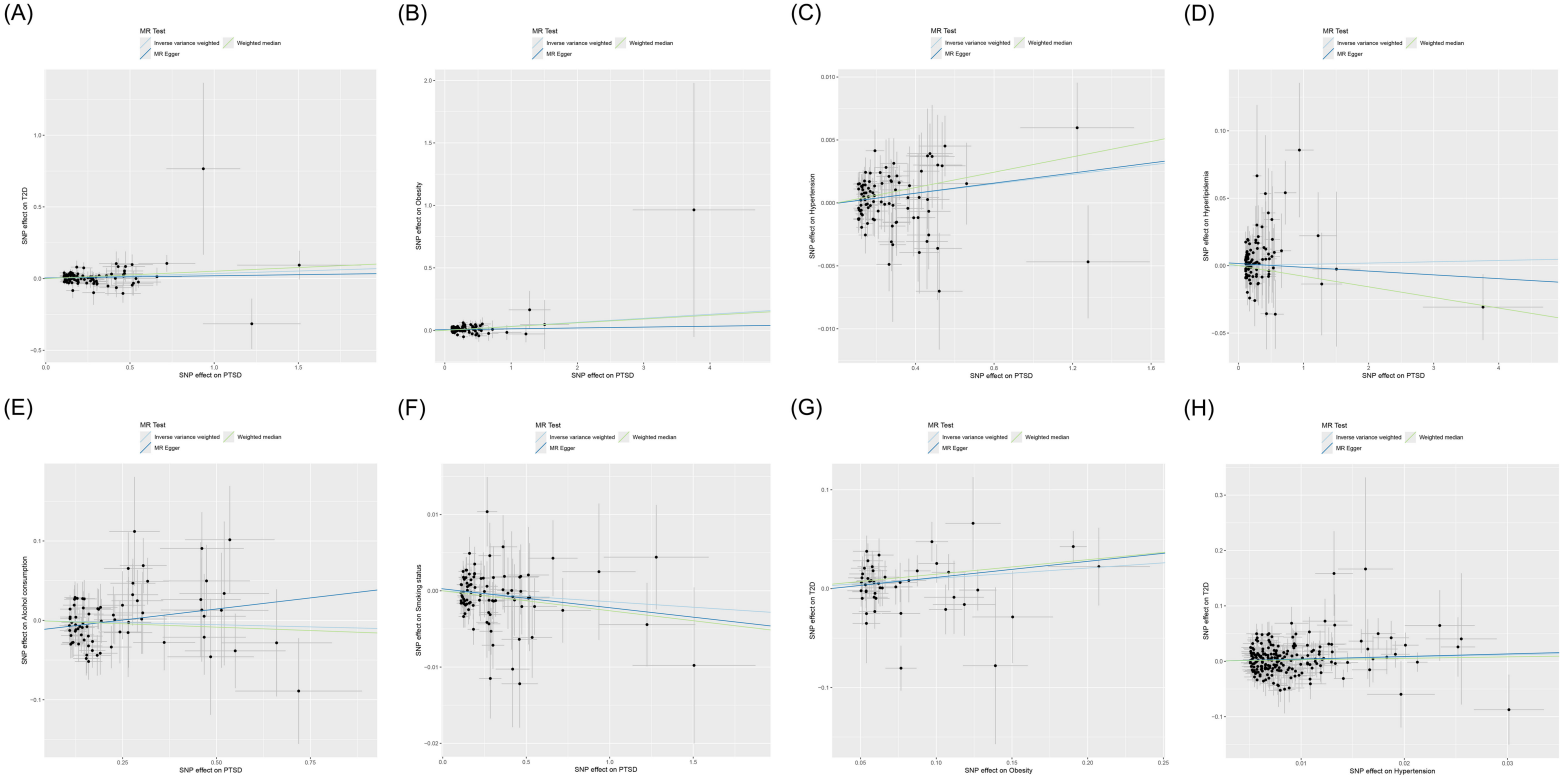
Scatter plot of MR analysis for the causal relationships between PTSD, T2D, and mediating factors. **(A)** PTSD on T2D; **(B)** PTSD on obesity; **(C)** PTSD on hypertension; **(D)** PTSD on hyperlipidemia; **(E)** PTSD on alcohol consumption; **(F)** PTSD on smoking status; **(G)** Obesity on T2D; **(H)** Hypertension on T2D. MR, Mendelian randomization; PTSD, post-traumatic stress disorder; T2D, type 2 diabetes.

**Figure 4:**
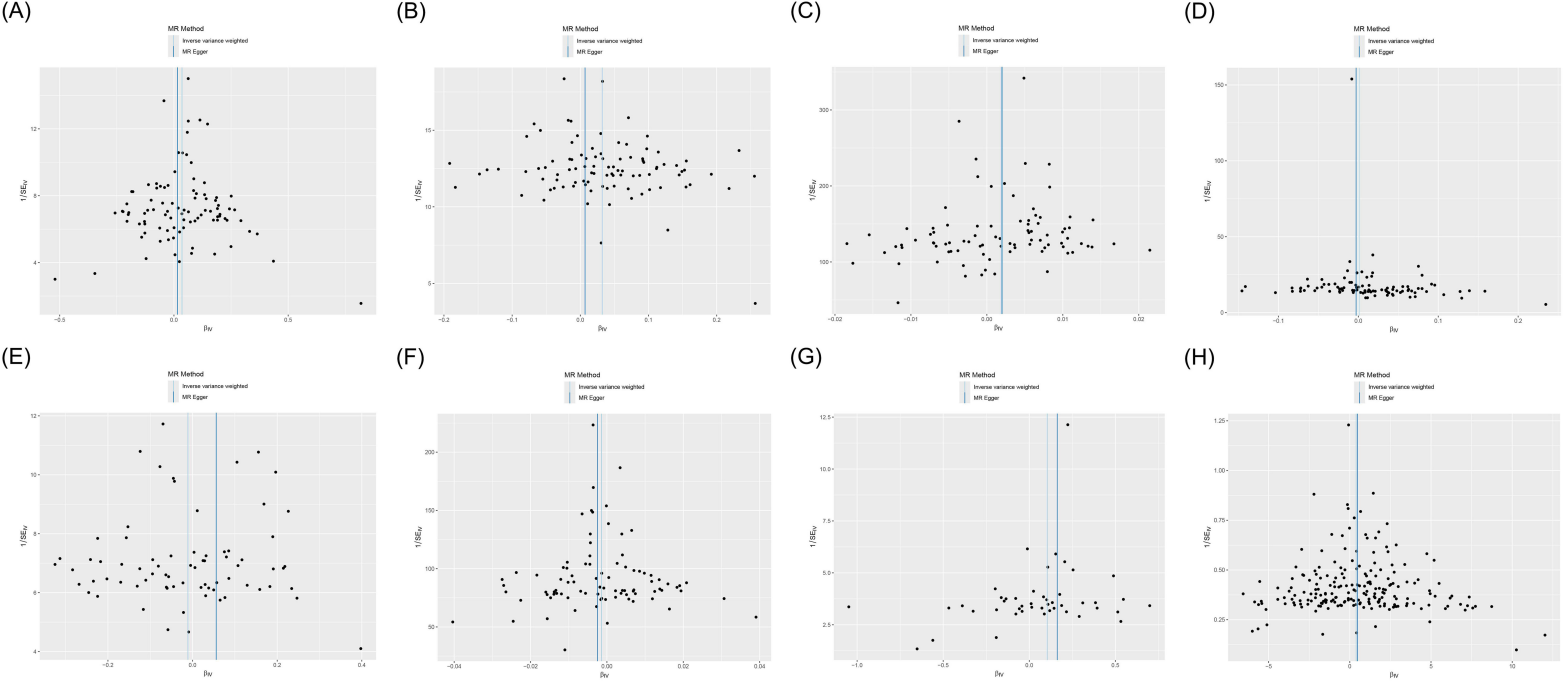
Funnel plot of heterogeneity analysis on the causal relationships between PTSD, T2D, and mediating factors. **(A)** PTSD on T2D; **(B)** PTSD on obesity; **(C)** PTSD on hypertension; **(D)** PTSD on hyperlipidemia; **(E)** PTSD on alcohol consumption; **(F)** PTSD on smoking status; **(G)** Obesity on T2D; **(H)** Hypertension on T2D.

**Figure 5:**
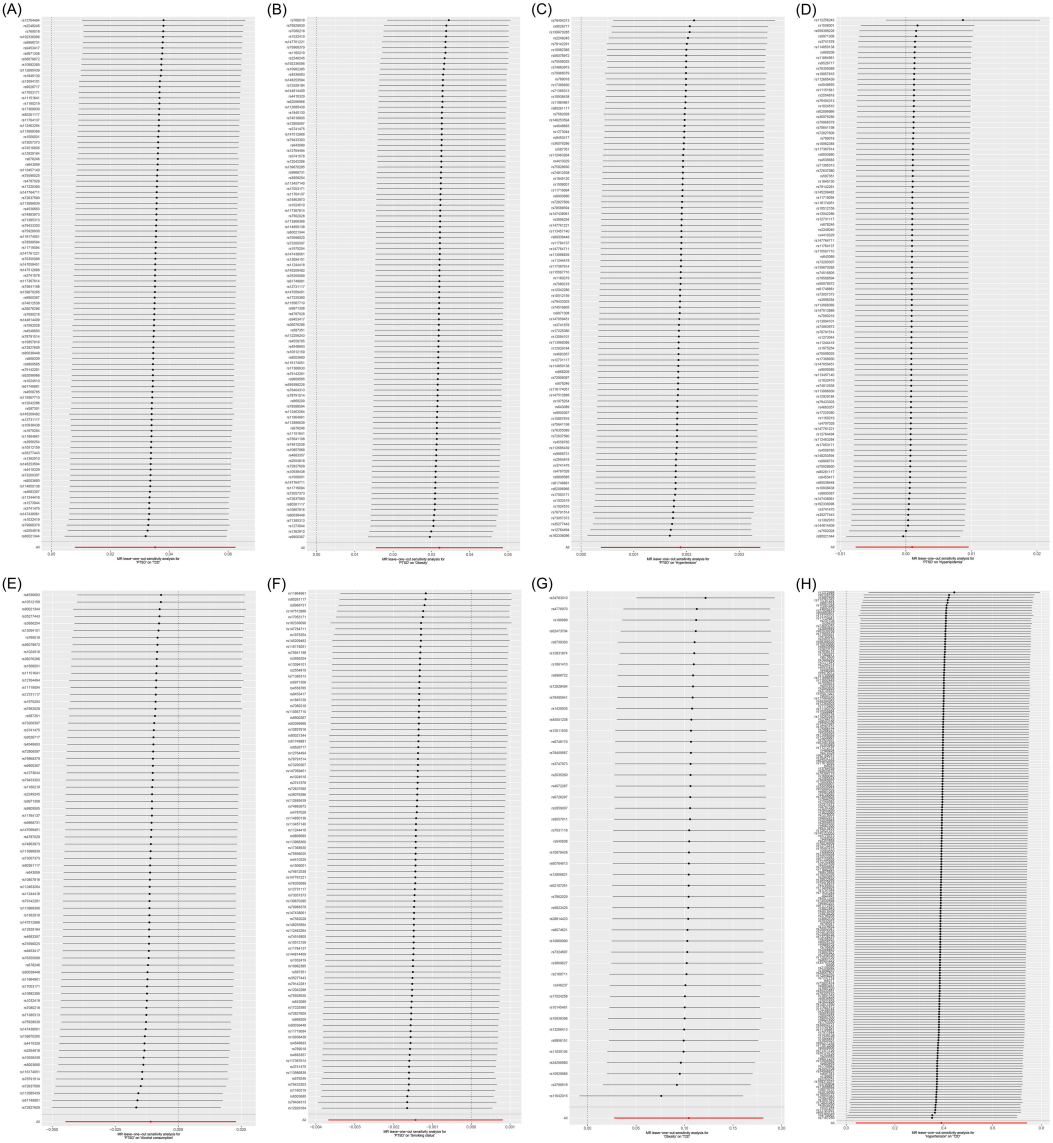
Leave-one-out sensitivity analysis on the causal relationships between PTSD, T2D, and mediating factors. **(A)** PTSD on T2D; **(B)** PTSD on obesity; **(C)** PTSD on hypertension; **(D)** PTSD on hyperlipidemia; **(E)** PTSD on alcohol consumption; **(F)** PTSD on smoking status; **(G)** Obesity on T2D; **(H)** Hypertension on T2D.

MR analysis was employed to assess the causal effect between exposure and outcomes. The corresponding forest plot is illustrated in **Figure 2**, and the scatter plot is depicted in **Figure 3**. MR-Egger intercept analysis is presented in **Supplementary Table S2**. Cochran’s Q test is outlined in **Supplementary Table S3** and **Figure 4**. The results of leave-one-out sensitivity analysis are displayed in **Figure 5**.

#### 3.2.1 Effect of PTSD on T2D

IVW (OR, 1.036; 95% CI, 1.008-1.064; *p* = 0.011) and weighted median (OR, 1.053; 95% CI, 1.011-1.097; *p* = 0.013) showed that PTSD was associated with increased genetic susceptibility to T2D, whereas MR-Egger (OR, 1.015; 95% CI, 0.959-1.075; *p* = 0.598) did not observe this effect, as shown in **Figures 2**, **3A**. MR-Egger intercept showed no significant horizontal pleiotropy (*p* = 0.436), as shown in **Supplementary Table S2**. Cochran’s Q showed no significant heterogeneity (*p* = 0.378), as shown in **Figure 4A** and **Supplementary Table S3**. Leave-one-out sensitivity analysis showed the results were robust, as shown in **Figure 5A**.

#### 3.2.2 Effect of PTSD on mediating factors

IVW showed that PTSD was associated with increased genetic susceptibility to obesity (OR, 1.033; 95% CI, 1.016-1.050; *p* < 0.001) and hypertension (OR, 1.002; 95% CI, 1.000-1.003; *p* = 0.015), but not with genetic susceptibility to hyperlipidemia (OR, 1.001; 95% CI, 0.992-1.010; *p* = 0.828), alcohol consumption (OR, 0.989; 95% CI, 0.956-1.023; *p* = 0.526), and smoking status (OR, 0.999; 95% CI, 0.996-1.001; *p* = 0.211). Additionally, weighted median also showed that PTSD was associated with increased genetic susceptibility to obesity (OR, 1.031; 95% CI, 1.006-1.055; *p* = 0.013) and hypertension (OR, 1.003; 95% CI, 1.001-1.005; *p* = 0.008), while MR-Egger did not report a significant causal effect, as shown in **Figures 2**, **3B–F**. MR-Egger intercept showed no significant horizontal pleiotropy (*p* ≥ 0.05), as shown in **Supplementary Table S2**. Cochran’s Q showed no significant heterogeneity (*p* ≥ 0.05), as shown in **Figures 4B–F** and **Supplementary Table S3**. Leave-one-out sensitivity analysis showed the results were robust, as shown in **Figures 5B–F**.

#### 3.2.3 Effect of mediating factors on T2D

IVW showed that obesity (OR, 1.110; 95% CI, 1.028-1.199; *p* = 0.008) and hypertension (OR, 1.477; 95% CI, 1.041-2.097; *p* = 0.029) were associated with increased genetic susceptibility to T2D. Additionally, weighted median also showed that obesity was associated with increased genetic susceptibility to T2D (OR, 1.158; 95% CI, 1.041-1.287; *p* = 0.007), while MR-Egger did not report a significant causal effect, as shown in **Figures 2**, **3G, H**. MR-Egger intercept showed no significant horizontal pleiotropy (*p* ≥ 0.05), as shown in **Supplementary Table S2**. Cochran’s Q showed no significant heterogeneity (*p* ≥ 0.05) except for the result of hypertension on T2D (*p* = 0.010), as shown in **Figures 4G, H** and **Supplementary Table S3**. Leave-one-out sensitivity analysis showed the results were robust, as shown in **Figures 5G, H**.

#### 3.2.4 Mediated effect of obesity and hypertension

**Figure 6:**
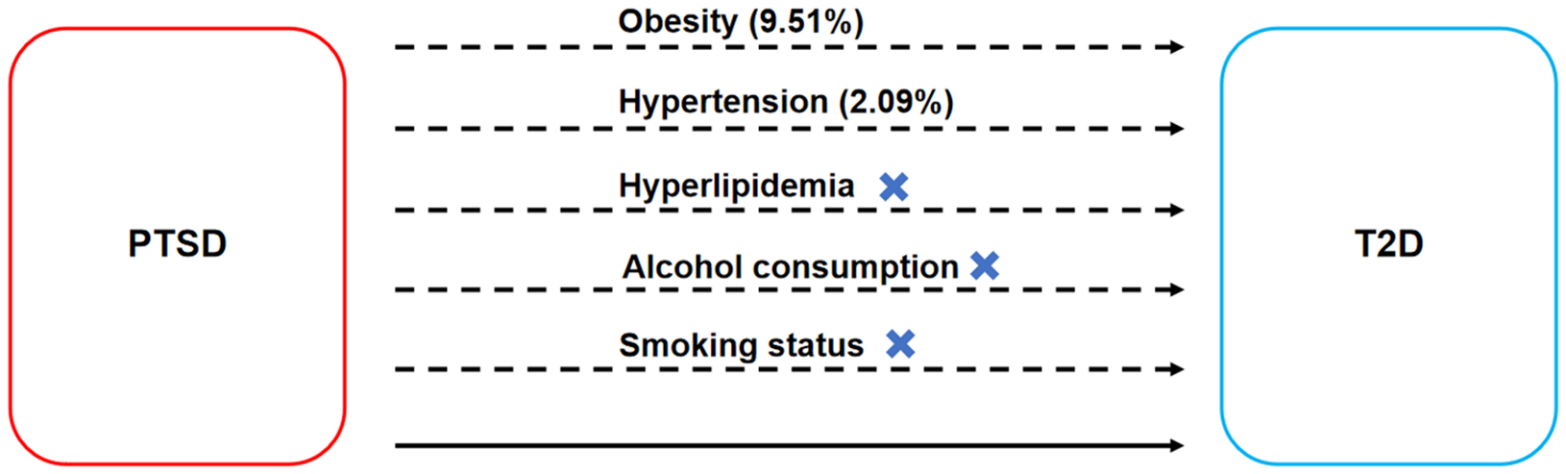
Mediated effect analysis on the causal relationships between PTSD and T2D. The dashed line represents the mediated effect, while the solid line represents the direct effect.

The mediated effect analysis showed that PTSD increased genetic susceptibility to T2D by increasing genetic susceptibility to obesity and hypertension, with obesity accounting for 9.51% of the PTSD-associated increase in genetic susceptibility to T2D (mediated proportion: 9.51%; mediated effect: 0.00335, 95% CI 0.00154-0.00516, *p* < 0.001) and hypertension accounting for 2.09% of the PTSD-associated increase in genetic susceptibility to T2D (mediated proportion: 2.09%; mediated effect: 0.000736, 95% CI 0.000146-0.00133, *p* = 0.015), as shown in **Figure 6**.

## 4 Discussion

Post-traumatic stress disorder is associated with disability and suicidal behavior and poses a significant threat to human physical and mental health, as well as quality of life (22). Earlier studies suggested that PTSD is linked to an increased risk of T2D and may serve as an independent risk factor for T2D (13). However, due to the high comorbidity between PTSD and obesity (23), others have proposed that the elevated risk of T2D might be attributed to PTSD-mediated obesity rather than a direct effect of PTSD (14). Whether PTSD is an independent risk factor for T2D remains a controversial topic. To the best of our knowledge, this is the first MR study to evaluate the impact of PTSD on genetic susceptibility to T2D and obesity. It aimed to investigate whether the role of PTSD in T2D is independent of obesity.

This MR analysis revealed that PTSD increased genetic susceptibility to T2D, and this effect may be achieved by regulating obesity and hypertension. There was no significant horizontal pleiotropy or heterogeneity in these results and they were robust. Since the datasets of this study exclusively comprised individuals of European ancestry, the findings primarily shed light on the effect of PTSD on the risk of T2D in Europeans.

Previous clinical studies demonstrated that PTSD was associated with an elevated prevalence of obesity. In a nationally representative cross-sectional survey in the United States, the prevalence of obesity among individuals with PTSD in the past year was 32.6%, which was significantly higher than the 24.1% prevalence observed among those without a history of PTSD (24). A study conducted by the World Trade Center Health Registry revealed that both the persistent PTSD group (39.5% *vs.* 29.3%) and the intermittent PTSD group (36.6% *vs.* 29.3%) exhibited significantly increased prevalence of obesity compared to the non-PTSD group (25). Additionally, a cohort study of U.S. military personnel and veterans found that PTSD was linked to an increased risk of ≥10% weight gain in multivariate models (OR, 1.44; 95% CI, 1.20-1.73) (26). Bartoli F et al. (27) conducted a meta-analysis of 13 clinical studies and 589,781 subjects, revealing that individuals with PTSD had a 55% increased risk of obesity compared to the general population (OR, 1.55; 95% CI, 1.32-1.82). These pieces of evidence support that PTSD is an independent risk factor for obesity, aligning with the findings of the MR analysis. Considering that obesity is widely acknowledged as a major risk factor for T2D, the concept that PTSD indirectly increases the risk of T2D by increasing obesity risk holds merit. Indeed, a cohort study of veterans by Scherrer JF et al. (28) affirmed that there was no significant association between PTSD and the incidence of T2D after adjusting for obesity. Consequently, they hypothesized that, in the absence of obesity, patients with PTSD would not have an increased risk of T2D (28).

However, our findings suggest that PTSD contributes to an increased genetic susceptibility to T2D through obesity, explaining 9.51% of the variance. Furthermore, PTSD also enhances genetic susceptibility to T2D through hypertension, accounting for 2.09% of the variance. Interestingly, the cumulative impact of obesity and hypertension as mediators was only 11.60%, with smoking status, alcohol consumption, and hyperlipidemia not contributing significantly. This implies that PTSD may be an independent risk factor for T2D. Several previous clinical studies and meta-analysis substantiate this finding. A cohort study of U.S. military personnel showed that PTSD remained significantly associated with an increased risk of diabetes (OR, 2.07; 95% CI, 1.31-3.29) after adjusting for race, gender, age, BMI, education, and mental health status (29). An analysis of a national electronic database of community health services in the Netherlands indicated a significantly higher prevalence of T2D in both male patients with PTSD (APR, 1.40; 95% CI, 1.12-1.76) and female patients with PTSD (APR, 1.22; 95% CI, 0.95-1.56) compared to patients without PTSD (30). A meta-analysis of nine clinical studies by Vancampfort D et al. (13) showed that the PTSD population had a 49% increased risk of T2D compared to the non-affected population (RR, 1.49; 95% CI, 1.17-1.89). Additionally, a cohort study of U.S. females indicated that females with the most PTSD symptoms had an 80% increased risk of T2D compared to females without trauma exposure (HR, 1.80; 95% CI, 1.5-2.1) (31). The researchers of this study concluded that PTSD severity was positively associated with the risk of T2D, with more PTSD symptoms linked to a greater risk of T2D (31). Another German cross-sectional study demonstrated that patients with full PTSD had a substantial 290% increased risk of T2D compared to those without traumatic events (OR, 3.90; 95% CI, 1.61-9.45) (32). These pieces of evidence strongly suggest that PTSD is an independent risk factor for T2D, and that the severity of the condition is positively associated with the risk of T2D.

Consequently, PTSD emerges as an independent risk factor for both obesity and T2D, increasing the risk of T2D both directly and independently, as well as indirectly through obesity. Roberts AL et al. (31) conducted a longitudinal study with a follow-up of up to 22 years that supports our argument. They found that, after adjusting for BMI, PTSD with varying numbers of symptoms remained associated with an increased risk of T2D, although this association was weakened (31). Vaccarino V et al. (15) further noted that the role of PTSD in T2D includes its effects on BMI, lifestyle and metabolic factors. In summary, the role of PTSD in increasing the risk of T2D is multi-pathway and encompasses not only its direct effects on diabetes but also its effects on other risk factors for diabetes.

Current studies suggest that the impact of PTSD on T2D may be related to inflammatory responses and hypothalamic-pituitary-adrenal axis (HPA) dysfunction. Firstly, individuals with PTSD had higher plasma proinflammatory factor levels and exhibited a low systemic proinflammatory state (33). Notably, these pro-inflammatory factors, such as tumor necrosis factor-α and interleukin-6, were associated with an increased risk of insulin resistance and pancreatic β-cell dysfunction (34). Secondly, dysregulation of the HPA axis was identified as one of the mechanisms involved in the development and progression of PTSD (35), and it is similarly implicated in the pathogenesis of T2D. Under normal physiological conditions, the HPA played a role in regulating hormone levels associated with peripheral insulin sensitivity (36). However, under pathological conditions, the HPA was activated by stress, leading to elevated levels of glucocorticoids, which further result in endocrine disorders such as insulin resistance, abdominal obesity, hyperglycemia, etc. (36). As the current evidence is relatively limited, more studies are warranted in the future to explore the intrinsic mechanism of PTSD-mediated T2D.

In the same field of research, the Psychiatric Genomics Consortium (PGC) updated and published the latest GWAS dataset for PTSD in 2024 (37). It reported 95 SNPs significantly associated with PTSD, including 16 core SNPs, namely rs2853999, rs4810485, rs2384572, rs4626350, rs1051792, rs1051792, rs4664313, rs115032094, rs116482870, rs342706, rs72957586, rs805270, rs1054221, rs77874543, rs204993, and rs9461541 (37). However, these SNPs did not overlap with the 87 SNPs we included. We speculate that this difference may be attributed to sample size, population distribution, as well as data processing and quality control. First, the number of participants and their genetic diversity can affect the results. The sample size of the PTSD dataset provided by PGC was 1,222,882 cases, while the sample size of the PTSD dataset provided by FinnGen was only 339,859 cases, which may affect the detection results of SNPs. Second, even within European ancestry, subtle population stratification may lead to differences in genetic associations. FinnGen included primarily individuals from Finland, a more genetically homogeneous group. However, PGC included European ancestry groups from countries such as the United States, Canada, Australia, the United Kingdom, France, Germany, and the Netherlands, which may include more genetic diversity. Third, each institution has independent data processing pathways and quality control measures, which may lead to differences in reported results. Moreover, to further explore the genetic mechanisms contributing to the increased risk of T2D associated with PTSD, we conducted a search for the 87 SNPs included in the study. Unfortunately, these SNPs have not been cited in any publications, suggesting that their role or significance in health and disease might not be well-established or understood yet. More research is needed to explore the roles and significance of these SNPs in the future.

Undoubtedly, this MR analysis has certain limitations. Firstly, as entire datasets were derived from Europeans, the study primarily elucidated the impact of PTSD on genetic susceptibility to obesity and T2D within this ethnic group, and caution should be exercised when extrapolating these findings to other ethnicities. Secondly, since PTSD was derived from pooled GWAS data, the study couldn’t separately analyze the role of different morbidity statuses and various severities of PTSD on outcome variables. Thirdly, the study could not exclude the potential impact of psychotropic drugs on causal effects. Fourthly, potential unrecognized confounding variables between PTSD and outcome variables may exist, posing a risk of bias. Given these limitations, future studies could benefit from improvements. Firstly, there is a need to further promote global research on the human genome to establish a foundation for understanding ethnically diverse genetics. Secondly, the promotion of stratified clinical studies can help explore the effects of PTSD with different morbidity statuses and severities, thereby enriching the data for evidence-based studies.

## 5 Conclusion

This MR analysis suggests that PTSD increases the risk of T2D and that this effect is partially mediated by obesity and hypertension. Proactive prevention and treatment of PTSD can contribute to reducing the risk of T2D. Further research is essential to delve into the genetic mechanisms underlying PTSD in the future.

## Data Availability

The original contributions presented in the study are included in the article/**Supplementary Material**. Further inquiries can be directed to the corresponding author.

## References

[1] Bryant RA. Post-traumatic stress disorder: a state-of-the-art review of evidence and challenges. World Psychiatry. (2019) 18:259–69. doi: 10.1002/wps.20656 PMC6732680 31496089

[2] Nemeroff Charles B. Post-Traumatic Stress Disorder. New York: Oxford University Press (2018). doi: 10.1093/med/9780190259440.003.0008

[3] Kilpatrick DG Resnick HS Milanak ME Miller MW Keyes KM Friedman MJ. National estimates of exposure to traumatic events and PTSD prevalence using DSM-IV and DSM-5 criteria. J Trauma Stress. (2013) 26:537–47. doi: 10.1002/jts.21848 PMC4096796 24151000

[4] Qi W Gevonden M Shalev A. Prevention of post-traumatic stress disorder after trauma: current evidence and future directions. Curr Psychiatry Rep. (2016) 18:20. doi: 10.1007/s11920-015-0655-0 26800995 PMC4723637

[5] El Hajj M. Prevalence and associated factors of post-traumatic stress disorder in Lebanon: A literature review. Asian J Psychiatr. (2021) 63:102800. doi: 10.1016/j.ajp.2021.102800 34340165

[6] Mohamed A Touheed S Ahmed M Hor M Fatima S. The efficacy of psychedelic-assisted therapy in managing post-traumatic stress disorder (PTSD): A new frontier? Cureus. (2022) 14:e30919. doi: 10.7759/cureus.30919 36465766 PMC9710723

[7] Rosenbaum S Stubbs B Ward PB Steel Z Lederman O Vancampfort D. The prevalence and risk of metabolic syndrome and its components among people with posttraumatic stress disorder: a systematic review and meta-analysis. Metabolism. (2015) 64:926–33. doi: 10.1016/j.metabol.2015.04.009 25982700

[8] Ismail L Materwala H Al Kaabi J. Association of risk factors with type 2 diabetes: A systematic review. Comput Struct Biotechnol J. (2021) 19:1759–85. doi: 10.1016/j.csbj.2021.03.003 PMC8050730 33897980

[9] Cannata D Fierz Y Vijayakumar A LeRoith D. Type 2 diabetes and cancer: what is the connection? Mt Sinai J Med. (2010) 77:197–213. doi: 10.1002/msj.20167 20309918

[10] Majety P Lozada Orquera FA Edem D Hamdy O. Pharmacological approaches to the prevention of type 2 diabetes mellitus. Front Endocrinol (Lausanne). (2023) 14:1118848. doi: 10.3389/fendo.2023.1118848 36967777 PMC10033948

[11] Magliano DJ Boyko EJ IDF Diabetes Atlas 10th edition scientific committee. IDF DIABETES ATLAS. Brussels: International Diabetes Federation (2021). Available at: http://www.ncbi.nlm.nih.gov/books/NBK581934/. 35914061

[12] Nanda M Sharma R Mubarik S Aashima A Zhang K. Type-2 diabetes mellitus (T2DM): spatial-temporal patterns of incidence, mortality and attributable risk factors from 1990 to 2019 among 21 world regions. Endocrine. (2022) 77:444–54. doi: 10.1007/s12020-022-03125-5 35841511

[13] Vancampfort D Rosenbaum S Ward PB Steel Z Lederman O Lamwaka AV. Type 2 diabetes among people with posttraumatic stress disorder: Systematic review and meta-analysis. Psychosom Med. (2016) 78:465–73. doi: 10.1097/PSY.0000000000000297 26867081

[14] Qureshi SU Pyne JM Magruder KM Schulz PE Kunik ME. The link between post-traumatic stress disorder and physical comorbidities: a systematic review. Psychiatr Q. (2009) 80:87–97. doi: 10.1007/s11126-009-9096-4 19291401

[15] Vaccarino V Goldberg J Magruder KM Forsberg CW Friedman MJ Litz BT. Posttraumatic stress disorder and incidence of type-2 diabetes: a prospective twin study. J Psychiatr Res. (2014) 56:158–64. doi: 10.1016/j.jpsychires.2014.05.019 PMC4086302 24950602

[16] Gao RC Sang N Jia CZ Zhang MY Li BH Wei M. Association between sleep traits and rheumatoid arthritis: A mendelian randomization study. Front Public Health. (2022) 10:940161. doi: 10.3389/fpubh.2022.940161 35844889 PMC9280285

[17] Julian TH Boddy S Islam M Kurz J Whittaker KJ Moll T. A review of Mendelian randomization in amyotrophic lateral sclerosis. Brain. (2022) 145:832–42. doi: 10.1093/brain/awab420 PMC9050546 34791088

[18] Ren F Jin Q Liu T Ren X Zhan Y. Causal effects between gut microbiota and IgA nephropathy: a bidirectional Mendelian randomization study. Front Cell Infect Microbiol. (2023) 13:1171517. doi: 10.3389/fcimb.2023.1171517 37201114 PMC10185820

[19] Davies NM Holmes MV Davey Smith G. Reading Mendelian randomisation studies: a guide, glossary, and checklist for clinicians. BMJ. (2018) 362:k601. doi: 10.1136/bmj.k601 30002074 PMC6041728

[20] Burgess S Butterworth A Thompson SG. Mendelian randomization analysis with multiple genetic variants using summarized data. Genet Epidemiol. (2013) 37:658–65. doi: 10.1002/gepi.21758 PMC4377079 24114802

[21] 1000 Genomes Project Consortium Auton A Brooks LD Durbin RM Garrison EP Kang HM. A global reference for human genetic variation. Nature. (2015) 526:68–74. doi: 10.1038/nature15393 26432245 PMC4750478

[22] Sareen J Cox BJ Stein MB Afifi TO Fleet C Asmundson GJG. Physical and mental comorbidity, disability, and suicidal behavior associated with posttraumatic stress disorder in a large community sample. Psychosom Med. (2007) 69:242–8. doi: 10.1097/PSY.0b013e31803146d8 17401056

[23] Michopoulos V Vester A Neigh G. Posttraumatic stress disorder: A metabolic disorder in disguise? Exp Neurol. (2016) 284:220–9. doi: 10.1016/j.expneurol.2016.05.038 PMC5056806 27246996

[24] Pagoto SL Schneider KL Bodenlos JS Appelhans BM Whited MC Ma Y. Association of post-traumatic stress disorder and obesity in a nationally representative sample. Obes (Silver Spring). (2012) 20:200–5. doi: 10.1038/oby.2011.318 22016096

[25] Takemoto E Van Oss KR Chamany S Brite J Brackbill R. Post-traumatic stress disorder and the association with overweight, obesity, and weight change among individuals exposed to the World Trade Center disaster, 2003-2016. Psychol Med. (2021) 51:2647–56. doi: 10.1017/S0033291720001208 32375911

[26] LeardMann CA Woodall KA Littman AJ Jacobson IG Boyko EJ Smith B. Post-traumatic stress disorder predicts future weight change in the Millennium Cohort Study. Obes (Silver Spring). (2015) 23:886–92. doi: 10.1002/oby.21025 25776806

[27] Bartoli F Crocamo C Alamia A Amidani F Paggi E Pini E. Posttraumatic stress disorder and risk of obesity: systematic review and meta-analysis. J Clin Psychiatry. (2015) 76:e1253–1261. doi: 10.4088/JCP.14r09199 26528647

[28] Scherrer JF Salas J Lustman PJ van den Berk-Clark C Schnurr PP Tuerk P. The role of obesity in the association between posttraumatic stress disorder and incident diabetes. JAMA Psychiatry. (2018) 75:1189–98. doi: 10.1001/jamapsychiatry.2018.2028 PMC6248094 30090920

[29] Boyko EJ Jacobson IG Smith B Ryan MAK Hooper TI Amoroso PJ. Risk of diabetes in U.S. military service members in relation to combat deployment and mental health. Diabetes Care. (2010) 33:1771–7. doi: 10.2337/dc10-0296 PMC2909060 20484134

[30] Agyemang C Goosen S Anujuo K Ogedegbe G. Relationship between post-traumatic stress disorder and diabetes among 105,180 asylum seekers in the Netherlands. Eur J Public Health. (2012) 22:658–62. doi: 10.1093/eurpub/ckr138 21953061

[31] Roberts AL Agnew-Blais JC Spiegelman D Kubzansky LD Mason SM Galea S. Posttraumatic stress disorder and incidence of type 2 diabetes mellitus in a sample of women: a 22-year longitudinal study. JAMA Psychiatry. (2015) 72:203–10. doi: 10.1001/jamapsychiatry.2014.2632 PMC4522929 25565410

[32] Lukaschek K Baumert J Kruse J Emeny RT Lacruz ME Huth C. Relationship between posttraumatic stress disorder and type 2 diabetes in a population-based cross-sectional study with 2970 participants. J Psychosom Res. (2013) 74:340–5. doi: 10.1016/j.jpsychores.2012.12.011 23497837

[33] von Känel R Hepp U Kraemer B Traber R Keel M Mica L. Evidence for low-grade systemic proinflammatory activity in patients with posttraumatic stress disorder. J Psychiatr Res. (2007) 41:744–52. doi: 10.1016/j.jpsychires.2006.06.009 16901505

[34] Peruzzolo TL Pinto JV Roza TH Shintani AO Anzolin AP Gnielka V. Inflammatory and oxidative stress markers in post-traumatic stress disorder: a systematic review and meta-analysis. Mol Psychiatry. (2022) 27:3150–63. doi: 10.1038/s41380-022-01564-0 35477973

[35] Morris MC Compas BE Garber J. Relations among posttraumatic stress disorder, comorbid major depression, and HPA function: a systematic review and meta-analysis. Clin Psychol Rev. (2012) 32:301–15. doi: 10.1016/j.cpr.2012.02.002 PMC3340453 22459791

[36] Björntorp P Holm G Rosmond R. Hypothalamic arousal, insulin resistance and Type 2 diabetes mellitus. Diabetes Med. (1999) 16:373–83. doi: 10.1046/j.1464-5491.1999.00067.x 10342336

[37] Nievergelt CM Maihofer AX Atkinson EG. Genome-wide association analyses identify 95 risk loci and provide insights into the neurobiology of post-traumatic stress disorder. Nat Genet. (2024) 56:792–808. doi: 10.1038/s41588-024-01707-9 38637617 PMC11396662

