# Supplementary material for: Effect of post-traumatic stress disorder on type 2 diabetes and the mediated effect of obesity: a Mendelian randomization study
Source: Front Endocrinol (Lausanne). 2024 Sep 5;15:1375068. doi: 10.3389/fendo.2024.1375068 (PMC11410705; doi:10.3389/fendo.2024.1375068)
Supplement: Supplementary file 1 [file Table1.docx]

**Supplementary Table S1.1** Information of identified SNPs in exposure (PTSD) and outcomes (T2D).

|  |  | | | **Exposure (PTSD)** | | |  | **Outcome (T2D)** | | | | |
| --- | --- | --- | --- | --- | --- | --- | --- | --- | --- | --- | --- | --- |
|  | **SNP** | **EA** | **OA** | **β** | **SE** | ***p* value** |  | **Case** | **Control** | **β** | **SE** | ***p* value** |
| 1 | rs1024510 | G | A | -0.219903 | 0.0538123 | 4.38E-05 |  | 12,931 | 57,196 | -0.0191 | 0.0244 | 0.4329 |
| 2 | rs1032419 | C | T | -0.145257 | 0.0355232 | 4.33E-05 |  | 12,931 | 57,196 | -0.0386 | 0.0203 | 0.0567296 |
| 3 | rs10512159 | A | G | 0.159327 | 0.0366852 | 1.40E-05 |  | 12,931 | 57,196 | 0.0319 | 0.0235 | 0.1745 |
| 4 | rs10857816 | G | A | 0.109934 | 0.0261106 | 2.55E-05 |  | 12,931 | 57,196 | 0.0115 | 0.0165 | 0.486 |
| 5 | rs10938438 | C | T | 0.129063 | 0.0294969 | 1.21E-05 |  | 12,931 | 57,196 | 0.0182 | 0.0165 | 0.2697 |
| 6 | rs10982385 | G | T | 0.11 | 0.0264619 | 3.23E-05 |  | 12,931 | 57,196 | -0.022 | 0.016 | 0.1677 |
| 7 | rs11151641 | A | G | 0.115256 | 0.0263531 | 1.22E-05 |  | 12,931 | 57,196 | -0.0148 | 0.0166 | 0.374 |
| 8 | rs11244418 | C | T | -0.225322 | 0.0515111 | 1.22E-05 |  | 12,931 | 57,196 | -0.0746 | 0.0384 | 0.0520403 |
| 9 | rs112463264 | T | G | 0.303551 | 0.0734204 | 3.56E-05 |  | 12,931 | 57,196 | -0.0387 | 0.0481 | 0.4213 |
| 10 | rs112685439 | C | T | -0.304907 | 0.062345 | 1.01E-06 |  | 12,931 | 57,196 | 0.034 | 0.0352 | 0.3343 |
| 11 | rs113457140 | C | G | 0.283916 | 0.0666363 | 2.04E-05 |  | 12,931 | 57,196 | -0.0983 | 0.0848 | 0.2468 |
| 12 | rs113968366 | T | C | -0.513073 | 0.125182 | 4.16E-05 |  | 12,931 | 57,196 | 0.0459 | 0.0716 | 0.5213 |
| 13 | rs113986839 | T | G | 0.265891 | 0.0603691 | 1.06E-05 |  | 12,931 | 57,196 | -0.016 | 0.0504 | 0.7502 |
| 14 | rs114850138 | G | A | 0.429768 | 0.0922631 | 3.19E-06 |  | 12,931 | 57,196 | 0.0837 | 0.0579 | 0.1484 |
| 15 | rs115567710 | A | G | 0.418466 | 0.101522 | 3.76E-05 |  | 12,931 | 57,196 | 0.1045 | 0.0844 | 0.2157 |
| 16 | rs1160219 | C | T | 0.142632 | 0.0331717 | 1.71E-05 |  | 12,931 | 57,196 | -0.0164 | 0.02 | 0.4118 |
| 17 | rs116174051 | C | T | 0.535461 | 0.119247 | 7.11E-06 |  | 12,931 | 57,196 | 2.00E-04 | 0.088 | 0.9986 |
| 18 | rs11719094 | T | G | 0.159073 | 0.0369491 | 1.67E-05 |  | 12,931 | 57,196 | 0.003 | 0.0219 | 0.8927 |
| 19 | rs117367614 | T | C | 0.36126 | 0.0838906 | 1.66E-05 |  | 12,931 | 57,196 | 0.0152 | 0.0594 | 0.7975 |
| 20 | rs11764137 | T | C | -0.242849 | 0.0576301 | 2.51E-05 |  | 12,931 | 57,196 | 0.0313 | 0.0376 | 0.4046 |
| 21 | rs11864961 | A | G | -0.183884 | 0.0351212 | 1.64E-07 |  | 12,931 | 57,196 | -0.0801 | 0.045 | 0.0748101 |
| 22 | rs12042286 | A | T | 0.190387 | 0.0428416 | 8.83E-06 |  | 12,931 | 57,196 | 0.0329 | 0.0291 | 0.2584 |
| 23 | rs1273044 | C | T | -0.109143 | 0.0263228 | 3.38E-05 |  | 12,931 | 57,196 | -0.0398 | 0.0191 | 0.0373904 |
| 24 | rs12731117 | T | C | -0.147557 | 0.0342711 | 1.67E-05 |  | 12,931 | 57,196 | -0.0198 | 0.0183 | 0.2797 |
| 25 | rs12764494 | A | G | -0.54991 | 0.134203 | 4.17E-05 |  | 12,931 | 57,196 | 0.0241 | 0.0402 | 0.548399 |
| 26 | rs12929184 | A | G | -0.160228 | 0.0389966 | 3.98E-05 |  | 12,931 | 57,196 | 0.0834 | 0.0533 | 0.1175 |
| 27 | rs13094101 | A | C | -0.182339 | 0.0379614 | 1.56E-06 |  | 12,931 | 57,196 | 0.0139 | 0.0209 | 0.5066 |
| 28 | rs1362910 | G | A | -0.11021 | 0.0269297 | 4.27E-05 |  | 12,931 | 57,196 | -0.0222 | 0.016 | 0.1653 |
| 29 | rs139670285 | T | C | 0.265025 | 0.0645787 | 4.06E-05 |  | 12,931 | 57,196 | 0.0118 | 0.0371 | 0.750601 |
| 30 | rs144814409 | A | G | -0.935522 | 0.21933 | 2.00E-05 |  | 12,931 | 57,196 | -0.766 | 0.5996 | 0.2014 |
| 31 | rs145209482 | A | G | 1.50508 | 0.368393 | 4.40E-05 |  | 12,931 | 57,196 | 0.094 | 0.1003 | 0.3489 |
| 32 | rs147059451 | T | G | 0.264574 | 0.0641234 | 3.69E-05 |  | 12,931 | 57,196 | 0.0063 | 0.0652 | 0.9226 |
| 33 | rs147438061 | A | G | -0.147717 | 0.0336811 | 1.16E-05 |  | 12,931 | 57,196 | -0.0433 | 0.0227 | 0.05633 |
| 34 | rs147512888 | G | A | -0.460906 | 0.108819 | 2.28E-05 |  | 12,931 | 57,196 | -0.0161 | 0.0666 | 0.8085 |
| 35 | rs147761221 | A | G | -0.412908 | 0.0875558 | 2.41E-06 |  | 12,931 | 57,196 | -0.0019 | 0.0924 | 0.9835 |
| 36 | rs147764711 | G | A | 0.284803 | 0.0664584 | 1.82E-05 |  | 12,931 | 57,196 | -0.0347 | 0.0672 | 0.6058 |
| 37 | rs148253594 | C | T | 0.512693 | 0.123652 | 3.38E-05 |  | 12,931 | 57,196 | 0.0976 | 0.0709 | 0.1686 |
| 38 | rs1506001 | A | G | -0.169846 | 0.0384237 | 9.85E-06 |  | 12,931 | 57,196 | 0.007 | 0.02 | 0.7286 |
| 39 | rs17053171 | T | C | 0.289219 | 0.0688782 | 2.68E-05 |  | 12,931 | 57,196 | -0.0218 | 0.0342 | 0.5229 |
| 40 | rs17225380 | A | G | 0.13851 | 0.032863 | 2.50E-05 |  | 12,931 | 57,196 | -0.0039 | 0.0196 | 0.8425 |
| 41 | rs17368930 | A | T | 0.419127 | 0.101686 | 3.76E-05 |  | 12,931 | 57,196 | -0.0634 | 0.0664 | 0.3394 |
| 42 | rs1845130 | T | G | -0.110026 | 0.0266575 | 3.67E-05 |  | 12,931 | 57,196 | 0.0226 | 0.017 | 0.1838 |
| 43 | rs192336096 | A | G | -1.22335 | 0.289787 | 2.43E-05 |  | 12,931 | 57,196 | 0.3153 | 0.1756 | 0.0726206 |
| 44 | rs1975254 | C | T | 0.132761 | 0.0312117 | 2.10E-05 |  | 12,931 | 57,196 | 0.0253 | 0.0203 | 0.2122 |
| 45 | rs2249245 | A | G | -0.155072 | 0.0312091 | 6.74E-07 |  | 12,931 | 57,196 | 0.0287 | 0.0188 | 0.1272 |
| 46 | rs2554818 | A | G | 0.143588 | 0.0328117 | 1.21E-05 |  | 12,931 | 57,196 | 0.036 | 0.018 | 0.0456499 |
| 47 | rs35277443 | A | C | -0.169386 | 0.0348745 | 1.19E-06 |  | 12,931 | 57,196 | -0.0226 | 0.0193 | 0.241 |
| 48 | rs36076286 | G | A | 0.118192 | 0.0285824 | 3.55E-05 |  | 12,931 | 57,196 | 0.0075 | 0.0168 | 0.6541 |
| 49 | rs3741475 | A | G | 0.139951 | 0.0341593 | 4.19E-05 |  | 12,931 | 57,196 | 0.034 | 0.0194 | 0.0796691 |
| 50 | rs3741578 | C | G | -0.297837 | 0.0729471 | 4.45E-05 |  | 12,931 | 57,196 | -0.0111 | 0.0282 | 0.693 |
| 51 | rs3956254 | T | C | -0.154452 | 0.0349614 | 9.97E-06 |  | 12,931 | 57,196 | -0.0295 | 0.0229 | 0.1983 |
| 52 | rs4410329 | A | G | -0.123388 | 0.0284311 | 1.43E-05 |  | 12,931 | 57,196 | -0.0276 | 0.0186 | 0.1373 |
| 53 | rs4536683 | A | G | 0.153157 | 0.0350246 | 1.23E-05 |  | 12,931 | 57,196 | -0.0011 | 0.0203 | 0.9584 |
| 54 | rs4548693 | C | A | -0.123961 | 0.0296464 | 2.90E-05 |  | 12,931 | 57,196 | -0.0114 | 0.0191 | 0.5518 |
| 55 | rs4558785 | T | A | -0.127557 | 0.0299171 | 2.01E-05 |  | 12,931 | 57,196 | -0.0175 | 0.0179 | 0.3276 |
| 56 | rs4683357 | G | A | -0.125441 | 0.0270408 | 3.50E-06 |  | 12,931 | 57,196 | -0.0234 | 0.0159 | 0.1396 |
| 57 | rs4787028 | C | T | 0.114579 | 0.0275383 | 3.17E-05 |  | 12,931 | 57,196 | -0.0043 | 0.0167 | 0.7972 |
| 58 | rs56078972 | G | T | -0.111841 | 0.0262733 | 2.07E-05 |  | 12,931 | 57,196 | 0.0221 | 0.016 | 0.1667 |
| 59 | rs587351 | C | A | -0.112457 | 0.0262745 | 1.87E-05 |  | 12,931 | 57,196 | -0.018 | 0.0159 | 0.2578 |
| 60 | rs61748881 | G | T | -0.31663 | 0.0564936 | 2.09E-08 |  | 12,931 | 57,196 | -0.0199 | 0.0254 | 0.4333 |
| 61 | rs62096966 | G | T | 0.16496 | 0.0396832 | 3.23E-05 |  | 12,931 | 57,196 | 0.0162 | 0.0203 | 0.4232 |
| 62 | rs643089 | G | T | 0.229217 | 0.055854 | 4.06E-05 |  | 12,931 | 57,196 | -0.0059 | 0.0266 | 0.825 |
| 63 | rs678246 | G | T | -0.191238 | 0.0451704 | 2.30E-05 |  | 12,931 | 57,196 | 0.0095 | 0.0253 | 0.705399 |
| 64 | rs7080218 | A | G | -0.120927 | 0.0297889 | 4.92E-05 |  | 12,931 | 57,196 | -0.0087 | 0.0188 | 0.6445 |
| 65 | rs71385313 | G | T | 0.276722 | 0.0677608 | 4.43E-05 |  | 12,931 | 57,196 | -0.0038 | 0.0415 | 0.9276 |
| 66 | rs72637580 | C | T | 0.277385 | 0.0599411 | 3.70E-06 |  | 12,931 | 57,196 | 0.0011 | 0.0294 | 0.9692 |
| 67 | rs72827609 | A | G | -0.461125 | 0.111496 | 3.54E-05 |  | 12,931 | 57,196 | -0.027 | 0.0391 | 0.49 |
| 68 | rs73057373 | T | C | 0.193197 | 0.0471536 | 4.18E-05 |  | 12,931 | 57,196 | -0.0246 | 0.0335 | 0.4632 |
| 69 | rs73200307 | T | C | -0.158719 | 0.0382272 | 3.30E-05 |  | 12,931 | 57,196 | -0.0369 | 0.0243 | 0.1289 |
| 70 | rs74516805 | A | G | 0.369337 | 0.0885093 | 3.01E-05 |  | 12,931 | 57,196 | -0.0522 | 0.0669 | 0.4353 |
| 71 | rs74812538 | C | T | 0.390509 | 0.0918243 | 2.11E-05 |  | 12,931 | 57,196 | 0.0317 | 0.0803 | 0.6934 |
| 72 | rs74863973 | C | T | -0.144354 | 0.0346156 | 3.04E-05 |  | 12,931 | 57,196 | 0.0041 | 0.0244 | 0.8674 |
| 73 | rs75598025 | G | A | 0.520541 | 0.119165 | 1.25E-05 |  | 12,931 | 57,196 | -0.0324 | 0.0858 | 0.7055 |
| 74 | rs7562028 | A | G | -0.360709 | 0.082574 | 1.25E-05 |  | 12,931 | 57,196 | -0.02 | 0.0345 | 0.562999 |
| 75 | rs75641198 | T | C | 0.274826 | 0.0575213 | 1.77E-06 |  | 12,931 | 57,196 | 0.0119 | 0.0419 | 0.777 |
| 76 | rs75928930 | C | T | 0.281701 | 0.0672459 | 2.80E-05 |  | 12,931 | 57,196 | -0.0068 | 0.0525 | 0.8967 |
| 77 | rs76355089 | G | A | 0.249634 | 0.0602865 | 3.46E-05 |  | 12,931 | 57,196 | 0.0062 | 0.0428 | 0.8846 |
| 78 | rs769018 | T | C | 0.190415 | 0.0459608 | 3.43E-05 |  | 12,931 | 57,196 | -0.0342 | 0.0231 | 0.1391 |
| 79 | rs78588594 | C | G | 0.209619 | 0.0486867 | 1.67E-05 |  | 12,931 | 57,196 | -4.00E-04 | 0.0383 | 0.9909 |
| 80 | rs78791514 | T | C | -0.473445 | 0.115499 | 4.15E-05 |  | 12,931 | 57,196 | -0.0844 | 0.105 | 0.4217 |
| 81 | rs79142281 | A | C | -0.467126 | 0.110791 | 2.48E-05 |  | 12,931 | 57,196 | -0.0354 | 0.0468 | 0.4491 |
| 82 | rs79433303 | T | G | 0.660108 | 0.147299 | 7.41E-06 |  | 12,931 | 57,196 | 0.0133 | 0.0624 | 0.8318 |
| 83 | rs79968379 | G | A | -0.467312 | 0.110772 | 2.46E-05 |  | 12,931 | 57,196 | -0.0532 | 0.0373 | 0.1534 |
| 84 | rs80021344 | G | A | 0.718864 | 0.168948 | 2.09E-05 |  | 12,931 | 57,196 | 0.1058 | 0.0585 | 0.0704693 |
| 85 | rs8003680 | T | C | 0.121925 | 0.0277197 | 1.09E-05 |  | 12,931 | 57,196 | 0.0222 | 0.0158 | 0.16 |
| 86 | rs80038448 | T | C | 0.18611 | 0.0403734 | 4.03E-06 |  | 12,931 | 57,196 | 0.016 | 0.0224 | 0.4745 |
| 87 | rs80261117 | A | G | 0.3007 | 0.0671 | 7.42E-06 |  | 12,931 | 57,196 | -0.0178 | 0.0351 | 0.6134 |
| 88 | rs868209 | A | T | -0.168971 | 0.0395824 | 1.96E-05 |  | 12,931 | 57,196 | -0.0156 | 0.0215 | 0.4679 |
| 89 | rs9453417 | A | G | 0.458284 | 0.106145 | 1.58E-05 |  | 12,931 | 57,196 | -0.1035 | 0.0648 | 0.1104 |
| 90 | rs9526717 | G | A | 0.125284 | 0.0270735 | 3.70E-06 |  | 12,931 | 57,196 | -0.0125 | 0.0162 | 0.4401 |
| 91 | rs9809585 | G | A | -0.108248 | 0.026432 | 4.22E-05 |  | 12,931 | 57,196 | -0.0129 | 0.0158 | 0.4145 |
| 92 | rs9900367 | C | T | -0.131089 | 0.0285907 | 4.54E-06 |  | 12,931 | 57,196 | -0.0101 | 0.0288 | 0.726999 |
| 93 | rs9968731 | T | C | -0.124696 | 0.0299353 | 3.11E-05 |  | 12,931 | 57,196 | 0.0257 | 0.0166 | 0.1224 |
| 94 | rs9971308 | G | A | 0.136043 | 0.0323547 | 2.61E-05 |  | 12,931 | 57,196 | -0.0303 | 0.0193 | 0.1166 |

SNP, single nucleotide polymorphism; EA, effect allele; OA, other allele; SE, standard error; PTSD, post-traumatic stress disorder; T2D, type 2 diabetes.

**Supplementary Table S1.2** Information of identified SNPs in exposure (PTSD) and outcomes (Obesity).

|  |  | | | **Exposure (PTSD)** | | |  | **Outcome (Obesity)** | | | | |
| --- | --- | --- | --- | --- | --- | --- | --- | --- | --- | --- | --- | --- |
|  | **SNP** | **EA** | **OA** | **β** | **SE** | ***p* value** |  | **Case** | **Control** | **β** | **SE** | ***p* value** |
| 1 | rs1024510 | G | A | -0.219903 | 0.0538123 | 4.38E-05 |  | 27,711 | 425,881 | -0.0516297 | 0.0249902 | 0.0388284 |
| 2 | rs1032419 | C | T | -0.145257 | 0.0355232 | 4.33E-05 |  | 27,711 | 425,881 | 0.00815953 | 0.0220297 | 0.711094 |
| 3 | rs10512159 | A | G | 0.159327 | 0.0366852 | 1.40E-05 |  | 27,711 | 425,881 | 0.164121 | 0.150756 | 0.276306 |
| 4 | rs10857816 | G | A | 0.109934 | 0.0261106 | 2.55E-05 |  | 27,711 | 425,881 | -0.0364866 | 0.0148346 | 0.0139111 |
| 5 | rs10857889 | T | A | 0.112659 | 0.0264427 | 2.04E-05 |  | 27,711 | 425,881 | 0.0182457 | 0.0173934 | 0.294177 |
| 6 | rs10938438 | C | T | 0.129063 | 0.0294969 | 1.21E-05 |  | 27,711 | 425,881 | -0.0321268 | 0.0336253 | 0.339359 |
| 7 | rs10982385 | G | T | 0.11 | 0.0264619 | 3.23E-05 |  | 27,711 | 425,881 | -0.0262555 | 0.0329128 | 0.425027 |
| 8 | rs11151641 | A | G | 0.115256 | 0.0263531 | 1.22E-05 |  | 27,711 | 425,881 | -0.0252334 | 0.0588743 | 0.668216 |
| 9 | rs112256243 | C | G | 3.75787 | 0.923226 | 4.69E-05 |  | 27,711 | 425,881 | 0.0367423 | 0.0320099 | 0.251034 |
| 10 | rs11244418 | C | T | -0.225322 | 0.0515111 | 1.22E-05 |  | 27,711 | 425,881 | 0.0076871 | 0.0704849 | 0.913155 |
| 11 | rs112463264 | T | G | 0.303551 | 0.0734204 | 3.56E-05 |  | 27,711 | 425,881 | 0.00713222 | 0.00929436 | 0.442861 |
| 12 | rs112685439 | C | T | -0.304907 | 0.062345 | 1.01E-06 |  | 27,711 | 425,881 | 0.0289237 | 0.0143246 | 0.04347 |
| 13 | rs113457140 | C | G | 0.283916 | 0.0666363 | 2.04E-05 |  | 27,711 | 425,881 | 0.0447829 | 0.0244346 | 0.0668375 |
| 14 | rs113968366 | T | C | -0.513073 | 0.125182 | 4.16E-05 |  | 27,711 | 425,881 | -0.012463 | 0.0127716 | 0.329146 |
| 15 | rs113986839 | T | G | 0.265891 | 0.0603691 | 1.06E-05 |  | 27,711 | 425,881 | 0.0227327 | 0.0403691 | 0.573353 |
| 16 | rs114850138 | G | A | 0.429768 | 0.0922631 | 3.19E-06 |  | 27,711 | 425,881 | -0.00840363 | 0.00900467 | 0.35069 |
| 17 | rs115567710 | A | G | 0.418466 | 0.101522 | 3.76E-05 |  | 27,711 | 425,881 | -0.0305161 | 0.00958599 | 0.00145553 |
| 18 | rs1160219 | C | T | 0.142632 | 0.0331717 | 1.71E-05 |  | 27,711 | 425,881 | 0.000844976 | 0.00998461 | 0.932557 |
| 19 | rs116174051 | C | T | 0.535461 | 0.119247 | 7.11E-06 |  | 27,711 | 425,881 | 0.00563267 | 0.0108051 | 0.602157 |
| 20 | rs11719094 | T | G | 0.159073 | 0.0369491 | 1.67E-05 |  | 27,711 | 425,881 | -0.0516297 | 0.0249902 | 0.0388284 |
| 21 | rs117367614 | T | C | 0.36126 | 0.0838906 | 1.66E-05 |  | 27,711 | 425,881 | 0.00815953 | 0.0220297 | 0.711094 |
| 22 | rs11764137 | T | C | -0.242849 | 0.0576301 | 2.51E-05 |  | 27,711 | 425,881 | 0.164121 | 0.150756 | 0.276306 |
| 23 | rs11864961 | A | G | -0.183884 | 0.0351212 | 1.64E-07 |  | 27,711 | 425,881 | -0.0364866 | 0.0148346 | 0.0139111 |
| 24 | rs12042286 | A | T | 0.190387 | 0.0428416 | 8.83E-06 |  | 27,711 | 425,881 | 0.0182457 | 0.0173934 | 0.294177 |
| 25 | rs1273044 | C | T | -0.109143 | 0.0263228 | 3.38E-05 |  | 27,711 | 425,881 | -0.0321268 | 0.0336253 | 0.339359 |
| 26 | rs12731117 | T | C | -0.147557 | 0.0342711 | 1.67E-05 |  | 27,711 | 425,881 | -0.0262555 | 0.0329128 | 0.425027 |
| 27 | rs12764494 | A | G | -0.54991 | 0.134203 | 4.17E-05 |  | 27,711 | 425,881 | -0.0252334 | 0.0588743 | 0.668216 |
| 28 | rs12929184 | A | G | -0.160228 | 0.0389966 | 3.98E-05 |  | 27,711 | 425,881 | 0.0367423 | 0.0320099 | 0.251034 |
| 29 | rs13094101 | A | C | -0.182339 | 0.0379614 | 1.56E-06 |  | 27,711 | 425,881 | 0.0076871 | 0.0704849 | 0.913155 |
| 30 | rs1362910 | G | A | -0.11021 | 0.0269297 | 4.27E-05 |  | 27,711 | 425,881 | 0.00713222 | 0.00929436 | 0.442861 |
| 31 | rs139670285 | T | C | 0.265025 | 0.0645787 | 4.06E-05 |  | 27,711 | 425,881 | 0.0289237 | 0.0143246 | 0.04347 |
| 32 | rs144814409 | A | G | -0.935522 | 0.21933 | 2.00E-05 |  | 27,711 | 425,881 | 0.0447829 | 0.0244346 | 0.0668375 |
| 33 | rs145209482 | A | G | 1.50508 | 0.368393 | 4.40E-05 |  | 27,711 | 425,881 | -0.012463 | 0.0127716 | 0.329146 |
| 34 | rs147059451 | T | G | 0.264574 | 0.0641234 | 3.69E-05 |  | 27,711 | 425,881 | 0.0227327 | 0.0403691 | 0.573353 |
| 35 | rs147438061 | A | G | -0.147717 | 0.0336811 | 1.16E-05 |  | 27,711 | 425,881 | -0.00840363 | 0.00900467 | 0.35069 |
| 36 | rs147512888 | G | A | -0.460906 | 0.108819 | 2.28E-05 |  | 27,711 | 425,881 | -0.0305161 | 0.00958599 | 0.00145553 |
| 37 | rs147761221 | A | G | -0.412908 | 0.0875558 | 2.41E-06 |  | 27,711 | 425,881 | 0.000844976 | 0.00998461 | 0.932557 |
| 38 | rs147764711 | G | A | 0.284803 | 0.0664584 | 1.82E-05 |  | 27,711 | 425,881 | 0.00563267 | 0.0108051 | 0.602157 |
| 39 | rs148253594 | C | T | 0.512693 | 0.123652 | 3.38E-05 |  | 27,711 | 425,881 | -0.0516297 | 0.0249902 | 0.0388284 |
| 40 | rs1506001 | A | G | -0.169846 | 0.0384237 | 9.85E-06 |  | 27,711 | 425,881 | 0.00815953 | 0.0220297 | 0.711094 |
| 41 | rs17053171 | T | C | 0.289219 | 0.0688782 | 2.68E-05 |  | 27,711 | 425,881 | 0.164121 | 0.150756 | 0.276306 |
| 42 | rs17225380 | A | G | 0.13851 | 0.032863 | 2.50E-05 |  | 27,711 | 425,881 | -0.0364866 | 0.0148346 | 0.0139111 |
| 43 | rs17368930 | A | T | 0.419127 | 0.101686 | 3.76E-05 |  | 27,711 | 425,881 | 0.0182457 | 0.0173934 | 0.294177 |
| 44 | rs1845130 | T | G | -0.110026 | 0.0266575 | 3.67E-05 |  | 27,711 | 425,881 | -0.0321268 | 0.0336253 | 0.339359 |
| 45 | rs192336096 | A | G | -1.22335 | 0.289787 | 2.43E-05 |  | 27,711 | 425,881 | -0.0262555 | 0.0329128 | 0.425027 |
| 46 | rs1975254 | C | T | 0.132761 | 0.0312117 | 2.10E-05 |  | 27,711 | 425,881 | -0.0252334 | 0.0588743 | 0.668216 |
| 47 | rs2249245 | A | G | -0.155072 | 0.0312091 | 6.74E-07 |  | 27,711 | 425,881 | 0.0367423 | 0.0320099 | 0.251034 |
| 48 | rs2554818 | A | G | 0.143588 | 0.0328117 | 1.21E-05 |  | 27,711 | 425,881 | 0.0076871 | 0.0704849 | 0.913155 |
| 49 | rs36076286 | G | A | 0.118192 | 0.0285824 | 3.55E-05 |  | 27,711 | 425,881 | 0.00713222 | 0.00929436 | 0.442861 |
| 50 | rs3741475 | A | G | 0.139951 | 0.0341593 | 4.19E-05 |  | 27,711 | 425,881 | 0.0289237 | 0.0143246 | 0.04347 |
| 51 | rs3741578 | C | G | -0.297837 | 0.0729471 | 4.45E-05 |  | 27,711 | 425,881 | 0.0447829 | 0.0244346 | 0.0668375 |
| 52 | rs3956254 | T | C | -0.154452 | 0.0349614 | 9.97E-06 |  | 27,711 | 425,881 | -0.012463 | 0.0127716 | 0.329146 |
| 53 | rs4410329 | A | G | -0.123388 | 0.0284311 | 1.43E-05 |  | 27,711 | 425,881 | 0.0227327 | 0.0403691 | 0.573353 |
| 54 | rs4536683 | A | G | 0.153157 | 0.0350246 | 1.23E-05 |  | 27,711 | 425,881 | -0.00840363 | 0.00900467 | 0.35069 |
| 55 | rs4548693 | C | A | -0.123961 | 0.0296464 | 2.90E-05 |  | 27,711 | 425,881 | -0.0305161 | 0.00958599 | 0.00145553 |
| 56 | rs4558785 | T | A | -0.127557 | 0.0299171 | 2.01E-05 |  | 27,711 | 425,881 | 0.000844976 | 0.00998461 | 0.932557 |
| 57 | rs4683357 | G | A | -0.125441 | 0.0270408 | 3.50E-06 |  | 27,711 | 425,881 | 0.00563267 | 0.0108051 | 0.602157 |
| 58 | rs4787028 | C | T | 0.114579 | 0.0275383 | 3.17E-05 |  | 27,711 | 425,881 | -0.0516297 | 0.0249902 | 0.0388284 |
| 59 | rs559398226 | C | T | 0.565271 | 0.130732 | 1.53E-05 |  | 27,711 | 425,881 | 0.00815953 | 0.0220297 | 0.711094 |
| 60 | rs587351 | C | A | -0.112457 | 0.0262745 | 1.87E-05 |  | 27,711 | 425,881 | 0.164121 | 0.150756 | 0.276306 |
| 61 | rs61748881 | G | T | -0.31663 | 0.0564936 | 2.09E-08 |  | 27,711 | 425,881 | -0.0364866 | 0.0148346 | 0.0139111 |
| 62 | rs62096966 | G | T | 0.16496 | 0.0396832 | 3.23E-05 |  | 27,711 | 425,881 | 0.0182457 | 0.0173934 | 0.294177 |
| 63 | rs643089 | G | T | 0.229217 | 0.055854 | 4.06E-05 |  | 27,711 | 425,881 | -0.0321268 | 0.0336253 | 0.339359 |
| 64 | rs678246 | G | T | -0.191238 | 0.0451704 | 2.30E-05 |  | 27,711 | 425,881 | -0.0262555 | 0.0329128 | 0.425027 |
| 65 | rs7080218 | A | G | -0.120927 | 0.0297889 | 4.92E-05 |  | 27,711 | 425,881 | -0.0252334 | 0.0588743 | 0.668216 |
| 66 | rs71385313 | G | T | 0.276722 | 0.0677608 | 4.43E-05 |  | 27,711 | 425,881 | 0.0367423 | 0.0320099 | 0.251034 |
| 67 | rs72637580 | C | T | 0.277385 | 0.0599411 | 3.70E-06 |  | 27,711 | 425,881 | 0.0076871 | 0.0704849 | 0.913155 |
| 68 | rs72806097 | T | C | 0.484445 | 0.115027 | 2.54E-05 |  | 27,711 | 425,881 | 0.00713222 | 0.00929436 | 0.442861 |
| 69 | rs72827609 | A | G | -0.461125 | 0.111496 | 3.54E-05 |  | 27,711 | 425,881 | 0.0289237 | 0.0143246 | 0.04347 |
| 70 | rs73057373 | T | C | 0.193197 | 0.0471536 | 4.18E-05 |  | 27,711 | 425,881 | 0.0447829 | 0.0244346 | 0.0668375 |
| 71 | rs73200307 | T | C | -0.158719 | 0.0382272 | 3.30E-05 |  | 27,711 | 425,881 | -0.012463 | 0.0127716 | 0.329146 |
| 72 | rs74516805 | A | G | 0.369337 | 0.0885093 | 3.01E-05 |  | 27,711 | 425,881 | 0.0227327 | 0.0403691 | 0.573353 |
| 73 | rs74812538 | C | T | 0.390509 | 0.0918243 | 2.11E-05 |  | 27,711 | 425,881 | -0.00840363 | 0.00900467 | 0.35069 |
| 74 | rs74863973 | C | T | -0.144354 | 0.0346156 | 3.04E-05 |  | 27,711 | 425,881 | -0.0305161 | 0.00958599 | 0.00145553 |
| 75 | rs75598025 | G | A | 0.520541 | 0.119165 | 1.25E-05 |  | 27,711 | 425,881 | 0.000844976 | 0.00998461 | 0.932557 |
| 76 | rs7562028 | A | G | -0.360709 | 0.082574 | 1.25E-05 |  | 27,711 | 425,881 | 0.00563267 | 0.0108051 | 0.602157 |
| 77 | rs75641198 | T | C | 0.274826 | 0.0575213 | 1.77E-06 |  | 27,711 | 425,881 | -0.0516297 | 0.0249902 | 0.0388284 |
| 78 | rs75928930 | C | T | 0.281701 | 0.0672459 | 2.80E-05 |  | 27,711 | 425,881 | 0.00815953 | 0.0220297 | 0.711094 |
| 79 | rs76355089 | G | A | 0.249634 | 0.0602865 | 3.46E-05 |  | 27,711 | 425,881 | 0.164121 | 0.150756 | 0.276306 |
| 80 | rs76404313 | G | A | 1.27887 | 0.314601 | 4.80E-05 |  | 27,711 | 425,881 | -0.0364866 | 0.0148346 | 0.0139111 |
| 81 | rs769018 | T | C | 0.190415 | 0.0459608 | 3.43E-05 |  | 27,711 | 425,881 | 0.0182457 | 0.0173934 | 0.294177 |
| 82 | rs78588594 | C | G | 0.209619 | 0.0486867 | 1.67E-05 |  | 27,711 | 425,881 | -0.0321268 | 0.0336253 | 0.339359 |
| 83 | rs78791514 | T | C | -0.473445 | 0.115499 | 4.15E-05 |  | 27,711 | 425,881 | -0.0262555 | 0.0329128 | 0.425027 |
| 84 | rs79142281 | A | C | -0.467126 | 0.110791 | 2.48E-05 |  | 27,711 | 425,881 | -0.0252334 | 0.0588743 | 0.668216 |
| 85 | rs79433303 | T | G | 0.660108 | 0.147299 | 7.41E-06 |  | 27,711 | 425,881 | 0.0367423 | 0.0320099 | 0.251034 |
| 86 | rs79968379 | G | A | -0.467312 | 0.110772 | 2.46E-05 |  | 27,711 | 425,881 | 0.0076871 | 0.0704849 | 0.913155 |
| 87 | rs80021344 | G | A | 0.718864 | 0.168948 | 2.09E-05 |  | 27,711 | 425,881 | 0.00713222 | 0.00929436 | 0.442861 |
| 88 | rs8003680 | T | C | 0.121925 | 0.0277197 | 1.09E-05 |  | 27,711 | 425,881 | 0.0289237 | 0.0143246 | 0.04347 |
| 89 | rs80038448 | T | C | 0.18611 | 0.0403734 | 4.03E-06 |  | 27,711 | 425,881 | 0.0447829 | 0.0244346 | 0.0668375 |
| 90 | rs80261117 | A | G | 0.3007 | 0.0671 | 7.42E-06 |  | 27,711 | 425,881 | -0.012463 | 0.0127716 | 0.329146 |
| 91 | rs868209 | A | T | -0.168971 | 0.0395824 | 1.96E-05 |  | 27,711 | 425,881 | 0.0227327 | 0.0403691 | 0.573353 |
| 92 | rs9453417 | A | G | 0.458284 | 0.106145 | 1.58E-05 |  | 27,711 | 425,881 | -0.00840363 | 0.00900467 | 0.35069 |
| 93 | rs9809585 | G | A | -0.108248 | 0.026432 | 4.22E-05 |  | 27,711 | 425,881 | -0.0305161 | 0.00958599 | 0.00145553 |
| 94 | rs9900367 | C | T | -0.131089 | 0.0285907 | 4.54E-06 |  | 27,711 | 425,881 | 0.000844976 | 0.00998461 | 0.932557 |
| 95 | rs9968731 | T | C | -0.124696 | 0.0299353 | 3.11E-05 |  | 27,711 | 425,881 | 0.00563267 | 0.0108051 | 0.602157 |
| 96 | rs9971308 | G | A | 0.136043 | 0.0323547 | 2.61E-05 |  | 27,711 | 425,881 | -0.0516297 | 0.0249902 | 0.0388284 |

SNP, single nucleotide polymorphism; EA, effect allele; OA, other allele; SE, standard error; PTSD, post-traumatic stress disorder.

**Supplementary Table S1.3** Information of identified SNPs in exposure (PTSD) and outcomes (Hypertension).

|  |  | | | **Exposure (PTSD)** | | |  | **Outcome (Hypertension)** | | | | |
| --- | --- | --- | --- | --- | --- | --- | --- | --- | --- | --- | --- | --- |
|  | **SNP** | **EA** | **OA** | **β** | **SE** | ***p* value** |  | **Case** | **Control** | **β** | **SE** | ***p* value** |
| 1 | rs1024510 | G | A | -0.219903 | 0.0538123 | 4.38E-05 |  | 119,731 | 343,202 | -0.00240912 | 0.00138085 | 0.0810009 |
| 2 | rs1032419 | C | T | -0.145257 | 0.0355232 | 4.33E-05 |  | 119,731 | 343,202 | -0.00243002 | 0.00117322 | 0.0379997 |
| 3 | rs10512159 | A | G | 0.159327 | 0.0366852 | 1.40E-05 |  | 119,731 | 343,202 | 0.000485351 | 0.00128258 | 0.709999 |
| 4 | rs10857816 | G | A | 0.109934 | 0.0261106 | 2.55E-05 |  | 119,731 | 343,202 | 0.0011052 | 0.000916383 | 0.23 |
| 5 | rs10938438 | C | T | 0.129063 | 0.0294969 | 1.21E-05 |  | 119,731 | 343,202 | -0.000959026 | 0.000946738 | 0.31 |
| 6 | rs10982385 | G | T | 0.11 | 0.0264619 | 3.23E-05 |  | 119,731 | 343,202 | -0.00131969 | 0.000914895 | 0.15 |
| 7 | rs11244418 | C | T | -0.225322 | 0.0515111 | 1.22E-05 |  | 119,731 | 343,202 | -8.15E-05 | 0.00218352 | 0.97 |
| 8 | rs112463264 | T | G | 0.303551 | 0.0734204 | 3.56E-05 |  | 119,731 | 343,202 | -0.00152381 | 0.00267934 | 0.57 |
| 9 | rs112685439 | C | T | -0.304907 | 0.062345 | 1.01E-06 |  | 119,731 | 343,202 | -0.00214394 | 0.00192252 | 0.26 |
| 10 | rs113457140 | C | G | 0.283916 | 0.0666363 | 2.04E-05 |  | 119,731 | 343,202 | -0.00332146 | 0.00612815 | 0.59 |
| 11 | rs113968366 | T | C | -0.513073 | 0.125182 | 4.16E-05 |  | 119,731 | 343,202 | -0.00301005 | 0.0039829 | 0.450001 |
| 12 | rs113986839 | T | G | 0.265891 | 0.0603691 | 1.06E-05 |  | 119,731 | 343,202 | -5.24E-05 | 0.00297782 | 0.99 |
| 13 | rs114850138 | G | A | 0.429768 | 0.0922631 | 3.19E-06 |  | 119,731 | 343,202 | 0.00252119 | 0.00306363 | 0.41 |
| 14 | rs115567710 | A | G | 0.418466 | 0.101522 | 3.76E-05 |  | 119,731 | 343,202 | 0.00043325 | 0.00497286 | 0.93 |
| 15 | rs1160219 | C | T | 0.142632 | 0.0331717 | 1.71E-05 |  | 119,731 | 343,202 | 0.000247631 | 0.00109047 | 0.82 |
| 16 | rs116174051 | C | T | 0.535461 | 0.119247 | 7.11E-06 |  | 119,731 | 343,202 | 0.0029467 | 0.00346422 | 0.39 |
| 17 | rs11719094 | T | G | 0.159073 | 0.0369491 | 1.67E-05 |  | 119,731 | 343,202 | -0.000377414 | 0.00125759 | 0.760001 |
| 18 | rs117367614 | T | C | 0.36126 | 0.0838906 | 1.66E-05 |  | 119,731 | 343,202 | 0.000418324 | 0.00271761 | 0.88 |
| 19 | rs11764137 | T | C | -0.242849 | 0.0576301 | 2.51E-05 |  | 119,731 | 343,202 | 0.000110993 | 0.00220672 | 0.96 |
| 20 | rs11864961 | A | G | -0.183884 | 0.0351212 | 1.64E-07 |  | 119,731 | 343,202 | 0.00128552 | 0.0013232 | 0.33 |
| 21 | rs12042286 | A | T | 0.190387 | 0.0428416 | 8.83E-06 |  | 119,731 | 343,202 | 0.000581197 | 0.00167753 | 0.73 |
| 22 | rs1273044 | C | T | -0.109143 | 0.0263228 | 3.38E-05 |  | 119,731 | 343,202 | 0.00126377 | 0.00111757 | 0.26 |
| 23 | rs12731117 | T | C | -0.147557 | 0.0342711 | 1.67E-05 |  | 119,731 | 343,202 | -0.000798236 | 0.00104429 | 0.44 |
| 24 | rs12764494 | A | G | -0.54991 | 0.134203 | 4.17E-05 |  | 119,731 | 343,202 | -0.00451761 | 0.00240563 | 0.0599998 |
| 25 | rs12929184 | A | G | -0.160228 | 0.0389966 | 3.98E-05 |  | 119,731 | 343,202 | -0.00116447 | 0.00140728 | 0.41 |
| 26 | rs13094101 | A | C | -0.182339 | 0.0379614 | 1.56E-06 |  | 119,731 | 343,202 | -0.000805159 | 0.00118606 | 0.5 |
| 27 | rs139670285 | T | C | 0.265025 | 0.0645787 | 4.06E-05 |  | 119,731 | 343,202 | -0.00488328 | 0.00213597 | 0.0219999 |
| 28 | rs147059451 | T | G | 0.264574 | 0.0641234 | 3.69E-05 |  | 119,731 | 343,202 | 0.00210756 | 0.00303295 | 0.49 |
| 29 | rs147438061 | A | G | -0.147717 | 0.0336811 | 1.16E-05 |  | 119,731 | 343,202 | 0.000130332 | 0.00122357 | 0.92 |
| 30 | rs147512888 | G | A | -0.460906 | 0.108819 | 2.28E-05 |  | 119,731 | 343,202 | -0.00373348 | 0.00375846 | 0.32 |
| 31 | rs147761221 | A | G | -0.412908 | 0.0875558 | 2.41E-06 |  | 119,731 | 343,202 | 0.00116825 | 0.00507903 | 0.82 |
| 32 | rs147764711 | G | A | 0.284803 | 0.0664584 | 1.82E-05 |  | 119,731 | 343,202 | -0.000193654 | 0.0034353 | 0.96 |
| 33 | rs148253594 | C | T | 0.512693 | 0.123652 | 3.38E-05 |  | 119,731 | 343,202 | -0.00360996 | 0.00409446 | 0.38 |
| 34 | rs1506001 | A | G | -0.169846 | 0.0384237 | 9.85E-06 |  | 119,731 | 343,202 | 0.000212944 | 0.00115418 | 0.85 |
| 35 | rs17053171 | T | C | 0.289219 | 0.0688782 | 2.68E-05 |  | 119,731 | 343,202 | 0.0031439 | 0.00199627 | 0.12 |
| 36 | rs17225380 | A | G | 0.13851 | 0.032863 | 2.50E-05 |  | 119,731 | 343,202 | 0.000784792 | 0.00112459 | 0.49 |
| 37 | rs17368930 | A | T | 0.419127 | 0.101686 | 3.76E-05 |  | 119,731 | 343,202 | -0.00394042 | 0.0032568 | 0.23 |
| 38 | rs1845130 | T | G | -0.110026 | 0.0266575 | 3.67E-05 |  | 119,731 | 343,202 | 0.000419231 | 0.000958653 | 0.66 |
| 39 | rs192336096 | A | G | -1.22335 | 0.289787 | 2.43E-05 |  | 119,731 | 343,202 | -0.00596903 | 0.00357825 | 0.0949992 |
| 40 | rs1975254 | C | T | 0.132761 | 0.0312117 | 2.10E-05 |  | 119,731 | 343,202 | 0.00141434 | 0.00119015 | 0.23 |
| 41 | rs2249245 | A | G | -0.155072 | 0.0312091 | 6.74E-07 |  | 119,731 | 343,202 | 0.00163368 | 0.00107808 | 0.13 |
| 42 | rs2554818 | A | G | 0.143588 | 0.0328117 | 1.21E-05 |  | 119,731 | 343,202 | 0.00143019 | 0.00103062 | 0.17 |
| 43 | rs35277443 | A | C | -0.169386 | 0.0348745 | 1.19E-06 |  | 119,731 | 343,202 | -0.00236726 | 0.00109064 | 0.0299999 |
| 44 | rs36076286 | G | A | 0.118192 | 0.0285824 | 3.55E-05 |  | 119,731 | 343,202 | -0.000640248 | 0.000956915 | 0.5 |
| 45 | rs3741475 | A | G | 0.139951 | 0.0341593 | 4.19E-05 |  | 119,731 | 343,202 | 0.00174146 | 0.00112756 | 0.12 |
| 46 | rs3741578 | C | G | -0.297837 | 0.0729471 | 4.45E-05 |  | 119,731 | 343,202 | -0.00103633 | 0.00159089 | 0.51 |
| 47 | rs3956254 | T | C | -0.154452 | 0.0349614 | 9.97E-06 |  | 119,731 | 343,202 | 8.73E-05 | 0.00126307 | 0.94 |
| 48 | rs4410329 | A | G | -0.123388 | 0.0284311 | 1.43E-05 |  | 119,731 | 343,202 | 0.000584007 | 0.00108333 | 0.59 |
| 49 | rs4548693 | C | A | -0.123961 | 0.0296464 | 2.90E-05 |  | 119,731 | 343,202 | 0.000865697 | 0.000993822 | 0.38 |
| 50 | rs4558785 | T | A | -0.127557 | 0.0299171 | 2.01E-05 |  | 119,731 | 343,202 | -0.00125396 | 0.00102321 | 0.22 |
| 51 | rs4683357 | G | A | -0.125441 | 0.0270408 | 3.50E-06 |  | 119,731 | 343,202 | -0.000694534 | 0.000905456 | 0.44 |
| 52 | rs4787028 | C | T | 0.114579 | 0.0275383 | 3.17E-05 |  | 119,731 | 343,202 | 0.00152657 | 0.000948785 | 0.11 |
| 53 | rs56078972 | G | T | -0.111841 | 0.0262733 | 2.07E-05 |  | 119,731 | 343,202 | 0.00125643 | 0.000914818 | 0.17 |
| 54 | rs587351 | C | A | -0.112457 | 0.0262745 | 1.87E-05 |  | 119,731 | 343,202 | 0.00055361 | 0.000898004 | 0.54 |
| 55 | rs61748881 | G | T | -0.31663 | 0.0564936 | 2.09E-08 |  | 119,731 | 343,202 | -0.00160354 | 0.00137889 | 0.24 |
| 56 | rs62096966 | G | T | 0.16496 | 0.0396832 | 3.23E-05 |  | 119,731 | 343,202 | 0.00169147 | 0.00114803 | 0.14 |
| 57 | rs643089 | G | T | 0.229217 | 0.055854 | 4.06E-05 |  | 119,731 | 343,202 | 0.00154794 | 0.00152095 | 0.31 |
| 58 | rs678246 | G | T | -0.191238 | 0.0451704 | 2.30E-05 |  | 119,731 | 343,202 | -0.00134541 | 0.00148809 | 0.37 |
| 59 | rs7080218 | A | G | -0.120927 | 0.0297889 | 4.92E-05 |  | 119,731 | 343,202 | -0.000225013 | 0.00100215 | 0.82 |
| 60 | rs71385313 | G | T | 0.276722 | 0.0677608 | 4.43E-05 |  | 119,731 | 343,202 | -0.00309135 | 0.00232738 | 0.18 |
| 61 | rs72637580 | C | T | 0.277385 | 0.0599411 | 3.70E-06 |  | 119,731 | 343,202 | 0.00169881 | 0.00163221 | 0.3 |
| 62 | rs72806097 | T | C | 0.484445 | 0.115027 | 2.54E-05 |  | 119,731 | 343,202 | 0.00369301 | 0.00408412 | 0.37 |
| 63 | rs72827609 | A | G | -0.461125 | 0.111496 | 3.54E-05 |  | 119,731 | 343,202 | -0.000266511 | 0.00231258 | 0.91 |
| 64 | rs73057373 | T | C | 0.193197 | 0.0471536 | 4.18E-05 |  | 119,731 | 343,202 | 0.00414515 | 0.00167247 | 0.0129999 |
| 65 | rs74516805 | A | G | 0.369337 | 0.0885093 | 3.01E-05 |  | 119,731 | 343,202 | 0.00136809 | 0.00311049 | 0.66 |
| 66 | rs74812538 | C | T | 0.390509 | 0.0918243 | 2.11E-05 |  | 119,731 | 343,202 | -0.00117984 | 0.00306596 | 0.7 |
| 67 | rs74863973 | C | T | -0.144354 | 0.0346156 | 3.04E-05 |  | 119,731 | 343,202 | 0.00254693 | 0.00146899 | 0.0830004 |
| 68 | rs75598025 | G | A | 0.520541 | 0.119165 | 1.25E-05 |  | 119,731 | 343,202 | -0.00701338 | 0.00463567 | 0.13 |
| 69 | rs7562028 | A | G | -0.360709 | 0.082574 | 1.25E-05 |  | 119,731 | 343,202 | 0.000432671 | 0.0016997 | 0.8 |
| 70 | rs75641198 | T | C | 0.274826 | 0.0575213 | 1.77E-06 |  | 119,731 | 343,202 | 0.00176691 | 0.00170307 | 0.3 |
| 71 | rs75928930 | C | T | 0.281701 | 0.0672459 | 2.80E-05 |  | 119,731 | 343,202 | -0.00183868 | 0.00281904 | 0.51 |
| 72 | rs76355089 | G | A | 0.249634 | 0.0602865 | 3.46E-05 |  | 119,731 | 343,202 | 0.00281948 | 0.00221731 | 0.2 |
| 73 | rs76404313 | G | A | 1.27887 | 0.314601 | 4.80E-05 |  | 119,731 | 343,202 | -0.00468207 | 0.00448568 | 0.3 |
| 74 | rs769018 | T | C | 0.190415 | 0.0459608 | 3.43E-05 |  | 119,731 | 343,202 | -0.00134877 | 0.00131877 | 0.31 |
| 75 | rs78588594 | C | G | 0.209619 | 0.0486867 | 1.67E-05 |  | 119,731 | 343,202 | -0.000648766 | 0.00220144 | 0.77 |
| 76 | rs78791514 | T | C | -0.473445 | 0.115499 | 4.15E-05 |  | 119,731 | 343,202 | -0.00391558 | 0.00238489 | 0.1 |
| 77 | rs79142281 | A | C | -0.467126 | 0.110791 | 2.48E-05 |  | 119,731 | 343,202 | 0.00254596 | 0.00272349 | 0.35 |
| 78 | rs79433303 | T | G | 0.660108 | 0.147299 | 7.41E-06 |  | 119,731 | 343,202 | 0.00153244 | 0.00324849 | 0.64 |
| 79 | rs79968379 | G | A | -0.467312 | 0.110772 | 2.46E-05 |  | 119,731 | 343,202 | 0.000667446 | 0.00198536 | 0.74 |
| 80 | rs8003680 | T | C | 0.121925 | 0.0277197 | 1.09E-05 |  | 119,731 | 343,202 | -0.000188713 | 0.000905512 | 0.83 |
| 81 | rs80038448 | T | C | 0.18611 | 0.0403734 | 4.03E-06 |  | 119,731 | 343,202 | 0.000104092 | 0.00126489 | 0.93 |
| 82 | rs80261117 | A | G | 0.3007 | 0.0671 | 7.42E-06 |  | 119,731 | 343,202 | -0.00155796 | 0.00202577 | 0.44 |
| 83 | rs868209 | A | T | -0.168971 | 0.0395824 | 1.96E-05 |  | 119,731 | 343,202 | -0.00091874 | 0.00112924 | 0.42 |
| 84 | rs9453417 | A | G | 0.458284 | 0.106145 | 1.58E-05 |  | 119,731 | 343,202 | -0.00306045 | 0.00379704 | 0.42 |
| 85 | rs9526717 | G | A | 0.125284 | 0.0270735 | 3.70E-06 |  | 119,731 | 343,202 | -0.00194071 | 0.000923277 | 0.0359998 |
| 86 | rs9809585 | G | A | -0.108248 | 0.026432 | 4.22E-05 |  | 119,731 | 343,202 | -0.00149848 | 0.000904838 | 0.0980009 |
| 87 | rs9900367 | C | T | -0.131089 | 0.0285907 | 4.54E-06 |  | 119,731 | 343,202 | -0.00104169 | 0.000968312 | 0.28 |
| 88 | rs9968731 | T | C | -0.124696 | 0.0299353 | 3.11E-05 |  | 119,731 | 343,202 | -0.00132559 | 0.000951095 | 0.16 |
| 89 | rs9971308 | G | A | 0.136043 | 0.0323547 | 2.61E-05 |  | 119,731 | 343,202 | 0.000495142 | 0.00110913 | 0.66 |

SNP, single nucleotide polymorphism; EA, effect allele; OA, other allele; SE, standard error; PTSD, post-traumatic stress disorder.

**Supplementary Table S1.4** Information of identified SNPs in exposure (PTSD) and outcomes (Hyperlipidemia).

|  |  | | | **Exposure (PTSD)** | | |  | **Outcome (Hyperlipidemia)** | | | | |
| --- | --- | --- | --- | --- | --- | --- | --- | --- | --- | --- | --- | --- |
|  | **SNP** | **EA** | **OA** | **β** | **SE** | ***p* value** |  | **Case** | **Control** | **β** | **SE** | ***p* value** |
| 1 | rs1024510 | G | A | -0.219903 | 0.0538123 | 4.38E-05 |  | 39,961 | 309,261 | 0.0194766 | 0.0393412 | 0.62055 |
| 2 | rs1032419 | C | T | -0.145257 | 0.0355232 | 4.33E-05 |  | 39,961 | 309,261 | -0.0287729 | 0.014644 | 0.0494345 |
| 3 | rs10512159 | A | G | 0.159327 | 0.0366852 | 1.40E-05 |  | 39,961 | 309,261 | -0.00802794 | 0.0145399 | 0.580858 |
| 4 | rs10857816 | G | A | 0.109934 | 0.0261106 | 2.55E-05 |  | 39,961 | 309,261 | 0.0301221 | 0.0238857 | 0.207274 |
| 5 | rs10938438 | C | T | 0.129063 | 0.0294969 | 1.21E-05 |  | 39,961 | 309,261 | -0.0258985 | 0.0189839 | 0.172492 |
| 6 | rs10982385 | G | T | 0.11 | 0.0264619 | 3.23E-05 |  | 39,961 | 309,261 | -0.0136178 | 0.0380305 | 0.720286 |
| 7 | rs11151641 | A | G | 0.115256 | 0.0263531 | 1.22E-05 |  | 39,961 | 309,261 | -0.00491752 | 0.0113638 | 0.665206 |
| 8 | rs112256243 | C | G | 3.75787 | 0.923226 | 4.69E-05 |  | 39,961 | 309,261 | 0.00435788 | 0.0188669 | 0.81733 |
| 9 | rs11244418 | C | T | -0.225322 | 0.0515111 | 1.22E-05 |  | 39,961 | 309,261 | -0.00504 | 0.0203151 | 0.804064 |
| 10 | rs112463264 | T | G | 0.303551 | 0.0734204 | 3.56E-05 |  | 39,961 | 309,261 | 0.00395814 | 0.023477 | 0.866114 |
| 11 | rs112685439 | C | T | -0.304907 | 0.062345 | 1.01E-06 |  | 39,961 | 309,261 | 0.0110428 | 0.0275849 | 0.68892 |
| 12 | rs113457140 | C | G | 0.283916 | 0.0666363 | 2.04E-05 |  | 39,961 | 309,261 | 0.00631945 | 0.0169396 | 0.709104 |
| 13 | rs113968366 | T | C | -0.513073 | 0.125182 | 4.16E-05 |  | 39,961 | 309,261 | 0.0540758 | 0.0235501 | 0.0216641 |
| 14 | rs113986839 | T | G | 0.265891 | 0.0603691 | 1.06E-05 |  | 39,961 | 309,261 | -0.00255181 | 0.00776458 | 0.742422 |
| 15 | rs114850138 | G | A | 0.429768 | 0.0922631 | 3.19E-06 |  | 39,961 | 309,261 | 0.0115246 | 0.0108545 | 0.28836 |
| 16 | rs115567710 | A | G | 0.418466 | 0.101522 | 3.76E-05 |  | 39,961 | 309,261 | 0.0167211 | 0.0175395 | 0.340418 |
| 17 | rs1160219 | C | T | 0.142632 | 0.0331717 | 1.71E-05 |  | 39,961 | 309,261 | 0.0105992 | 0.00968388 | 0.273727 |
| 18 | rs116174051 | C | T | 0.535461 | 0.119247 | 7.11E-06 |  | 39,961 | 309,261 | 0.0391241 | 0.0330732 | 0.236827 |
| 19 | rs11719094 | T | G | 0.159073 | 0.0369491 | 1.67E-05 |  | 39,961 | 309,261 | -0.00906635 | 0.00790762 | 0.251575 |
| 20 | rs117367614 | T | C | 0.36126 | 0.0838906 | 1.66E-05 |  | 39,961 | 309,261 | -0.00386629 | 0.00775999 | 0.61832 |
| 21 | rs11764137 | T | C | -0.242849 | 0.0576301 | 2.51E-05 |  | 39,961 | 309,261 | -0.0104579 | 0.00828571 | 0.206893 |
| 22 | rs11864961 | A | G | -0.183884 | 0.0351212 | 1.64E-07 |  | 39,961 | 309,261 | -0.00784671 | 0.00816025 | 0.336262 |
| 23 | rs12042286 | A | T | 0.190387 | 0.0428416 | 8.83E-06 |  | 39,961 | 309,261 | -0.0198136 | 0.00950963 | 0.0372032 |
| 24 | rs1273044 | C | T | -0.109143 | 0.0263228 | 3.38E-05 |  | 39,961 | 309,261 | 0.0194766 | 0.0393412 | 0.62055 |
| 25 | rs12731117 | T | C | -0.147557 | 0.0342711 | 1.67E-05 |  | 39,961 | 309,261 | -0.0287729 | 0.014644 | 0.0494345 |
| 26 | rs12764494 | A | G | -0.54991 | 0.134203 | 4.17E-05 |  | 39,961 | 309,261 | -0.00802794 | 0.0145399 | 0.580858 |
| 27 | rs12929184 | A | G | -0.160228 | 0.0389966 | 3.98E-05 |  | 39,961 | 309,261 | 0.0301221 | 0.0238857 | 0.207274 |
| 28 | rs13094101 | A | C | -0.182339 | 0.0379614 | 1.56E-06 |  | 39,961 | 309,261 | -0.0258985 | 0.0189839 | 0.172492 |
| 29 | rs1362910 | G | A | -0.11021 | 0.0269297 | 4.27E-05 |  | 39,961 | 309,261 | -0.0136178 | 0.0380305 | 0.720286 |
| 30 | rs139670285 | T | C | 0.265025 | 0.0645787 | 4.06E-05 |  | 39,961 | 309,261 | -0.00491752 | 0.0113638 | 0.665206 |
| 31 | rs144814409 | A | G | -0.935522 | 0.21933 | 2.00E-05 |  | 39,961 | 309,261 | 0.00435788 | 0.0188669 | 0.81733 |
| 32 | rs145209482 | A | G | 1.50508 | 0.368393 | 4.40E-05 |  | 39,961 | 309,261 | -0.00504 | 0.0203151 | 0.804064 |
| 33 | rs147059451 | T | G | 0.264574 | 0.0641234 | 3.69E-05 |  | 39,961 | 309,261 | 0.00395814 | 0.023477 | 0.866114 |
| 34 | rs147438061 | A | G | -0.147717 | 0.0336811 | 1.16E-05 |  | 39,961 | 309,261 | 0.0110428 | 0.0275849 | 0.68892 |
| 35 | rs147512888 | G | A | -0.460906 | 0.108819 | 2.28E-05 |  | 39,961 | 309,261 | 0.00631945 | 0.0169396 | 0.709104 |
| 36 | rs147761221 | A | G | -0.412908 | 0.0875558 | 2.41E-06 |  | 39,961 | 309,261 | 0.0540758 | 0.0235501 | 0.0216641 |
| 37 | rs147764711 | G | A | 0.284803 | 0.0664584 | 1.82E-05 |  | 39,961 | 309,261 | -0.00255181 | 0.00776458 | 0.742422 |
| 38 | rs148253594 | C | T | 0.512693 | 0.123652 | 3.38E-05 |  | 39,961 | 309,261 | 0.0115246 | 0.0108545 | 0.28836 |
| 39 | rs1506001 | A | G | -0.169846 | 0.0384237 | 9.85E-06 |  | 39,961 | 309,261 | 0.0167211 | 0.0175395 | 0.340418 |
| 40 | rs17053171 | T | C | 0.289219 | 0.0688782 | 2.68E-05 |  | 39,961 | 309,261 | 0.0105992 | 0.00968388 | 0.273727 |
| 41 | rs17225380 | A | G | 0.13851 | 0.032863 | 2.50E-05 |  | 39,961 | 309,261 | 0.0391241 | 0.0330732 | 0.236827 |
| 42 | rs17368930 | A | T | 0.419127 | 0.101686 | 3.76E-05 |  | 39,961 | 309,261 | -0.00906635 | 0.00790762 | 0.251575 |
| 43 | rs1845130 | T | G | -0.110026 | 0.0266575 | 3.67E-05 |  | 39,961 | 309,261 | -0.00386629 | 0.00775999 | 0.61832 |
| 44 | rs192336096 | A | G | -1.22335 | 0.289787 | 2.43E-05 |  | 39,961 | 309,261 | -0.0104579 | 0.00828571 | 0.206893 |
| 45 | rs1975254 | C | T | 0.132761 | 0.0312117 | 2.10E-05 |  | 39,961 | 309,261 | -0.00784671 | 0.00816025 | 0.336262 |
| 46 | rs2249245 | A | G | -0.155072 | 0.0312091 | 6.74E-07 |  | 39,961 | 309,261 | -0.0198136 | 0.00950963 | 0.0372032 |
| 47 | rs2554818 | A | G | 0.143588 | 0.0328117 | 1.21E-05 |  | 39,961 | 309,261 | 0.0194766 | 0.0393412 | 0.62055 |
| 48 | rs35277443 | A | C | -0.169386 | 0.0348745 | 1.19E-06 |  | 39,961 | 309,261 | -0.0287729 | 0.014644 | 0.0494345 |
| 49 | rs36076286 | G | A | 0.118192 | 0.0285824 | 3.55E-05 |  | 39,961 | 309,261 | -0.00802794 | 0.0145399 | 0.580858 |
| 50 | rs3741475 | A | G | 0.139951 | 0.0341593 | 4.19E-05 |  | 39,961 | 309,261 | 0.0301221 | 0.0238857 | 0.207274 |
| 51 | rs3741578 | C | G | -0.297837 | 0.0729471 | 4.45E-05 |  | 39,961 | 309,261 | -0.0258985 | 0.0189839 | 0.172492 |
| 52 | rs3956254 | T | C | -0.154452 | 0.0349614 | 9.97E-06 |  | 39,961 | 309,261 | -0.0136178 | 0.0380305 | 0.720286 |
| 53 | rs4410329 | A | G | -0.123388 | 0.0284311 | 1.43E-05 |  | 39,961 | 309,261 | -0.00491752 | 0.0113638 | 0.665206 |
| 54 | rs4536683 | A | G | 0.153157 | 0.0350246 | 1.23E-05 |  | 39,961 | 309,261 | 0.00435788 | 0.0188669 | 0.81733 |
| 55 | rs4548693 | C | A | -0.123961 | 0.0296464 | 2.90E-05 |  | 39,961 | 309,261 | -0.00504 | 0.0203151 | 0.804064 |
| 56 | rs4558785 | T | A | -0.127557 | 0.0299171 | 2.01E-05 |  | 39,961 | 309,261 | 0.00395814 | 0.023477 | 0.866114 |
| 57 | rs4683357 | G | A | -0.125441 | 0.0270408 | 3.50E-06 |  | 39,961 | 309,261 | 0.0110428 | 0.0275849 | 0.68892 |
| 58 | rs4787028 | C | T | 0.114579 | 0.0275383 | 3.17E-05 |  | 39,961 | 309,261 | 0.00631945 | 0.0169396 | 0.709104 |
| 59 | rs559398226 | C | T | 0.565271 | 0.130732 | 1.53E-05 |  | 39,961 | 309,261 | 0.0540758 | 0.0235501 | 0.0216641 |
| 60 | rs56078972 | G | T | -0.111841 | 0.0262733 | 2.07E-05 |  | 39,961 | 309,261 | -0.00255181 | 0.00776458 | 0.742422 |
| 61 | rs587351 | C | A | -0.112457 | 0.0262745 | 1.87E-05 |  | 39,961 | 309,261 | 0.0115246 | 0.0108545 | 0.28836 |
| 62 | rs61748881 | G | T | -0.31663 | 0.0564936 | 2.09E-08 |  | 39,961 | 309,261 | 0.0167211 | 0.0175395 | 0.340418 |
| 63 | rs62096966 | G | T | 0.16496 | 0.0396832 | 3.23E-05 |  | 39,961 | 309,261 | 0.0105992 | 0.00968388 | 0.273727 |
| 64 | rs643089 | G | T | 0.229217 | 0.055854 | 4.06E-05 |  | 39,961 | 309,261 | 0.0391241 | 0.0330732 | 0.236827 |
| 65 | rs678246 | G | T | -0.191238 | 0.0451704 | 2.30E-05 |  | 39,961 | 309,261 | -0.00906635 | 0.00790762 | 0.251575 |
| 66 | rs7080218 | A | G | -0.120927 | 0.0297889 | 4.92E-05 |  | 39,961 | 309,261 | -0.00386629 | 0.00775999 | 0.61832 |
| 67 | rs71385313 | G | T | 0.276722 | 0.0677608 | 4.43E-05 |  | 39,961 | 309,261 | -0.0104579 | 0.00828571 | 0.206893 |
| 68 | rs72637580 | C | T | 0.277385 | 0.0599411 | 3.70E-06 |  | 39,961 | 309,261 | -0.00784671 | 0.00816025 | 0.336262 |
| 69 | rs72827609 | A | G | -0.461125 | 0.111496 | 3.54E-05 |  | 39,961 | 309,261 | -0.0198136 | 0.00950963 | 0.0372032 |
| 70 | rs73057373 | T | C | 0.193197 | 0.0471536 | 4.18E-05 |  | 39,961 | 309,261 | 0.0194766 | 0.0393412 | 0.62055 |
| 71 | rs73200307 | T | C | -0.158719 | 0.0382272 | 3.30E-05 |  | 39,961 | 309,261 | -0.0287729 | 0.014644 | 0.0494345 |
| 72 | rs74516805 | A | G | 0.369337 | 0.0885093 | 3.01E-05 |  | 39,961 | 309,261 | -0.00802794 | 0.0145399 | 0.580858 |
| 73 | rs74812538 | C | T | 0.390509 | 0.0918243 | 2.11E-05 |  | 39,961 | 309,261 | 0.0301221 | 0.0238857 | 0.207274 |
| 74 | rs74863973 | C | T | -0.144354 | 0.0346156 | 3.04E-05 |  | 39,961 | 309,261 | -0.0258985 | 0.0189839 | 0.172492 |
| 75 | rs75598025 | G | A | 0.520541 | 0.119165 | 1.25E-05 |  | 39,961 | 309,261 | -0.0136178 | 0.0380305 | 0.720286 |
| 76 | rs7562028 | A | G | -0.360709 | 0.082574 | 1.25E-05 |  | 39,961 | 309,261 | -0.00491752 | 0.0113638 | 0.665206 |
| 77 | rs75641198 | T | C | 0.274826 | 0.0575213 | 1.77E-06 |  | 39,961 | 309,261 | 0.00435788 | 0.0188669 | 0.81733 |
| 78 | rs75928930 | C | T | 0.281701 | 0.0672459 | 2.80E-05 |  | 39,961 | 309,261 | -0.00504 | 0.0203151 | 0.804064 |
| 79 | rs76355089 | G | A | 0.249634 | 0.0602865 | 3.46E-05 |  | 39,961 | 309,261 | 0.00395814 | 0.023477 | 0.866114 |
| 80 | rs76404313 | G | A | 1.27887 | 0.314601 | 4.80E-05 |  | 39,961 | 309,261 | 0.0110428 | 0.0275849 | 0.68892 |
| 81 | rs769018 | T | C | 0.190415 | 0.0459608 | 3.43E-05 |  | 39,961 | 309,261 | 0.00631945 | 0.0169396 | 0.709104 |
| 82 | rs78588594 | C | G | 0.209619 | 0.0486867 | 1.67E-05 |  | 39,961 | 309,261 | 0.0540758 | 0.0235501 | 0.0216641 |
| 83 | rs78791514 | T | C | -0.473445 | 0.115499 | 4.15E-05 |  | 39,961 | 309,261 | -0.00255181 | 0.00776458 | 0.742422 |
| 84 | rs79142281 | A | C | -0.467126 | 0.110791 | 2.48E-05 |  | 39,961 | 309,261 | 0.0115246 | 0.0108545 | 0.28836 |
| 85 | rs79433303 | T | G | 0.660108 | 0.147299 | 7.41E-06 |  | 39,961 | 309,261 | 0.0167211 | 0.0175395 | 0.340418 |
| 86 | rs79968379 | G | A | -0.467312 | 0.110772 | 2.46E-05 |  | 39,961 | 309,261 | 0.0105992 | 0.00968388 | 0.273727 |
| 87 | rs80021344 | G | A | 0.718864 | 0.168948 | 2.09E-05 |  | 39,961 | 309,261 | 0.0391241 | 0.0330732 | 0.236827 |
| 88 | rs8003680 | T | C | 0.121925 | 0.0277197 | 1.09E-05 |  | 39,961 | 309,261 | -0.00906635 | 0.00790762 | 0.251575 |
| 89 | rs80038448 | T | C | 0.18611 | 0.0403734 | 4.03E-06 |  | 39,961 | 309,261 | -0.00386629 | 0.00775999 | 0.61832 |
| 90 | rs80261117 | A | G | 0.3007 | 0.0671 | 7.42E-06 |  | 39,961 | 309,261 | -0.0104579 | 0.00828571 | 0.206893 |
| 91 | rs868209 | A | T | -0.168971 | 0.0395824 | 1.96E-05 |  | 39,961 | 309,261 | -0.00784671 | 0.00816025 | 0.336262 |
| 92 | rs9453417 | A | G | 0.458284 | 0.106145 | 1.58E-05 |  | 39,961 | 309,261 | -0.0198136 | 0.00950963 | 0.0372032 |
| 93 | rs9526717 | G | A | 0.125284 | 0.0270735 | 3.70E-06 |  | 39,961 | 309,261 | 0.0194766 | 0.0393412 | 0.62055 |
| 94 | rs9809585 | G | A | -0.108248 | 0.026432 | 4.22E-05 |  | 39,961 | 309,261 | -0.0287729 | 0.014644 | 0.0494345 |
| 95 | rs9900367 | C | T | -0.131089 | 0.0285907 | 4.54E-06 |  | 39,961 | 309,261 | -0.00802794 | 0.0145399 | 0.580858 |
| 96 | rs9968731 | T | C | -0.124696 | 0.0299353 | 3.11E-05 |  | 39,961 | 309,261 | 0.0301221 | 0.0238857 | 0.207274 |
| 97 | rs9971308 | G | A | 0.136043 | 0.0323547 | 2.61E-05 |  | 39,961 | 309,261 | -0.0258985 | 0.0189839 | 0.172492 |

SNP, single nucleotide polymorphism; EA, effect allele; OA, other allele; SE, standard error; PTSD, post-traumatic stress disorder.

**Supplementary Table S1.5** Information of identified SNPs in exposure (PTSD) and outcomes (Alcohol consumption).

|  |  | | | **Exposure (PTSD)** | | |  | **Outcome (Alcohol consumption)** | | | |
| --- | --- | --- | --- | --- | --- | --- | --- | --- | --- | --- | --- |
|  | **SNP** | **EA** | **OA** | **β** | **SE** | ***p* value** |  | **Samplesize** | **β** | **SE** | ***p* value** |
| 1 | rs1024510 | G | A | -0.219903 | 0.0538123 | 4.38E-05 |  | 83,626 | 0.0336 | 0.0267 | 0.2075 |
| 2 | rs1032419 | C | T | -0.145257 | 0.0355232 | 4.33E-05 |  | 83,626 | -0.0156 | 0.021 | 0.4579 |
| 3 | rs10512159 | A | G | 0.159327 | 0.0366852 | 1.40E-05 |  | 83,626 | -0.0518 | 0.0229 | 0.0237099 |
| 4 | rs10857816 | G | A | 0.109934 | 0.0261106 | 2.55E-05 |  | 83,626 | 0.0022 | 0.0178 | 0.9008 |
| 5 | rs10938438 | C | T | 0.129063 | 0.0294969 | 1.21E-05 |  | 83,626 | 0.0277 | 0.0189 | 0.1416 |
| 6 | rs10982385 | G | T | 0.11 | 0.0264619 | 3.23E-05 |  | 83,626 | 0.0142 | 0.0176 | 0.4207 |
| 7 | rs11151641 | A | G | 0.115256 | 0.0263531 | 1.22E-05 |  | 83,626 | -0.0283 | 0.0192 | 0.1404 |
| 8 | rs112256243 | C | G | 3.75787 | 0.923226 | 4.69E-05 |  | 83,626 | -0.0067 | 0.0359 | 0.8521 |
| 9 | rs11244418 | C | T | -0.225322 | 0.0515111 | 1.22E-05 |  | 83,626 | 0.0094 | 0.0515 | 0.8557 |
| 10 | rs112463264 | T | G | 0.303551 | 0.0734204 | 3.56E-05 |  | 83,626 | -0.069 | 0.0348 | 0.0472498 |
| 11 | rs112685439 | C | T | -0.304907 | 0.062345 | 1.01E-06 |  | 83,626 | -0.0127 | 0.0724 | 0.861 |
| 12 | rs113457140 | C | G | 0.283916 | 0.0666363 | 2.04E-05 |  | 83,626 | -0.0025 | 0.057 | 0.9647 |
| 13 | rs113968366 | T | C | -0.513073 | 0.125182 | 4.16E-05 |  | 83,626 | -0.009 | 0.0216 | 0.6754 |
| 14 | rs113986839 | T | G | 0.265891 | 0.0603691 | 1.06E-05 |  | 83,626 | 0.1015 | 0.0678 | 0.1345 |
| 15 | rs114850138 | G | A | 0.429768 | 0.0922631 | 3.19E-06 |  | 83,626 | -0.0322 | 0.0246 | 0.1901 |
| 16 | rs115567710 | A | G | 0.418466 | 0.101522 | 3.76E-05 |  | 83,626 | 0.0146 | 0.0395 | 0.7117 |
| 17 | rs1160219 | C | T | 0.142632 | 0.0331717 | 1.71E-05 |  | 83,626 | -0.0147 | 0.0255 | 0.5634 |
| 18 | rs116174051 | C | T | 0.535461 | 0.119247 | 7.11E-06 |  | 83,626 | 0.0127 | 0.0201 | 0.5281 |
| 19 | rs11719094 | T | G | 0.159073 | 0.0369491 | 1.67E-05 |  | 83,626 | 0.0247 | 0.0212 | 0.2449 |
| 20 | rs117367614 | T | C | 0.36126 | 0.0838906 | 1.66E-05 |  | 83,626 | 0.0384 | 0.0469 | 0.4128 |
| 21 | rs11764137 | T | C | -0.242849 | 0.0576301 | 2.51E-05 |  | 83,626 | -0.008 | 0.0263 | 0.762 |
| 22 | rs11864961 | A | G | -0.183884 | 0.0351212 | 1.64E-07 |  | 83,626 | 0.044 | 0.0256 | 0.0859706 |
| 23 | rs12042286 | A | T | 0.190387 | 0.0428416 | 8.83E-06 |  | 83,626 | -0.0042 | 0.0179 | 0.8127 |
| 24 | rs1273044 | C | T | -0.109143 | 0.0263228 | 3.38E-05 |  | 83,626 | 0.0654 | 0.0456 | 0.1515 |
| 25 | rs12731117 | T | C | -0.147557 | 0.0342711 | 1.67E-05 |  | 83,626 | -0.0154 | 0.0558 | 0.782099 |
| 26 | rs12764494 | A | G | -0.54991 | 0.134203 | 4.17E-05 |  | 83,626 | -0.027 | 0.0238 | 0.2564 |
| 27 | rs12929184 | A | G | -0.160228 | 0.0389966 | 3.98E-05 |  | 83,626 | -0.0131 | 0.0651 | 0.8405 |
| 28 | rs13094101 | A | C | -0.182339 | 0.0379614 | 1.56E-06 |  | 83,626 | 0.0266 | 0.0216 | 0.2171 |
| 29 | rs1362910 | G | A | -0.11021 | 0.0269297 | 4.27E-05 |  | 83,626 | 0.0247 | 0.039 | 0.5272 |
| 30 | rs139670285 | T | C | 0.265025 | 0.0645787 | 4.06E-05 |  | 83,626 | -0.0298 | 0.0226 | 0.1862 |
| 31 | rs144814409 | A | G | -0.935522 | 0.21933 | 2.00E-05 |  | 83,626 | 0.0079 | 0.0214 | 0.7119 |
| 32 | rs145209482 | A | G | 1.50508 | 0.368393 | 4.40E-05 |  | 83,626 | 0.0274 | 0.0211 | 0.1935 |
| 33 | rs147059451 | T | G | 0.264574 | 0.0641234 | 3.69E-05 |  | 83,626 | 0.038 | 0.0216 | 0.0780908 |
| 34 | rs147438061 | A | G | -0.147717 | 0.0336811 | 1.16E-05 |  | 83,626 | -0.0277 | 0.0185 | 0.1345 |
| 35 | rs147512888 | G | A | -0.460906 | 0.108819 | 2.28E-05 |  | 83,626 | -0.0183 | 0.0225 | 0.4162 |
| 36 | rs147761221 | A | G | -0.412908 | 0.0875558 | 2.41E-06 |  | 83,626 | 0.0438 | 0.0228 | 0.0545796 |
| 37 | rs147764711 | G | A | 0.284803 | 0.0664584 | 1.82E-05 |  | 83,626 | -0.0289 | 0.0201 | 0.1507 |
| 38 | rs148253594 | C | T | 0.512693 | 0.123652 | 3.38E-05 |  | 83,626 | -0.048 | 0.0214 | 0.02488 |
| 39 | rs1506001 | A | G | -0.169846 | 0.0384237 | 9.85E-06 |  | 83,626 | 0.0137 | 0.0193 | 0.4785 |
| 40 | rs17053171 | T | C | 0.289219 | 0.0688782 | 2.68E-05 |  | 83,626 | -0.004 | 0.0173 | 0.8167 |
| 41 | rs17225380 | A | G | 0.13851 | 0.032863 | 2.50E-05 |  | 83,626 | -0.0026 | 0.0181 | 0.8866 |
| 42 | rs17368930 | A | T | 0.419127 | 0.101686 | 3.76E-05 |  | 83,626 | 0.03 | 0.0178 | 0.0924294 |
| 43 | rs1845130 | T | G | -0.110026 | 0.0266575 | 3.67E-05 |  | 83,626 | 0.0191 | 0.0177 | 0.2808 |
| 44 | rs192336096 | A | G | -1.22335 | 0.289787 | 2.43E-05 |  | 83,626 | -0.0493 | 0.0294 | 0.0937195 |
| 45 | rs1975254 | C | T | 0.132761 | 0.0312117 | 2.10E-05 |  | 83,626 | 8.00E-04 | 0.0311 | 0.9792 |
| 46 | rs2249245 | A | G | -0.155072 | 0.0312091 | 6.74E-07 |  | 83,626 | -0.0164 | 0.0295 | 0.578699 |
| 47 | rs2554818 | A | G | 0.143588 | 0.0328117 | 1.21E-05 |  | 83,626 | -0.0192 | 0.0198 | 0.3335 |
| 48 | rs35277443 | A | C | -0.169386 | 0.0348745 | 1.19E-06 |  | 83,626 | 0.0323 | 0.0389 | 0.4058 |
| 49 | rs36076286 | G | A | 0.118192 | 0.0285824 | 3.55E-05 |  | 83,626 | 0.0468 | 0.0308 | 0.1285 |
| 50 | rs3741475 | A | G | 0.139951 | 0.0341593 | 4.19E-05 |  | 83,626 | -0.0459 | 0.073 | 0.5297 |
| 51 | rs3741578 | C | G | -0.297837 | 0.0729471 | 4.45E-05 |  | 83,626 | -0.0906 | 0.0457 | 0.0475697 |
| 52 | rs3956254 | T | C | -0.154452 | 0.0349614 | 9.97E-06 |  | 83,626 | -9.00E-04 | 0.0279 | 0.9741 |
| 53 | rs4410329 | A | G | -0.123388 | 0.0284311 | 1.43E-05 |  | 83,626 | 0.0198 | 0.0233 | 0.3954 |
| 54 | rs4536683 | A | G | 0.153157 | 0.0350246 | 1.23E-05 |  | 83,626 | 0.0031 | 0.0271 | 0.9077 |
| 55 | rs4548693 | C | A | -0.123961 | 0.0296464 | 2.90E-05 |  | 83,626 | 0.0339 | 0.0906 | 0.7083 |
| 56 | rs4558785 | T | A | -0.127557 | 0.0299171 | 2.01E-05 |  | 83,626 | 0.0278 | 0.0351 | 0.429 |
| 57 | rs4683357 | G | A | -0.125441 | 0.0270408 | 3.50E-06 |  | 83,626 | 0.112 | 0.0687 | 0.1031 |
| 58 | rs4787028 | C | T | 0.114579 | 0.0275383 | 3.17E-05 |  | 83,626 | 0.0191 | 0.0428 | 0.655901 |
| 59 | rs559398226 | C | T | 0.565271 | 0.130732 | 1.53E-05 |  | 83,626 | -0.0414 | 0.027 | 0.125 |
| 60 | rs56078972 | G | T | -0.111841 | 0.0262733 | 2.07E-05 |  | 83,626 | -0.0497 | 0.0454 | 0.2735 |
| 61 | rs587351 | C | A | -0.112457 | 0.0262745 | 1.87E-05 |  | 83,626 | -0.0052 | 0.0532 | 0.9225 |
| 62 | rs61748881 | G | T | -0.31663 | 0.0564936 | 2.09E-08 |  | 83,626 | -0.0283 | 0.0675 | 0.6751 |
| 63 | rs62096966 | G | T | 0.16496 | 0.0396832 | 3.23E-05 |  | 83,626 | 0.0212 | 0.0473 | 0.6534 |
| 64 | rs643089 | G | T | 0.229217 | 0.055854 | 4.06E-05 |  | 83,626 | -0.089 | 0.0666 | 0.1812 |
| 65 | rs678246 | G | T | -0.191238 | 0.0451704 | 2.30E-05 |  | 83,626 | 0.0266 | 0.0177 | 0.1336 |
| 66 | rs7080218 | A | G | -0.120927 | 0.0297889 | 4.92E-05 |  | 83,626 | 0.014 | 0.0252 | 0.5798 |
| 67 | rs71385313 | G | T | 0.276722 | 0.0677608 | 4.43E-05 |  | 83,626 | 0.0017 | 0.0439 | 0.9696 |
| 68 | rs72637580 | C | T | 0.277385 | 0.0599411 | 3.70E-06 |  | 83,626 | 0.0262 | 0.0723 | 0.717 |
| 69 | rs72827609 | A | G | -0.461125 | 0.111496 | 3.54E-05 |  | 83,626 | -0.0118 | 0.0176 | 0.5004 |
| 70 | rs73057373 | T | C | 0.193197 | 0.0471536 | 4.18E-05 |  | 83,626 | 0.0067 | 0.0175 | 0.703301 |
| 71 | rs73200307 | T | C | -0.158719 | 0.0382272 | 3.30E-05 |  | 83,626 | 0.0106 | 0.019 | 0.578801 |
| 72 | rs74516805 | A | G | 0.369337 | 0.0885093 | 3.01E-05 |  | 83,626 | 0.0056 | 0.0201 | 0.780301 |
| 73 | rs9971308 | G | A | 0.136043 | 0.0323547 | 2.61E-05 |  | 83,626 | -0.0077 | 0.0208 | 0.7114 |

SNP, single nucleotide polymorphism; EA, effect allele; OA, other allele; SE, standard error; PTSD, post-traumatic stress disorder.

**Supplementary Table S1.6** Information of identified SNPs in exposure (PTSD) and outcomes (Smoking status).

|  |  | | | **Exposure (PTSD)** | | |  | **Outcome (Smoking status)** | | | |
| --- | --- | --- | --- | --- | --- | --- | --- | --- | --- | --- | --- |
|  | **SNP** | **EA** | **OA** | **β** | **SE** | ***p* value** |  | **Samplesize** | **β** | **SE** | ***p* value** |
| 1 | rs1024510 | G | A | -0.219903 | 0.0538123 | 4.38E-05 |  | 468,170 | 0.00122627 | 0.00211266 | 0.5 |
| 2 | rs1032419 | C | T | -0.145257 | 0.0355232 | 4.33E-05 |  | 468,170 | -0.00100209 | 0.001799 | 0.59 |
| 3 | rs10512159 | A | G | 0.159327 | 0.0366852 | 1.40E-05 |  | 468,170 | 0.000740114 | 0.00196224 | 0.7 |
| 4 | rs10857816 | G | A | 0.109934 | 0.0261106 | 2.55E-05 |  | 468,170 | -0.00130938 | 0.001403 | 0.31 |
| 5 | rs10938438 | C | T | 0.129063 | 0.0294969 | 1.21E-05 |  | 468,170 | 0.00151249 | 0.00144901 | 0.29 |
| 6 | rs10982385 | G | T | 0.11 | 0.0264619 | 3.23E-05 |  | 468,170 | 0.000819536 | 0.00140029 | 0.55 |
| 7 | rs11244418 | C | T | -0.225322 | 0.0515111 | 1.22E-05 |  | 468,170 | 0.000614752 | 0.00333995 | 0.85 |
| 8 | rs112463264 | T | G | 0.303551 | 0.0734204 | 3.56E-05 |  | 468,170 | 0.0017401 | 0.00410639 | 0.64 |
| 9 | rs112685439 | C | T | -0.304907 | 0.062345 | 1.01E-06 |  | 468,170 | 0.00135852 | 0.00293752 | 0.630001 |
| 10 | rs113457140 | C | G | 0.283916 | 0.0666363 | 2.04E-05 |  | 468,170 | -0.0031239 | 0.00936284 | 0.68 |
| 11 | rs113968366 | T | C | -0.513073 | 0.125182 | 4.16E-05 |  | 468,170 | 0.000938827 | 0.00609812 | 0.84 |
| 12 | rs113986839 | T | G | 0.265891 | 0.0603691 | 1.06E-05 |  | 468,170 | 0.010382 | 0.00454997 | 0.0230001 |
| 13 | rs114850138 | G | A | 0.429768 | 0.0922631 | 3.19E-06 |  | 468,170 | -0.00120437 | 0.00468958 | 0.760001 |
| 14 | rs115567710 | A | G | 0.418466 | 0.101522 | 3.76E-05 |  | 468,170 | -0.0102692 | 0.00762598 | 0.2 |
| 15 | rs1160219 | C | T | 0.142632 | 0.0331717 | 1.71E-05 |  | 468,170 | 0.00271831 | 0.00166971 | 0.12 |
| 16 | rs116174051 | C | T | 0.535461 | 0.119247 | 7.11E-06 |  | 468,170 | -0.00611206 | 0.0053001 | 0.28 |
| 17 | rs11719094 | T | G | 0.159073 | 0.0369491 | 1.67E-05 |  | 468,170 | 0.00220226 | 0.0019266 | 0.22 |
| 18 | rs117367614 | T | C | 0.36126 | 0.0838906 | 1.66E-05 |  | 468,170 | 0.00577325 | 0.0041551 | 0.14 |
| 19 | rs11764137 | T | C | -0.242849 | 0.0576301 | 2.51E-05 |  | 468,170 | -0.00166882 | 0.00337582 | 0.649999 |
| 20 | rs11864961 | A | G | -0.183884 | 0.0351212 | 1.64E-07 |  | 468,170 | 0.00504489 | 0.00202615 | 0.012 |
| 21 | rs12042286 | A | T | 0.190387 | 0.0428416 | 8.83E-06 |  | 468,170 | 0.00226806 | 0.00256712 | 0.38 |
| 22 | rs12731117 | T | C | -0.147557 | 0.0342711 | 1.67E-05 |  | 468,170 | -7.13E-05 | 0.00159702 | 0.92 |
| 23 | rs12764494 | A | G | -0.54991 | 0.134203 | 4.17E-05 |  | 468,170 | 0.00205591 | 0.00370212 | 0.56 |
| 24 | rs12929184 | A | G | -0.160228 | 0.0389966 | 3.98E-05 |  | 468,170 | -0.0049162 | 0.00215546 | 0.025 |
| 25 | rs13094101 | A | C | -0.182339 | 0.0379614 | 1.56E-06 |  | 468,170 | 0.00191003 | 0.00181532 | 0.28 |
| 26 | rs139670285 | T | C | 0.265025 | 0.0645787 | 4.06E-05 |  | 468,170 | 0.000597383 | 0.00327111 | 0.96 |
| 27 | rs144814409 | A | G | -0.935522 | 0.21933 | 2.00E-05 |  | 468,170 | -0.00250832 | 0.0089455 | 0.8 |
| 28 | rs145209482 | A | G | 1.50508 | 0.368393 | 4.40E-05 |  | 468,170 | -0.00977864 | 0.0102381 | 0.33 |
| 29 | rs147059451 | T | G | 0.264574 | 0.0641234 | 3.69E-05 |  | 468,170 | -0.00413276 | 0.00463643 | 0.35 |
| 30 | rs147438061 | A | G | -0.147717 | 0.0336811 | 1.16E-05 |  | 468,170 | -0.000546992 | 0.00187297 | 0.780001 |
| 31 | rs147512888 | G | A | -0.460906 | 0.108819 | 2.28E-05 |  | 468,170 | 0.0121909 | 0.00575569 | 0.0299999 |
| 32 | rs147761221 | A | G | -0.412908 | 0.0875558 | 2.41E-06 |  | 468,170 | -4.44E-05 | 0.00776367 | 0.97 |
| 33 | rs147764711 | G | A | 0.284803 | 0.0664584 | 1.82E-05 |  | 468,170 | -0.0114973 | 0.00524729 | 0.0309999 |
| 34 | rs148253594 | C | T | 0.512693 | 0.123652 | 3.38E-05 |  | 468,170 | 0.00207681 | 0.00630512 | 0.75 |
| 35 | rs1506001 | A | G | -0.169846 | 0.0384237 | 9.85E-06 |  | 468,170 | 0.000229506 | 0.00176766 | 0.99 |
| 36 | rs17053171 | T | C | 0.289219 | 0.0688782 | 2.68E-05 |  | 468,170 | -0.00530443 | 0.00306071 | 0.0940005 |
| 37 | rs17225380 | A | G | 0.13851 | 0.032863 | 2.50E-05 |  | 468,170 | 0.00173283 | 0.00172079 | 0.27 |
| 38 | rs17368930 | A | T | 0.419127 | 0.101686 | 3.76E-05 |  | 468,170 | -0.000759643 | 0.00497619 | 0.85 |
| 39 | rs1845130 | T | G | -0.110026 | 0.0266575 | 3.67E-05 |  | 468,170 | 0.00162252 | 0.00146637 | 0.23 |
| 40 | rs192336096 | A | G | -1.22335 | 0.289787 | 2.43E-05 |  | 468,170 | 0.00443061 | 0.00547713 | 0.47 |
| 41 | rs1975254 | C | T | 0.132761 | 0.0312117 | 2.10E-05 |  | 468,170 | -0.00299595 | 0.00182169 | 0.12 |
| 42 | rs2554818 | A | G | 0.143588 | 0.0328117 | 1.21E-05 |  | 468,170 | -0.00173453 | 0.00158049 | 0.27 |
| 43 | rs35277443 | A | C | -0.169386 | 0.0348745 | 1.19E-06 |  | 468,170 | -0.000829861 | 0.00167006 | 0.62 |
| 44 | rs36076286 | G | A | 0.118192 | 0.0285824 | 3.55E-05 |  | 468,170 | -0.000811825 | 0.00146511 | 0.55 |
| 45 | rs3741475 | A | G | 0.139951 | 0.0341593 | 4.19E-05 |  | 468,170 | 0.00271562 | 0.001729 | 0.12 |
| 46 | rs3741578 | C | G | -0.297837 | 0.0729471 | 4.45E-05 |  | 468,170 | 0.00128693 | 0.0024362 | 0.6 |
| 47 | rs3956254 | T | C | -0.154452 | 0.0349614 | 9.97E-06 |  | 468,170 | 0.00247074 | 0.00193277 | 0.19 |
| 48 | rs4410329 | A | G | -0.123388 | 0.0284311 | 1.43E-05 |  | 468,170 | 0.000167252 | 0.00165677 | 0.91 |
| 49 | rs4548693 | C | A | -0.123961 | 0.0296464 | 2.90E-05 |  | 468,170 | -0.00178276 | 0.00152233 | 0.23 |
| 50 | rs4558785 | T | A | -0.127557 | 0.0299171 | 2.01E-05 |  | 468,170 | 0.00170416 | 0.0015665 | 0.26 |
| 51 | rs4683357 | G | A | -0.125441 | 0.0270408 | 3.50E-06 |  | 468,170 | -0.00171769 | 0.00138523 | 0.22 |
| 52 | rs4787028 | C | T | 0.114579 | 0.0275383 | 3.17E-05 |  | 468,170 | -0.000641526 | 0.0014526 | 0.649999 |
| 53 | rs587351 | C | A | -0.112457 | 0.0262745 | 1.87E-05 |  | 468,170 | -0.000793389 | 0.00137463 | 0.53 |
| 54 | rs61748881 | G | T | -0.31663 | 0.0564936 | 2.09E-08 |  | 468,170 | 0.00128308 | 0.0021114 | 0.53 |
| 55 | rs62096966 | G | T | 0.16496 | 0.0396832 | 3.23E-05 |  | 468,170 | -0.00150767 | 0.00175689 | 0.37 |
| 56 | rs643089 | G | T | 0.229217 | 0.055854 | 4.06E-05 |  | 468,170 | 0.00162125 | 0.00232781 | 0.54 |
| 57 | rs678246 | G | T | -0.191238 | 0.0451704 | 2.30E-05 |  | 468,170 | -0.00350887 | 0.00227647 | 0.13 |
| 58 | rs7080218 | A | G | -0.120927 | 0.0297889 | 4.92E-05 |  | 468,170 | 0.00159036 | 0.00153419 | 0.31 |
| 59 | rs71385313 | G | T | 0.276722 | 0.0677608 | 4.43E-05 |  | 468,170 | -0.00433243 | 0.00355869 | 0.26 |
| 60 | rs72637580 | C | T | 0.277385 | 0.0599411 | 3.70E-06 |  | 468,170 | -0.00123495 | 0.00249896 | 0.61 |
| 61 | rs72827609 | A | G | -0.461125 | 0.111496 | 3.54E-05 |  | 468,170 | -0.00179936 | 0.00355381 | 0.6 |
| 62 | rs73057373 | T | C | 0.193197 | 0.0471536 | 4.18E-05 |  | 468,170 | 0.000438423 | 0.00255928 | 0.86 |
| 63 | rs73200307 | T | C | -0.158719 | 0.0382272 | 3.30E-05 |  | 468,170 | 0.00161956 | 0.00211538 | 0.46 |
| 64 | rs74516805 | A | G | 0.369337 | 0.0885093 | 3.01E-05 |  | 468,170 | 0.00189716 | 0.00475319 | 0.7 |
| 65 | rs74812538 | C | T | 0.390509 | 0.0918243 | 2.11E-05 |  | 468,170 | -0.000316645 | 0.00468271 | 0.9 |
| 66 | rs74863973 | C | T | -0.144354 | 0.0346156 | 3.04E-05 |  | 468,170 | 0.00120397 | 0.00225126 | 0.58 |
| 67 | rs75598025 | G | A | 0.520541 | 0.119165 | 1.25E-05 |  | 468,170 | -0.000824369 | 0.00708237 | 0.82 |
| 68 | rs7562028 | A | G | -0.360709 | 0.082574 | 1.25E-05 |  | 468,170 | -0.000143981 | 0.00260175 | 0.94 |
| 69 | rs75641198 | T | C | 0.274826 | 0.0575213 | 1.77E-06 |  | 468,170 | -0.0028505 | 0.00260381 | 0.26 |
| 70 | rs75928930 | C | T | 0.281701 | 0.0672459 | 2.80E-05 |  | 468,170 | 0.00461055 | 0.0043078 | 0.29 |
| 71 | rs76355089 | G | A | 0.249634 | 0.0602865 | 3.46E-05 |  | 468,170 | -8.74E-05 | 0.00339369 | 0.97 |
| 72 | rs76404313 | G | A | 1.27887 | 0.314601 | 4.80E-05 |  | 468,170 | 0.00441555 | 0.0068519 | 0.54 |
| 73 | rs769018 | T | C | 0.190415 | 0.0459608 | 3.43E-05 |  | 468,170 | 0.00220194 | 0.00201957 | 0.28 |
| 74 | rs78791514 | T | C | -0.473445 | 0.115499 | 4.15E-05 |  | 468,170 | 0.00205982 | 0.00364785 | 0.58 |
| 75 | rs79142281 | A | C | -0.467126 | 0.110791 | 2.48E-05 |  | 468,170 | -0.0018966 | 0.00417836 | 0.68 |
| 76 | rs79433303 | T | G | 0.660108 | 0.147299 | 7.41E-06 |  | 468,170 | 0.00427939 | 0.00496842 | 0.450001 |
| 77 | rs79968379 | G | A | -0.467312 | 0.110772 | 2.46E-05 |  | 468,170 | 6.56E-05 | 0.00303642 | 0.96 |
| 78 | rs80021344 | G | A | 0.718864 | 0.168948 | 2.09E-05 |  | 468,170 | -0.00256273 | 0.00423697 | 0.55 |
| 79 | rs8003680 | T | C | 0.121925 | 0.0277197 | 1.09E-05 |  | 468,170 | 0.00254183 | 0.00138689 | 0.0599998 |
| 80 | rs80038448 | T | C | 0.18611 | 0.0403734 | 4.03E-06 |  | 468,170 | 0.00182381 | 0.00193579 | 0.39 |
| 81 | rs80261117 | A | G | 0.3007 | 0.0671 | 7.42E-06 |  | 468,170 | -0.00713503 | 0.00310962 | 0.0269998 |
| 82 | rs868209 | A | T | -0.168971 | 0.0395824 | 1.96E-05 |  | 468,170 | -0.00144332 | 0.0017273 | 0.41 |
| 83 | rs9453417 | A | G | 0.458284 | 0.106145 | 1.58E-05 |  | 468,170 | -0.00636128 | 0.00586987 | 0.27 |
| 84 | rs9526717 | G | A | 0.125284 | 0.0270735 | 3.70E-06 |  | 468,170 | -0.00108443 | 0.00141381 | 0.51 |
| 85 | rs9809585 | G | A | -0.108248 | 0.026432 | 4.22E-05 |  | 468,170 | 0.000253985 | 0.00138428 | 0.81 |
| 86 | rs9900367 | C | T | -0.131089 | 0.0285907 | 4.54E-06 |  | 468,170 | 0.0013332 | 0.00148394 | 0.35 |
| 87 | rs9968731 | T | C | -0.124696 | 0.0299353 | 3.11E-05 |  | 468,170 | 0.00337119 | 0.00145633 | 0.0219999 |
| 88 | rs9971308 | G | A | 0.136043 | 0.0323547 | 2.61E-05 |  | 468,170 | -0.00187702 | 0.00169795 | 0.3 |

SNP, single nucleotide polymorphism; EA, effect allele; OA, other allele; SE, standard error; PTSD, post-traumatic stress disorder.

**Supplementary Table S1.7** Information of identified SNPs in exposure (Obesity) and outcomes (T2D).

|  |  | | | **Exposure (Obesity)** | | |  | **Outcome (T2D)** | | | | |
| --- | --- | --- | --- | --- | --- | --- | --- | --- | --- | --- | --- | --- |
|  | **SNP** | **EA** | **OA** | **β** | **SE** | ***p* value** |  | **Case** | **Control** | **β** | **SE** | ***p* value** |
| 1 | rs10145461 | G | T | 0.0525398 | 0.00894147 | 4.20E-09 |  | 12,931 | 57,196 | 0.0207 | 0.0159 | 0.1929 |
| 2 | rs10860990 | C | T | -0.0509467 | 0.00915491 | 2.62E-08 |  | 12,931 | 57,196 | -0.011 | 0.0163 | 0.4978 |
| 3 | rs10879428 | C | A | 0.0619012 | 0.010758 | 8.72E-09 |  | 12,931 | 57,196 | 0.0054 | 0.0205 | 0.7914 |
| 4 | rs10929985 | T | C | 0.0540452 | 0.00901485 | 2.03E-09 |  | 12,931 | 57,196 | 0.0378 | 0.0158 | 0.0166702 |
| 5 | rs10938398 | A | G | 0.0874448 | 0.00887577 | 6.71E-23 |  | 12,931 | 57,196 | 0.018 | 0.0158 | 0.256 |
| 6 | rs11030104 | G | A | -0.100292 | 0.0121661 | 1.67E-16 |  | 12,931 | 57,196 | -0.0255 | 0.0195 | 0.1905 |
| 7 | rs11642015 | T | C | 0.190462 | 0.00887448 | 3.55E-102 |  | 12,931 | 57,196 | 0.0426 | 0.0157 | 0.00668806 |
| 8 | rs12511535 | T | C | -0.0536745 | 0.0091559 | 4.57E-09 |  | 12,931 | 57,196 | 0.0031 | 0.0164 | 0.8517 |
| 9 | rs12831874 | T | G | -0.0766609 | 0.0127064 | 1.61E-09 |  | 12,931 | 57,196 | 0.025 | 0.0243 | 0.3018 |
| 10 | rs12928404 | C | T | 0.0589756 | 0.00893363 | 4.07E-11 |  | 12,931 | 57,196 | -0.0086 | 0.0162 | 0.5954 |
| 11 | rs12956821 | G | T | -0.0588749 | 0.00929662 | 2.41E-10 |  | 12,931 | 57,196 | -0.0064 | 0.0169 | 0.704601 |
| 12 | rs13296413 | T | C | -0.0577256 | 0.00972666 | 2.94E-09 |  | 12,931 | 57,196 | -0.0222 | 0.0162 | 0.1721 |
| 13 | rs1429935 | C | A | -0.0595548 | 0.00889159 | 2.11E-11 |  | 12,931 | 57,196 | 0.0047 | 0.0158 | 0.7685 |
| 14 | rs17024258 | T | C | 0.124289 | 0.018246 | 9.64E-12 |  | 12,931 | 57,196 | 0.0661 | 0.0467 | 0.1565 |
| 15 | rs1861410 | T | C | -0.0601854 | 0.00900153 | 2.29E-11 |  | 12,931 | 57,196 | 0.0098 | 0.0158 | 0.5361 |
| 16 | rs186989 | G | A | 0.0593946 | 0.0105152 | 1.62E-08 |  | 12,931 | 57,196 | -0.0231 | 0.0174 | 0.1833 |
| 17 | rs2035260 | A | C | 0.0732823 | 0.0110826 | 3.78E-11 |  | 12,931 | 57,196 | 0.0018 | 0.0178 | 0.9177 |
| 18 | rs2168711 | C | T | 0.108166 | 0.0111765 | 3.74E-22 |  | 12,931 | 57,196 | 0.0166 | 0.0183 | 0.3646 |
| 19 | rs2659007 | A | G | 0.0511162 | 0.00891927 | 9.98E-09 |  | 12,931 | 57,196 | -0.0098 | 0.0271 | 0.717799 |
| 20 | rs28814423 | A | G | 0.057011 | 0.00949489 | 1.92E-09 |  | 12,931 | 57,196 | 0.0082 | 0.0173 | 0.635801 |
| 21 | rs34298980 | C | T | -0.0622097 | 0.00961315 | 9.72E-11 |  | 12,931 | 57,196 | -0.034 | 0.0167 | 0.0425305 |
| 22 | rs346237 | C | T | -0.0582915 | 0.00922476 | 2.63E-10 |  | 12,931 | 57,196 | -0.0181 | 0.0164 | 0.2718 |
| 23 | rs34783010 | T | G | -0.0767011 | 0.0102727 | 8.24E-14 |  | 12,931 | 57,196 | 0.0804 | 0.0228 | 0.000425696 |
| 24 | rs3747973 | G | A | -0.0503581 | 0.00907877 | 2.91E-08 |  | 12,931 | 57,196 | 0.0021 | 0.016 | 0.8931 |
| 25 | rs3798519 | C | A | 0.0971224 | 0.0106521 | 7.68E-20 |  | 12,931 | 57,196 | 0.0475 | 0.02 | 0.0176299 |
| 26 | rs3809627 | A | C | -0.0513707 | 0.00887638 | 7.15E-09 |  | 12,931 | 57,196 | -0.0149 | 0.0177 | 0.3988 |
| 27 | rs4072287 | A | C | 0.053879 | 0.00909349 | 3.12E-09 |  | 12,931 | 57,196 | -0.0351 | 0.0402 | 0.3814 |
| 28 | rs45551238 | T | C | -0.139042 | 0.0214124 | 8.38E-11 |  | 12,931 | 57,196 | 0.0779 | 0.0793 | 0.3256 |
| 29 | rs4776970 | T | A | -0.0539283 | 0.00936044 | 8.35E-09 |  | 12,931 | 57,196 | 0.0252 | 0.0163 | 0.1215 |
| 30 | rs545608 | C | G | 0.0761439 | 0.0113769 | 2.19E-11 |  | 12,931 | 57,196 | 0.0063 | 0.0198 | 0.7503 |
| 31 | rs60764613 | T | G | 0.0818851 | 0.0114914 | 1.03E-12 |  | 12,931 | 57,196 | 0.0083 | 0.0221 | 0.7076 |
| 32 | rs62107261 | C | T | -0.207366 | 0.033749 | 8.03E-10 |  | 12,931 | 57,196 | -0.0223 | 0.0393 | 0.5707 |
| 33 | rs62473704 | A | C | -0.106212 | 0.0109831 | 4.02E-22 |  | 12,931 | 57,196 | 0.021 | 0.0251 | 0.4025 |
| 34 | rs6726297 | G | A | 0.0643523 | 0.0102791 | 3.84E-10 |  | 12,931 | 57,196 | 7.00E-04 | 0.0183 | 0.9678 |
| 35 | rs6739303 | T | C | 0.12742 | 0.0122823 | 3.25E-25 |  | 12,931 | 57,196 | -0.0014 | 0.0207 | 0.9476 |
| 36 | rs6749170 | G | A | 0.0537046 | 0.00898358 | 2.26E-09 |  | 12,931 | 57,196 | -0.0023 | 0.0158 | 0.886 |
| 37 | rs6966722 | A | G | 0.118542 | 0.0207806 | 1.17E-08 |  | 12,931 | 57,196 | -0.016 | 0.0316 | 0.6122 |
| 38 | rs7324697 | A | C | -0.0661732 | 0.00940134 | 1.94E-12 |  | 12,931 | 57,196 | -0.0117 | 0.0167 | 0.4834 |
| 39 | rs7531118 | C | T | 0.0531995 | 0.00905337 | 4.20E-09 |  | 12,931 | 57,196 | 0.0039 | 0.0161 | 0.8096 |
| 40 | rs78459567 | G | A | 0.111714 | 0.0197182 | 1.47E-08 |  | 12,931 | 57,196 | -0.0087 | 0.037 | 0.8145 |
| 41 | rs78485941 | A | G | 0.150434 | 0.0266527 | 1.66E-08 |  | 12,931 | 57,196 | -0.0286 | 0.0467 | 0.5401 |
| 42 | rs7862029 | T | G | -0.0505698 | 0.00899087 | 1.86E-08 |  | 12,931 | 57,196 | -0.006 | 0.0159 | 0.7039 |
| 43 | rs8057911 | T | C | 0.0628601 | 0.0110547 | 1.30E-08 |  | 12,931 | 57,196 | 9.00E-04 | 0.0188 | 0.9627 |
| 44 | rs8074621 | C | T | 0.0548374 | 0.00889266 | 6.98E-10 |  | 12,931 | 57,196 | 0.0103 | 0.016 | 0.5202 |
| 45 | rs9823425 | C | T | 0.0560252 | 0.00901777 | 5.21E-10 |  | 12,931 | 57,196 | 0.0075 | 0.0157 | 0.633299 |
| 46 | rs9856151 | A | G | 0.0548632 | 0.00998061 | 3.86E-08 |  | 12,931 | 57,196 | 0.0282 | 0.0176 | 0.1085 |

SNP, single nucleotide polymorphism; EA, effect allele; OA, other allele; SE, standard error; T2D, type 2 diabetes.

**Supplementary Table S1.8** Information of identified SNPs in exposure (Hypertension) and outcomes (T2D).

|  |  | | | **Exposure (Hypertension)** | | |  | **Outcome (T2D)** | | | | |
| --- | --- | --- | --- | --- | --- | --- | --- | --- | --- | --- | --- | --- |
|  | **SNP** | **EA** | **OA** | **β** | **SE** | ***p* value** |  | **Case** | **Control** | **β** | **SE** | ***p* value** |
| 1 | rs10059884 | A | C | 0.0129429 | 0.000914148 | 1.66E-45 |  | 12,931 | 57,196 | -0.0013 | 0.016 | 0.9374 |
| 2 | rs10059921 | T | G | -0.011882 | 0.00170987 | 3.68E-12 |  | 12,931 | 57,196 | 0.008 | 0.0287 | 0.7801 |
| 3 | rs10061288 | G | A | -0.00717078 | 0.000897735 | 1.38E-15 |  | 12,931 | 57,196 | 0.0254 | 0.0157 | 0.1059 |
| 4 | rs10282122 | T | C | -0.00656527 | 0.000951716 | 5.26E-12 |  | 12,931 | 57,196 | 0.0335 | 0.0293 | 0.2524 |
| 5 | rs10457174 | T | C | 0.00653277 | 0.00113551 | 8.76E-09 |  | 12,931 | 57,196 | -0.0111 | 0.0197 | 0.574399 |
| 6 | rs1048070 | C | T | 0.00515096 | 0.000917968 | 2.01E-08 |  | 12,931 | 57,196 | 0.0147 | 0.0163 | 0.3679 |
| 7 | rs10491475 | A | C | -0.00508215 | 0.000906733 | 2.08E-08 |  | 12,931 | 57,196 | -0.0012 | 0.0158 | 0.9395 |
| 8 | rs10500326 | T | G | -0.00769248 | 0.00105717 | 3.43E-13 |  | 12,931 | 57,196 | -0.0046 | 0.0183 | 0.8027 |
| 9 | rs10832586 | C | A | 0.0101316 | 0.00111568 | 1.07E-19 |  | 12,931 | 57,196 | 0.006 | 0.0195 | 0.759 |
| 10 | rs10900127 | A | C | -0.0057941 | 0.000915326 | 2.45E-10 |  | 12,931 | 57,196 | -0.0364 | 0.0157 | 0.02044 |
| 11 | rs10930990 | G | C | -0.00602194 | 0.00096762 | 4.86E-10 |  | 12,931 | 57,196 | 0.0267 | 0.0171 | 0.1199 |
| 12 | rs11055034 | A | C | -0.0058341 | 0.00100178 | 5.75E-09 |  | 12,931 | 57,196 | -0.0022 | 0.0179 | 0.9034 |
| 13 | rs11072508 | T | C | -0.011038 | 0.000957232 | 9.19E-31 |  | 12,931 | 57,196 | -0.0175 | 0.0167 | 0.2951 |
| 14 | rs11086052 | A | G | 0.00764151 | 0.00100215 | 2.44E-14 |  | 12,931 | 57,196 | -0.0073 | 0.0174 | 0.674099 |
| 15 | rs1115460 | A | G | 0.00526644 | 0.00095581 | 3.59E-08 |  | 12,931 | 57,196 | -0.0156 | 0.0168 | 0.3519 |
| 16 | rs111896658 | T | C | -0.0100829 | 0.00144611 | 3.12E-12 |  | 12,931 | 57,196 | 0.0212 | 0.0251 | 0.3988 |
| 17 | rs11190709 | A | G | 0.00857061 | 0.00143315 | 2.23E-09 |  | 12,931 | 57,196 | -0.0203 | 0.0249 | 0.4157 |
| 18 | rs11191607 | T | G | -0.017465 | 0.00167693 | 2.12E-25 |  | 12,931 | 57,196 | -0.0499 | 0.0279 | 0.0732302 |
| 19 | rs11199851 | C | A | 0.00683369 | 0.00111602 | 9.17E-10 |  | 12,931 | 57,196 | 0.0243 | 0.0192 | 0.2068 |
| 20 | rs11212197 | A | G | 0.00579373 | 0.000958807 | 1.52E-09 |  | 12,931 | 57,196 | -0.0094 | 0.0169 | 0.580399 |
| 21 | rs112767262 | T | C | 0.00798101 | 0.00115522 | 4.89E-12 |  | 12,931 | 57,196 | -0.0524 | 0.021 | 0.0125901 |
| 22 | rs115262049 | T | A | -0.00979727 | 0.00159532 | 8.19E-10 |  | 12,931 | 57,196 | 0.0253 | 0.0299 | 0.3963 |
| 23 | rs11556924 | T | C | -0.00680118 | 0.000920214 | 1.46E-13 |  | 12,931 | 57,196 | -0.0068 | 0.0181 | 0.7061 |
| 24 | rs11616092 | G | A | -0.00615154 | 0.0010088 | 1.07E-09 |  | 12,931 | 57,196 | 0.0089 | 0.0177 | 0.6162 |
| 25 | rs11688682 | C | G | -0.00621807 | 0.00104261 | 2.46E-09 |  | 12,931 | 57,196 | -0.0481 | 0.0196 | 0.0142699 |
| 26 | rs11692391 | C | T | -0.00632584 | 0.00108982 | 6.46E-09 |  | 12,931 | 57,196 | -0.0039 | 0.0192 | 0.8376 |
| 27 | rs11692449 | C | T | -0.00614942 | 0.000928988 | 3.60E-11 |  | 12,931 | 57,196 | 0.0147 | 0.0162 | 0.3622 |
| 28 | rs11724647 | A | T | -0.00940922 | 0.00115908 | 4.75E-16 |  | 12,931 | 57,196 | 0.0267 | 0.0199 | 0.1797 |
| 29 | rs117464403 | A | G | 0.0196768 | 0.00328268 | 2.05E-09 |  | 12,931 | 57,196 | -0.0596 | 0.0599 | 0.3192 |
| 30 | rs11955537 | G | A | -0.00732953 | 0.00129892 | 1.67E-08 |  | 12,931 | 57,196 | 0.0397 | 0.0221 | 0.0721307 |
| 31 | rs12035750 | C | T | 0.00589603 | 0.000923596 | 1.73E-10 |  | 12,931 | 57,196 | 0.0117 | 0.0163 | 0.4743 |
| 32 | rs12258967 | G | C | -0.0106243 | 0.000980732 | 2.40E-27 |  | 12,931 | 57,196 | 0.0031 | 0.0175 | 0.8601 |
| 33 | rs12364503 | G | C | -0.00619261 | 0.000925343 | 2.20E-11 |  | 12,931 | 57,196 | 0.0129 | 0.0163 | 0.4306 |
| 34 | rs12627514 | G | C | 0.00695894 | 0.000998038 | 3.11E-12 |  | 12,931 | 57,196 | -0.0116 | 0.0394 | 0.768 |
| 35 | rs12702586 | A | G | 0.0121916 | 0.00142284 | 1.05E-17 |  | 12,931 | 57,196 | 0.0051 | 0.0241 | 0.8325 |
| 36 | rs12714414 | C | T | -0.00716148 | 0.00129457 | 3.17E-08 |  | 12,931 | 57,196 | -0.0017 | 0.0207 | 0.9344 |
| 37 | rs1274652 | G | C | -0.00738331 | 0.00127736 | 7.46E-09 |  | 12,931 | 57,196 | -0.0129 | 0.0217 | 0.5526 |
| 38 | rs1275988 | T | C | -0.0140941 | 0.000922812 | 1.16E-52 |  | 12,931 | 57,196 | 0.0311 | 0.016 | 0.0519003 |
| 39 | rs12786744 | C | T | -0.0062928 | 0.00109836 | 1.01E-08 |  | 12,931 | 57,196 | -0.0222 | 0.019 | 0.2425 |
| 40 | rs12906962 | C | T | 0.00614902 | 0.000968275 | 2.15E-10 |  | 12,931 | 57,196 | 0.0323 | 0.0167 | 0.0525702 |
| 41 | rs12948326 | G | T | 0.00540283 | 0.000944536 | 1.06E-08 |  | 12,931 | 57,196 | 0.0149 | 0.0166 | 0.3694 |
| 42 | rs13125101 | A | G | 0.0212658 | 0.000988617 | 1.24E-102 |  | 12,931 | 57,196 | -0.0016 | 0.0173 | 0.9251 |
| 43 | rs13257887 | C | T | -0.00619465 | 0.00102872 | 1.73E-09 |  | 12,931 | 57,196 | 0.0093 | 0.0162 | 0.5681 |
| 44 | rs1362877 | T | C | -0.00584575 | 0.000903671 | 9.87E-11 |  | 12,931 | 57,196 | 0.0198 | 0.0157 | 0.2059 |
| 45 | rs1422278 | T | G | 0.00945522 | 0.00132451 | 9.42E-13 |  | 12,931 | 57,196 | 0.0282 | 0.0227 | 0.2135 |
| 46 | rs1436138 | G | A | -0.00739454 | 0.000938222 | 3.24E-15 |  | 12,931 | 57,196 | -0.0197 | 0.0171 | 0.2493 |
| 47 | rs145153053 | G | A | 0.00767235 | 0.0011983 | 1.53E-10 |  | 12,931 | 57,196 | -7.00E-04 | 0.0215 | 0.9734 |
| 48 | rs1623060 | T | C | -0.0050744 | 0.00089802 | 1.60E-08 |  | 12,931 | 57,196 | 0.0029 | 0.0157 | 0.8514 |
| 49 | rs1689040 | T | C | -0.011369 | 0.000913632 | 1.51E-35 |  | 12,931 | 57,196 | 2.00E-04 | 0.016 | 0.9878 |
| 50 | rs1741344 | T | C | 0.00538176 | 0.000931546 | 7.59E-09 |  | 12,931 | 57,196 | 0.0065 | 0.0163 | 0.692 |
| 51 | rs17637472 | A | G | 0.00861819 | 0.000924195 | 1.11E-20 |  | 12,931 | 57,196 | 0.0132 | 0.0161 | 0.4118 |
| 52 | rs17747401 | T | C | -0.00541349 | 0.000935245 | 7.11E-09 |  | 12,931 | 57,196 | -0.0213 | 0.0163 | 0.1905 |
| 53 | rs1799998 | G | A | -0.00546115 | 0.000901191 | 1.36E-09 |  | 12,931 | 57,196 | 0.0167 | 0.0158 | 0.2904 |
| 54 | rs1801253 | C | G | 0.0117536 | 0.00102209 | 1.33E-30 |  | 12,931 | 57,196 | 0.0126 | 0.0178 | 0.4769 |
| 55 | rs1859551 | G | A | 0.00511058 | 0.000900843 | 1.40E-08 |  | 12,931 | 57,196 | 0.0249 | 0.0158 | 0.1137 |
| 56 | rs188315257 | G | T | -0.010802 | 0.00155991 | 4.37E-12 |  | 12,931 | 57,196 | -0.053 | 0.0273 | 0.05236 |
| 57 | rs1887320 | A | G | 0.0106438 | 0.000900496 | 3.08E-32 |  | 12,931 | 57,196 | 0.0104 | 0.0157 | 0.5052 |
| 58 | rs1923031 | C | T | -0.00780776 | 0.000945459 | 1.48E-16 |  | 12,931 | 57,196 | 0.0441 | 0.0383 | 0.2487 |
| 59 | rs1957562 | A | G | 0.00933996 | 0.00101217 | 2.77E-20 |  | 12,931 | 57,196 | 0.0316 | 0.0177 | 0.0751398 |
| 60 | rs198851 | G | T | -0.0129598 | 0.00125459 | 5.16E-25 |  | 12,931 | 57,196 | -0.0305 | 0.0219 | 0.1632 |
| 61 | rs2014590 | T | C | -0.00708324 | 0.000897558 | 2.98E-15 |  | 12,931 | 57,196 | -0.0298 | 0.0157 | 0.0584804 |
| 62 | rs2032915 | T | C | -0.00598366 | 0.000934788 | 1.54E-10 |  | 12,931 | 57,196 | -0.0093 | 0.0166 | 0.5751 |
| 63 | rs204883 | A | G | 0.00778339 | 0.000920418 | 2.76E-17 |  | 12,931 | 57,196 | 0.0274 | 0.0177 | 0.1221 |
| 64 | rs2078339 | G | A | -0.00772915 | 0.00100886 | 1.84E-14 |  | 12,931 | 57,196 | -0.0086 | 0.0177 | 0.6264 |
| 65 | rs2105092 | A | G | -0.00709777 | 0.00098884 | 7.08E-13 |  | 12,931 | 57,196 | -0.0129 | 0.0174 | 0.4607 |
| 66 | rs2293251 | G | T | 0.0068858 | 0.0012357 | 2.51E-08 |  | 12,931 | 57,196 | 0.0499 | 0.0215 | 0.0205499 |
| 67 | rs2294214 | C | A | -0.0055795 | 0.000963432 | 6.99E-09 |  | 12,931 | 57,196 | 0.0222 | 0.0169 | 0.1892 |
| 68 | rs2298359 | C | T | -0.0131885 | 0.00181983 | 4.26E-13 |  | 12,931 | 57,196 | -0.1585 | 0.0764 | 0.0382103 |
| 69 | rs2311412 | C | T | -0.00530779 | 0.000899501 | 3.62E-09 |  | 12,931 | 57,196 | 0.0049 | 0.0157 | 0.756201 |
| 70 | rs231708 | C | G | -0.00573449 | 0.000967339 | 3.06E-09 |  | 12,931 | 57,196 | 0.0336 | 0.0169 | 0.0467498 |
| 71 | rs2341599 | A | G | -0.00701927 | 0.000947893 | 1.31E-13 |  | 12,931 | 57,196 | -0.0032 | 0.0165 | 0.8455 |
| 72 | rs2443708 | C | T | 0.00728822 | 0.000966422 | 4.65E-14 |  | 12,931 | 57,196 | 0.0291 | 0.0168 | 0.0837992 |
| 73 | rs2460448 | A | G | -0.00808064 | 0.000906743 | 5.02E-19 |  | 12,931 | 57,196 | 0.0038 | 0.0168 | 0.8202 |
| 74 | rs2493135 | G | C | 0.00787741 | 0.000914021 | 6.79E-18 |  | 12,931 | 57,196 | -0.0169 | 0.0159 | 0.2892 |
| 75 | rs2569882 | C | T | -0.00579055 | 0.000910921 | 2.06E-10 |  | 12,931 | 57,196 | -0.0141 | 0.0192 | 0.4637 |
| 76 | rs2643826 | T | C | 0.0106264 | 0.000903762 | 6.43E-32 |  | 12,931 | 57,196 | -0.0049 | 0.0158 | 0.7565 |
| 77 | rs268263 | A | T | 0.007854 | 0.00104552 | 5.82E-14 |  | 12,931 | 57,196 | 0.0011 | 0.0185 | 0.9516 |
| 78 | rs2744133 | G | A | -0.00653248 | 0.00100191 | 7.03E-11 |  | 12,931 | 57,196 | 0.0117 | 0.0175 | 0.5027 |
| 79 | rs27687 | C | T | 0.00608825 | 0.00101038 | 1.68E-09 |  | 12,931 | 57,196 | 0.0071 | 0.0179 | 0.691301 |
| 80 | rs2820290 | G | A | 0.00575237 | 0.000904397 | 2.01E-10 |  | 12,931 | 57,196 | 0.0423 | 0.0158 | 0.00741396 |
| 81 | rs2823139 | A | G | 0.00602667 | 0.000955103 | 2.79E-10 |  | 12,931 | 57,196 | -0.0243 | 0.0167 | 0.1449 |
| 82 | rs2856653 | C | T | -0.00902048 | 0.00093971 | 8.06E-22 |  | 12,931 | 57,196 | -0.0014 | 0.0165 | 0.9322 |
| 83 | rs28667801 | T | A | 0.00635567 | 0.000917697 | 4.34E-12 |  | 12,931 | 57,196 | 0.0129 | 0.0164 | 0.4305 |
| 84 | rs2894446 | T | G | -0.00673875 | 0.000946932 | 1.11E-12 |  | 12,931 | 57,196 | 0.0243 | 0.0164 | 0.14 |
| 85 | rs2921965 | C | T | -0.00568897 | 0.00099864 | 1.22E-08 |  | 12,931 | 57,196 | -0.0497 | 0.018 | 0.00570204 |
| 86 | rs2934849 | C | T | -0.00560546 | 0.000929599 | 1.64E-09 |  | 12,931 | 57,196 | 0.0017 | 0.0164 | 0.916 |
| 87 | rs2943810 | G | C | 0.00629417 | 0.001039 | 1.38E-09 |  | 12,931 | 57,196 | 0.0054 | 0.0202 | 0.788 |
| 88 | rs2977324 | G | T | 0.0071587 | 0.0009839 | 3.44E-13 |  | 12,931 | 57,196 | 0.0065 | 0.0171 | 0.702399 |
| 89 | rs303949 | C | A | 0.00872954 | 0.00154917 | 1.75E-08 |  | 12,931 | 57,196 | -0.0153 | 0.0262 | 0.5598 |
| 90 | rs34344953 | C | T | 0.00571969 | 0.000935702 | 9.79E-10 |  | 12,931 | 57,196 | -0.0147 | 0.0164 | 0.3706 |
| 91 | rs34946857 | A | G | 0.0090773 | 0.00165246 | 3.95E-08 |  | 12,931 | 57,196 | 0.0687 | 0.0294 | 0.01962 |
| 92 | rs35429 | G | A | -0.0110645 | 0.000924745 | 5.42E-33 |  | 12,931 | 57,196 | 0.0123 | 0.0163 | 0.4523 |
| 93 | rs35432681 | C | T | 0.00648903 | 0.000923451 | 2.11E-12 |  | 12,931 | 57,196 | 0.0134 | 0.0161 | 0.4055 |
| 94 | rs35479618 | A | G | 0.0255211 | 0.00343987 | 1.18E-13 |  | 12,931 | 57,196 | 0.0404 | 0.1185 | 0.733 |
| 95 | rs35783704 | A | G | -0.0130188 | 0.00151052 | 6.77E-18 |  | 12,931 | 57,196 | -0.0169 | 0.0262 | 0.5197 |
| 96 | rs35942721 | T | C | -0.0076048 | 0.000989164 | 1.49E-14 |  | 12,931 | 57,196 | -0.0192 | 0.0178 | 0.2793 |
| 97 | rs36071027 | T | C | -0.00665162 | 0.000937598 | 1.30E-12 |  | 12,931 | 57,196 | 0.0129 | 0.0165 | 0.4334 |
| 98 | rs36174733 | G | A | -0.00677219 | 0.00116831 | 6.77E-09 |  | 12,931 | 57,196 | -0.0481 | 0.0227 | 0.0336202 |
| 99 | rs3735533 | C | T | 0.0166405 | 0.00171939 | 3.73E-22 |  | 12,931 | 57,196 | -0.0154 | 0.0308 | 0.615601 |
| 100 | rs3759582 | C | A | -0.00827667 | 0.00134949 | 8.61E-10 |  | 12,931 | 57,196 | 0.0072 | 0.0229 | 0.752 |
| 101 | rs3764769 | T | C | -0.0068207 | 0.00102118 | 2.40E-11 |  | 12,931 | 57,196 | 0.0075 | 0.0179 | 0.676299 |
| 102 | rs3785837 | A | G | 0.0074595 | 0.00107275 | 3.56E-12 |  | 12,931 | 57,196 | 0.0065 | 0.0195 | 0.738901 |
| 103 | rs3790604 | A | C | 0.0187271 | 0.00172638 | 2.05E-27 |  | 12,931 | 57,196 | 0.0424 | 0.0304 | 0.163 |
| 104 | rs3796205 | C | G | -0.00520953 | 0.000941864 | 3.18E-08 |  | 12,931 | 57,196 | -0.0097 | 0.0164 | 0.5568 |
| 105 | rs3803266 | C | G | -0.00769725 | 0.00106429 | 4.75E-13 |  | 12,931 | 57,196 | 0.0016 | 0.0183 | 0.9291 |
| 106 | rs3821843 | A | G | 0.00840648 | 0.000976299 | 7.27E-18 |  | 12,931 | 57,196 | 0.008 | 0.0175 | 0.6489 |
| 107 | rs3863105 | C | T | 0.00611387 | 0.000962822 | 2.15E-10 |  | 12,931 | 57,196 | 0.0092 | 0.0167 | 0.579201 |
| 108 | rs3867466 | C | A | 0.00773571 | 0.000999198 | 9.79E-15 |  | 12,931 | 57,196 | -0.0429 | 0.0175 | 0.0140799 |
| 109 | rs3897821 | G | A | 0.00551054 | 0.000949905 | 6.59E-09 |  | 12,931 | 57,196 | 0.0118 | 0.0167 | 0.481 |
| 110 | rs3918226 | T | C | 0.0251981 | 0.0016684 | 1.54E-51 |  | 12,931 | 57,196 | 0.0259 | 0.0536 | 0.6281 |
| 111 | rs4351668 | T | C | 0.00572754 | 0.00092755 | 6.62E-10 |  | 12,931 | 57,196 | 5.00E-04 | 0.0162 | 0.9763 |
| 112 | rs448385 | A | G | 0.00681429 | 0.000903464 | 4.61E-14 |  | 12,931 | 57,196 | -0.0139 | 0.0159 | 0.3816 |
| 113 | rs4651223 | T | C | 0.00528924 | 0.000969124 | 4.82E-08 |  | 12,931 | 57,196 | 0.0142 | 0.0169 | 0.3982 |
| 114 | rs4675682 | C | T | 0.00526118 | 0.000898999 | 4.85E-09 |  | 12,931 | 57,196 | 0.0034 | 0.0157 | 0.8297 |
| 115 | rs474328 | T | C | 0.0054289 | 0.000969633 | 2.16E-08 |  | 12,931 | 57,196 | -0.0104 | 0.017 | 0.542199 |
| 116 | rs4759062 | T | C | -0.00949665 | 0.000983707 | 4.73E-22 |  | 12,931 | 57,196 | -0.0493 | 0.0173 | 0.00442996 |
| 117 | rs4767288 | G | A | -0.00546297 | 0.000970391 | 1.81E-08 |  | 12,931 | 57,196 | 0.004 | 0.0171 | 0.816 |
| 118 | rs4775373 | C | T | -0.005689 | 0.000936434 | 1.24E-09 |  | 12,931 | 57,196 | 5.00E-04 | 0.0163 | 0.9771 |
| 119 | rs4783581 | C | G | -0.0063523 | 0.00108529 | 4.83E-09 |  | 12,931 | 57,196 | 0.0357 | 0.0195 | 0.0673101 |
| 120 | rs483465 | G | A | 0.00696887 | 0.00103533 | 1.68E-11 |  | 12,931 | 57,196 | 0.0345 | 0.0183 | 0.0589997 |
| 121 | rs4883481 | C | T | -0.00756598 | 0.000927099 | 3.32E-16 |  | 12,931 | 57,196 | -0.0121 | 0.0161 | 0.453799 |
| 122 | rs488834 | T | C | -0.00839858 | 0.0010577 | 2.02E-15 |  | 12,931 | 57,196 | 0.0504 | 0.0437 | 0.2493 |
| 123 | rs4930676 | T | C | -0.00805959 | 0.00144286 | 2.33E-08 |  | 12,931 | 57,196 | 0.0169 | 0.0275 | 0.539 |
| 124 | rs4981000 | C | T | -0.00624401 | 0.000899237 | 3.82E-12 |  | 12,931 | 57,196 | 5.00E-04 | 0.0162 | 0.9763 |
| 125 | rs537244 | T | G | -0.00687581 | 0.000907302 | 3.50E-14 |  | 12,931 | 57,196 | -0.0378 | 0.0159 | 0.0173002 |
| 126 | rs557675 | G | T | -0.00749399 | 0.00089978 | 8.17E-17 |  | 12,931 | 57,196 | 0.0104 | 0.0159 | 0.515 |
| 127 | rs55944332 | G | A | 0.00653855 | 0.00105935 | 6.73E-10 |  | 12,931 | 57,196 | -0.0067 | 0.0187 | 0.7217 |
| 128 | rs56153133 | G | A | -0.0158264 | 0.00121689 | 1.14E-38 |  | 12,931 | 57,196 | -0.0364 | 0.0216 | 0.0925891 |
| 129 | rs56271783 | C | G | 0.0123172 | 0.00217881 | 1.58E-08 |  | 12,931 | 57,196 | 0.0725 | 0.0403 | 0.0724303 |
| 130 | rs56388530 | T | C | 0.0094542 | 0.00104874 | 1.97E-19 |  | 12,931 | 57,196 | -0.0178 | 0.0184 | 0.3322 |
| 131 | rs569550 | G | T | 0.0113813 | 0.000923887 | 7.16E-35 |  | 12,931 | 57,196 | 0.0041 | 0.0288 | 0.8866 |
| 132 | rs57139556 | G | A | -0.0183069 | 0.00173904 | 6.49E-26 |  | 12,931 | 57,196 | -0.0075 | 0.0308 | 0.8068 |
| 133 | rs59980837 | T | G | 0.030137 | 0.00339588 | 7.02E-19 |  | 12,931 | 57,196 | -0.0874 | 0.0633 | 0.1672 |
| 134 | rs6026739 | T | A | 0.0191373 | 0.00139985 | 1.51E-42 |  | 12,931 | 57,196 | 0.0127 | 0.0241 | 0.6002 |
| 135 | rs6031431 | G | A | 0.00725662 | 0.000905914 | 1.14E-15 |  | 12,931 | 57,196 | -0.0131 | 0.0158 | 0.4055 |
| 136 | rs604723 | C | T | 0.0145019 | 0.00101129 | 1.23E-46 |  | 12,931 | 57,196 | -0.0021 | 0.0175 | 0.9064 |
| 137 | rs6090907 | A | G | -0.00750749 | 0.00126055 | 2.59E-09 |  | 12,931 | 57,196 | 0.0085 | 0.0219 | 0.6968 |
| 138 | rs6108168 | A | C | -0.0108666 | 0.0010302 | 5.19E-26 |  | 12,931 | 57,196 | 0.0322 | 0.018 | 0.0732504 |
| 139 | rs61772592 | G | A | 0.00911074 | 0.00136661 | 2.62E-11 |  | 12,931 | 57,196 | 0.0096 | 0.0232 | 0.6798 |
| 140 | rs62039768 | A | C | 0.0108998 | 0.00153381 | 1.19E-12 |  | 12,931 | 57,196 | -0.0407 | 0.0277 | 0.1413 |
| 141 | rs62043959 | C | A | -0.00751939 | 0.00100461 | 7.16E-14 |  | 12,931 | 57,196 | 0.007 | 0.0181 | 0.7011 |
| 142 | rs62189015 | C | T | -0.00914529 | 0.00150035 | 1.09E-09 |  | 12,931 | 57,196 | 0.0137 | 0.0261 | 0.5999 |
| 143 | rs62481856 | A | G | 0.00996236 | 0.00112579 | 8.81E-19 |  | 12,931 | 57,196 | -0.0055 | 0.0195 | 0.7771 |
| 144 | rs6271 | T | C | -0.013259 | 0.00171351 | 1.01E-14 |  | 12,931 | 57,196 | -0.0653 | 0.0554 | 0.2386 |
| 145 | rs6441207 | T | C | 0.0056617 | 0.000915044 | 6.12E-10 |  | 12,931 | 57,196 | 0.0067 | 0.016 | 0.676299 |
| 146 | rs6595833 | T | C | 0.00735974 | 0.000914798 | 8.61E-16 |  | 12,931 | 57,196 | -0.0064 | 0.016 | 0.69 |
| 147 | rs6666703 | T | C | -0.00549363 | 0.000990499 | 2.92E-08 |  | 12,931 | 57,196 | -0.0117 | 0.0172 | 0.4975 |
| 148 | rs675605 | C | G | -0.00548512 | 0.000991253 | 3.14E-08 |  | 12,931 | 57,196 | 0.0283 | 0.0182 | 0.1208 |
| 149 | rs681343 | T | C | 0.00566235 | 0.000896045 | 2.63E-10 |  | 12,931 | 57,196 | 0.0386 | 0.0173 | 0.02594 |
| 150 | rs682709 | G | C | 0.00687977 | 0.000964778 | 9.97E-13 |  | 12,931 | 57,196 | -0.0305 | 0.0168 | 0.0687306 |
| 151 | rs6848906 | C | T | -0.0106107 | 0.0012506 | 2.17E-17 |  | 12,931 | 57,196 | -0.029 | 0.0217 | 0.1808 |
| 152 | rs6855246 | G | A | -0.0126807 | 0.00170449 | 1.01E-13 |  | 12,931 | 57,196 | -0.0312 | 0.03 | 0.2976 |
| 153 | rs696 | T | C | 0.00517754 | 0.000932697 | 2.84E-08 |  | 12,931 | 57,196 | 0.014 | 0.0165 | 0.3956 |
| 154 | rs6961048 | G | C | 0.0108579 | 0.00148711 | 2.85E-13 |  | 12,931 | 57,196 | -0.006 | 0.0259 | 0.8164 |
| 155 | rs7009973 | G | A | 0.00547402 | 0.00096157 | 1.25E-08 |  | 12,931 | 57,196 | -0.0186 | 0.0169 | 0.2704 |
| 156 | rs706406 | A | C | 0.00563334 | 0.000921203 | 9.64E-10 |  | 12,931 | 57,196 | 0.0103 | 0.016 | 0.5188 |
| 157 | rs71371126 | A | G | 0.00530599 | 0.000946641 | 2.08E-08 |  | 12,931 | 57,196 | 0.032 | 0.0167 | 0.05434 |
| 158 | rs71631245 | T | C | 0.0164647 | 0.00289133 | 1.24E-08 |  | 12,931 | 57,196 | 0.0221 | 0.0538 | 0.681999 |
| 159 | rs7187250 | A | C | 0.0091922 | 0.00091842 | 1.40E-23 |  | 12,931 | 57,196 | 0.0436 | 0.0158 | 0.00579095 |
| 160 | rs7237714 | G | A | 0.00869762 | 0.00132558 | 5.33E-11 |  | 12,931 | 57,196 | 0.0184 | 0.0227 | 0.419 |
| 161 | rs7258382 | C | T | -0.0115164 | 0.00120577 | 1.28E-21 |  | 12,931 | 57,196 | 0.0041 | 0.025 | 0.8705 |
| 162 | rs72677850 | A | G | -0.0234357 | 0.00338669 | 4.52E-12 |  | 12,931 | 57,196 | -0.0646 | 0.0638 | 0.3117 |
| 163 | rs7278003 | C | T | 0.00510515 | 0.000903796 | 1.62E-08 |  | 12,931 | 57,196 | -0.0026 | 0.0159 | 0.8722 |
| 164 | rs72801474 | A | G | -0.0092404 | 0.00155365 | 2.72E-09 |  | 12,931 | 57,196 | -0.0274 | 0.0291 | 0.3478 |
| 165 | rs72831343 | G | T | -0.0201099 | 0.00127261 | 3.01E-56 |  | 12,931 | 57,196 | -0.0291 | 0.0227 | 0.2002 |
| 166 | rs72915163 | T | C | 0.00795953 | 0.00103729 | 1.68E-14 |  | 12,931 | 57,196 | 0.0101 | 0.0185 | 0.5836 |
| 167 | rs73046792 | A | G | -0.00755912 | 0.00121349 | 4.69E-10 |  | 12,931 | 57,196 | -0.0136 | 0.0221 | 0.5376 |
| 168 | rs73050466 | A | G | -0.016241 | 0.00266298 | 1.07E-09 |  | 12,931 | 57,196 | -0.1666 | 0.1653 | 0.3136 |
| 169 | rs73098804 | A | T | 0.00819459 | 0.00133989 | 9.60E-10 |  | 12,931 | 57,196 | 0.0044 | 0.0235 | 0.853 |
| 170 | rs73099903 | T | C | 0.00885322 | 0.00161545 | 4.25E-08 |  | 12,931 | 57,196 | -0.0118 | 0.0289 | 0.683701 |
| 171 | rs7340705 | C | T | 0.00677796 | 0.00096552 | 2.22E-12 |  | 12,931 | 57,196 | 0.0108 | 0.0167 | 0.5203 |
| 172 | rs73693253 | T | C | -0.00884618 | 0.00146053 | 1.39E-09 |  | 12,931 | 57,196 | 0.0482 | 0.0258 | 0.0618999 |
| 173 | rs73728279 | T | G | 0.00778142 | 0.000997953 | 6.32E-15 |  | 12,931 | 57,196 | 0.0097 | 0.0177 | 0.5837 |
| 174 | rs740047 | T | C | 0.00657594 | 0.00112108 | 4.47E-09 |  | 12,931 | 57,196 | 0.0116 | 0.0195 | 0.5517 |
| 175 | rs74439044 | C | T | 0.0133165 | 0.00151444 | 1.46E-18 |  | 12,931 | 57,196 | -0.004 | 0.0266 | 0.8802 |
| 176 | rs7498127 | A | G | -0.00613362 | 0.000900437 | 9.64E-12 |  | 12,931 | 57,196 | 0.0092 | 0.0162 | 0.5717 |
| 177 | rs75523587 | A | T | 0.0068243 | 0.00116183 | 4.26E-09 |  | 12,931 | 57,196 | 0.0028 | 0.0371 | 0.9401 |
| 178 | rs76038906 | T | G | 0.0169645 | 0.0024547 | 4.81E-12 |  | 12,931 | 57,196 | 0.0043 | 0.0424 | 0.9198 |
| 179 | rs76452347 | T | C | -0.00959303 | 0.00115513 | 1.00E-16 |  | 12,931 | 57,196 | -0.0116 | 0.0218 | 0.595 |
| 180 | rs7700842 | C | T | -0.0112029 | 0.00092786 | 1.45E-33 |  | 12,931 | 57,196 | -0.02 | 0.0162 | 0.2166 |
| 181 | rs7763350 | C | A | 0.00831914 | 0.000958994 | 4.14E-18 |  | 12,931 | 57,196 | 0.0158 | 0.0166 | 0.3389 |
| 182 | rs7777545 | C | T | -0.00624618 | 0.00091476 | 8.60E-12 |  | 12,931 | 57,196 | 0.0176 | 0.0161 | 0.2738 |
| 183 | rs77924615 | A | G | -0.0119344 | 0.00113999 | 1.20E-25 |  | 12,931 | 57,196 | 0.0206 | 0.02 | 0.3034 |
| 184 | rs7805240 | T | C | 0.00567977 | 0.000915438 | 5.49E-10 |  | 12,931 | 57,196 | -0.0202 | 0.0161 | 0.2076 |
| 185 | rs78307470 | G | A | -0.0118378 | 0.00174445 | 1.15E-11 |  | 12,931 | 57,196 | -0.026 | 0.0296 | 0.379 |
| 186 | rs7831859 | C | T | -0.00512481 | 0.000908581 | 1.70E-08 |  | 12,931 | 57,196 | -0.0179 | 0.016 | 0.2621 |
| 187 | rs7838131 | A | G | -0.00770089 | 0.000908849 | 2.39E-17 |  | 12,931 | 57,196 | 0.0159 | 0.0161 | 0.3232 |
| 188 | rs7844259 | A | G | 0.00656456 | 0.00103454 | 2.22E-10 |  | 12,931 | 57,196 | -0.0055 | 0.0181 | 0.762801 |
| 189 | rs7911644 | T | C | 0.00757404 | 0.000956423 | 2.39E-15 |  | 12,931 | 57,196 | 0.0179 | 0.0166 | 0.2807 |
| 190 | rs79384779 | T | C | 0.00788049 | 0.00128513 | 8.68E-10 |  | 12,931 | 57,196 | -0.0116 | 0.0234 | 0.621199 |
| 191 | rs8002514 | A | G | 0.00625225 | 0.00105781 | 3.41E-09 |  | 12,931 | 57,196 | -0.0021 | 0.0185 | 0.9112 |
| 192 | rs8027450 | T | C | 0.0117734 | 0.000963142 | 2.32E-34 |  | 12,931 | 57,196 | 0.0173 | 0.02 | 0.3862 |
| 193 | rs8067500 | T | C | 0.00531315 | 0.000922204 | 8.34E-09 |  | 12,931 | 57,196 | 0.0234 | 0.0174 | 0.1803 |
| 194 | rs8070737 | T | G | 0.00717181 | 0.00116025 | 6.36E-10 |  | 12,931 | 57,196 | 0.0443 | 0.02 | 0.0270701 |
| 195 | rs8077276 | A | G | -0.0092985 | 0.000931271 | 1.78E-23 |  | 12,931 | 57,196 | -0.0242 | 0.0177 | 0.1727 |
| 196 | rs8093196 | T | G | 0.00697194 | 0.000958126 | 3.42E-13 |  | 12,931 | 57,196 | -0.0084 | 0.0165 | 0.6122 |
| 197 | rs8243 | A | C | -0.00517753 | 0.00092281 | 2.02E-08 |  | 12,931 | 57,196 | 0.0093 | 0.0163 | 0.5703 |
| 198 | rs906754 | A | G | -0.00652183 | 0.00103439 | 2.88E-10 |  | 12,931 | 57,196 | 0.0138 | 0.0181 | 0.4458 |
| 199 | rs9286351 | G | A | 0.00605282 | 0.000911548 | 3.13E-11 |  | 12,931 | 57,196 | 0.0093 | 0.0159 | 0.5575 |
| 200 | rs9375459 | T | C | 0.0120377 | 0.000903121 | 1.57E-40 |  | 12,931 | 57,196 | 0.0033 | 0.0158 | 0.8321 |
| 201 | rs9479072 | T | C | -0.00533388 | 0.000914177 | 5.39E-09 |  | 12,931 | 57,196 | 0.0184 | 0.0159 | 0.2484 |
| 202 | rs951914 | C | G | 0.00901473 | 0.000999211 | 1.85E-19 |  | 12,931 | 57,196 | -0.0299 | 0.0175 | 0.0877607 |
| 203 | rs9836592 | T | C | 0.00628536 | 0.000950902 | 3.85E-11 |  | 12,931 | 57,196 | 0.018 | 0.0177 | 0.31 |
| 204 | rs9943599 | T | C | 0.00717277 | 0.000908738 | 2.95E-15 |  | 12,931 | 57,196 | -0.0178 | 0.016 | 0.2649 |

SNP, single nucleotide polymorphism; EA, effect allele; OA, other allele; SE, standard error; T2D, type 2 diabetes.

**Supplementary Table S2** The results of horizontal pleiotropy analysis.

| **Exposure** | **Outcome** | **Egger_intercept** | **SE** | ***p* value** |
| --- | --- | --- | --- | --- |
| PTSD | T2D | 0.004252925 | 0.005440042 | 0.436350307 |
| PTSD | Obesity | 0.005067615 | 0.003349006 | 0.13359156 |
| PTSD | Hypertension | -2.65E-05 | 0.000303958 | 0.930674663 |
| PTSD | Hyperlipidemia | 0.001578566 | 0.001600576 | 0.326516305 |
| PTSD | Alcohol consumption | -0.013572255 | 0.006807413 | 0.050022023 |
| PTSD | Smoking status | 0.00025947 | 0.000448401 | 0.564333312 |
| Obesity | T2D | -0.004828046 | 0.007643001 | 0.530854604 |
| Hypertension | T2D | -0.000669587 | 0.004253828 | 0.875080384 |

PTSD, post-traumatic stress disorder; T2D, type 2 diabetes.

**Supplementary Table S3** The results of heterogeneity analysis.

| **Exposure** | **Outcome** | **Method** | **Q** | **Q_df** | **Q_*p* val** |
| --- | --- | --- | --- | --- | --- |
| PTSD | T2D | MR Egger | 95.99941894 | 92 | 0.367052032 |
| PTSD | T2D | Inverse variance weighted | 96.63717067 | 93 | 0.377504606 |
| PTSD | Obesity | MR Egger | 100.9297769 | 94 | 0.293961154 |
| PTSD | Obesity | Inverse variance weighted | 103.3882579 | 95 | 0.261232527 |
| PTSD | Hypertension | MR Egger | 96.29257666 | 87 | 0.232397183 |
| PTSD | Hypertension | Inverse variance weighted | 96.30100199 | 88 | 0.255606647 |
| PTSD | Hyperlipidemia | MR Egger | 78.94566061 | 95 | 0.882690864 |
| PTSD | Hyperlipidemia | Inverse variance weighted | 79.91834719 | 96 | 0.8817409 |
| PTSD | Alcohol consumption | MR Egger | 77.21699967 | 71 | 0.286781751 |
| PTSD | Alcohol consumption | Inverse variance weighted | 81.54009126 | 72 | 0.20685407 |
| PTSD | Smoking status | MR Egger | 93.5430157 | 86 | 0.271048348 |
| PTSD | Smoking status | Inverse variance weighted | 93.90722814 | 87 | 0.287525518 |
| Obesity | T2D | MR Egger | 52.48696348 | 44 | 0.178162018 |
| Obesity | T2D | Inverse variance weighted | 52.96297101 | 45 | 0.193853249 |
| Hypertension | T2D | MR Egger | 253.1324223 | 202 | 0.008451293 |
| Hypertension | T2D | Inverse variance weighted | 253.1634715 | 203 | 0.00958411 |

PTSD, post-traumatic stress disorder; T2D, type 2 diabetes.
